# FAM114A1 influences cardiac pathological remodeling by regulating angiotensin II signaling

**DOI:** 10.1172/jci.insight.152783

**Published:** 2022-07-08

**Authors:** Kadiam C. Venkata Subbaiah, Jiangbin Wu, Wai Hong Wilson Tang, Peng Yao

**Affiliations:** 1 Aab Cardiovascular Research Institute, Department of Medicine, University of Rochester School of Medicine & Dentistry, Rochester, New York, USA.; 2 Department of Cardiovascular Medicine, Cleveland Clinic, Cleveland, Ohio, USA.; 3 Department of Biochemistry & Biophysics,; 4 The Center for RNA Biology, and; 5 The Center for Biomedical Informatics, University of Rochester School of Medicine & Dentistry, Rochester, New York, USA.

**Keywords:** Cardiology, Cardiovascular disease, Fibrosis, Heart failure

## Abstract

Cardiac pathological remodeling, a primary contributor to heart failure (HF) and death, is an important target for HF therapy. However, the signaling pathways that govern cardiac remodeling are not fully elucidated. Here, we found that a functionally unannotated human myocardial infarction–associated (MI-associated) gene, family with sequence similarity 114 member A1 (*FAM114A1*), is induced in failing human and mouse hearts compared with nonfailing hearts. Homozygous KO of *Fam114a1* (*Fam114a1*^–/–^) in the mouse genome reduces cardiomyocyte hypertrophy, inflammation, and cardiac fibrosis while restoring cardiac function in angiotensin II–induced (Ang II–induced) and MI-induced HF mouse models. Cardiac fibroblasts (CFs) exhibit the highest FAM114A1 expression among different cardiac cell types. FAM114A1 is a critical autonomous factor for CF proliferation, activation, and migration. Mechanistically, FAM114A1 interacts with angiotensin receptor–associated protein (AGTRAP) and regulates the expression of angiotensin type 1 receptor (AT1R) and downstream Ang II signaling transduction, and it subsequently influences profibrotic response. Our results indicate that FAM114A1 regulates Ang II signaling, thereby activating CFs and other cardiac cells and augmenting pathological cardiac remodeling. These findings provide potentially novel insights into the regulation of cardiac remodeling and identify FAM114A1 as a therapeutic target for the treatment of heart disease.

## Introduction

Heart failure (HF) is a leading cause of morbidity and mortality across the globe (1). The development of HF in chronic stresses such as hypertension and myocardial infarction (MI) is characterized by complex changes in the structure and function of the heart at the cellular and molecular levels. This cardiac pathological remodeling process ultimately leads to cardiomyocyte (CM) hypertrophy, chamber dilatation, contractile dysfunction, and HF. Furthermore, massive amounts of clinical and experimental data have demonstrated that chronic activation of cardiac fibroblasts (CFs) and accumulation of cardiac fibrosis adversely affect cardiac compliance, cause diastolic dysfunction (2, 3), and lead to arrhythmia and sudden death (4, 5). However, effective antiremodeling therapeutics, especially antifibrosis drugs, are still limited (6, 7). Therefore, understanding the underpinning mechanisms of cardiac remodeling and identifying potentially novel modalities to modulate this process is of a high scientific impact and therapeutic potential.

In response to cardiac injuries during pathological remodeling, resident CFs proliferate following an MI ischemic insult (8). Increased CFs secrete more extracellular matrix (ECM) and contribute to the immune response (9, 10). The initial scar formation is a beneficial response that contributes to the replacement of dead CMs upon MI injury and prevents cardiac rupture. Then, resident CFs undergo a cell state transition into myofibroblasts (MFs) with increased ECM production, proliferation, and contractility (11), thereby driving persistent cardiac pathological fibrosis when left unchecked (9). At the molecular level, angiotensin II (Ang II), a critical neurohormonal ligand constituent of the renin-angiotensin system (RAS), plays a vital role in cardiac fibrosis by promoting CF proliferation and increasing the production of proinflammatory cytokines and ECM proteins through the activation of Ang II type 1 receptor (AT1R) (12, 13). The function of circulating RAS has been extensively studied. What remains elusive is the regulatory mechanism of tissue- and cell type–oriented RAS involved in the long-term effects of chronic RAS activation associated with local organ damage in the cardiac pathophysiology (14). Ang II activates profibrogenic cascades such as TGF-β/SMAD, ERK1/2, AKT (protein kinase B), and p38 MAPK signal transduction during the pathogenesis of cardiac fibrosis (15–17). The pathophysiological effects of Ang II can rewire the transcriptome of CFs to promote the activation of fibroblasts with enhanced ability to proliferate, migrate, secrete proinflammatory mediators, and produce ECM proteins (12, 13, 15, 16). Indeed, the first-line treatment for HF relies on angiotensin-converting enzyme (ACE) inhibitors and Ang II receptor blockers, which show promise in blunting cardiac fibrosis (2, 18, 19). This treatment, however, is limited due to its adverse effects. Thus, we still need to identify novel Ang II signaling regulators for targeting in order to advance the therapeutic interventions and develop effective therapies against HF.

In Ang II signaling transduction, AT1R serves as a control point for regulating the Ang II effects, and AT1R overactivation leads to cardiac pathogenesis (20, 21). AT1R-associated protein (AGTRAP) selectively inhibits AT1R overactivation and suppresses the Ang II–induced cardiac remodeling in mouse HF models (22, 23). CM-specific AGTRAP transgenic mice exhibit significantly diminished Ang II–induced cardiac remodeling due to an increased AGTRAP/AT1R ratio, AT1R internalization, and diminished AT1R downstream effects (24). Interestingly, AGTRAP expression was significantly reduced in failing mouse hearts (24). However, the molecular mechanism is poorly understood (13). A yeast 2-hybrid screen identified AGTRAP as an interacting protein of family with sequence similarity 114 member A1 (FAM114A1) (25). Intriguingly, 3 human genome-wide association studies (GWAS) have discovered that a single nucleotide polymorphism (SNP) rs1873197 in *FAM114A1* genomic locus is associated with MI and coronary artery disease (CAD) (26–28) (Supplemental Figure 1, A and B; supplemental material available online with this article; https://doi.org/10.1172/jci.insight.152783DS1). However, the function and mechanism of FAM114A1 in cardiac pathophysiology and general biology are unknown, to date. Therefore, we investigated the role of FAM114A1 in heart disease and its potential as a therapeutic target for HF treatment.

This work has revealed the role of FAM114A1 in the cardiac pathological remodeling process, especially in CFs and during cardiac fibrosis. FAM114A1 expression is robustly elevated in human and mouse failing hearts, highly enriched in CFs, and correlated with the expression of profibrotic collagens. *Fam114a1*^–/–^ mouse hearts display greater resistance to Ang II– and MI-induced CM hypertrophy, cardiac inflammation, fibrosis, and dysfunction than hearts from WT mice. Mechanistically, cardiac stress–induced FAM114A1 binds to AGTRAP and increases AT1R expression, amplifying Ang II signaling. Transcriptomic profiling of *Fam114a1-*null CFs revealed multiple downstream effectors involved in ECM remodeling, such as Adamts15 required for CF activation and migration. This study confirms the safety of complete inactivation of FAM114A1 to be used as a potential therapeutic approach to treat cardiac diseases.

## Results

### FAM114A1 expression is induced in CFs of human and mouse failing hearts.

**Figure 1:**
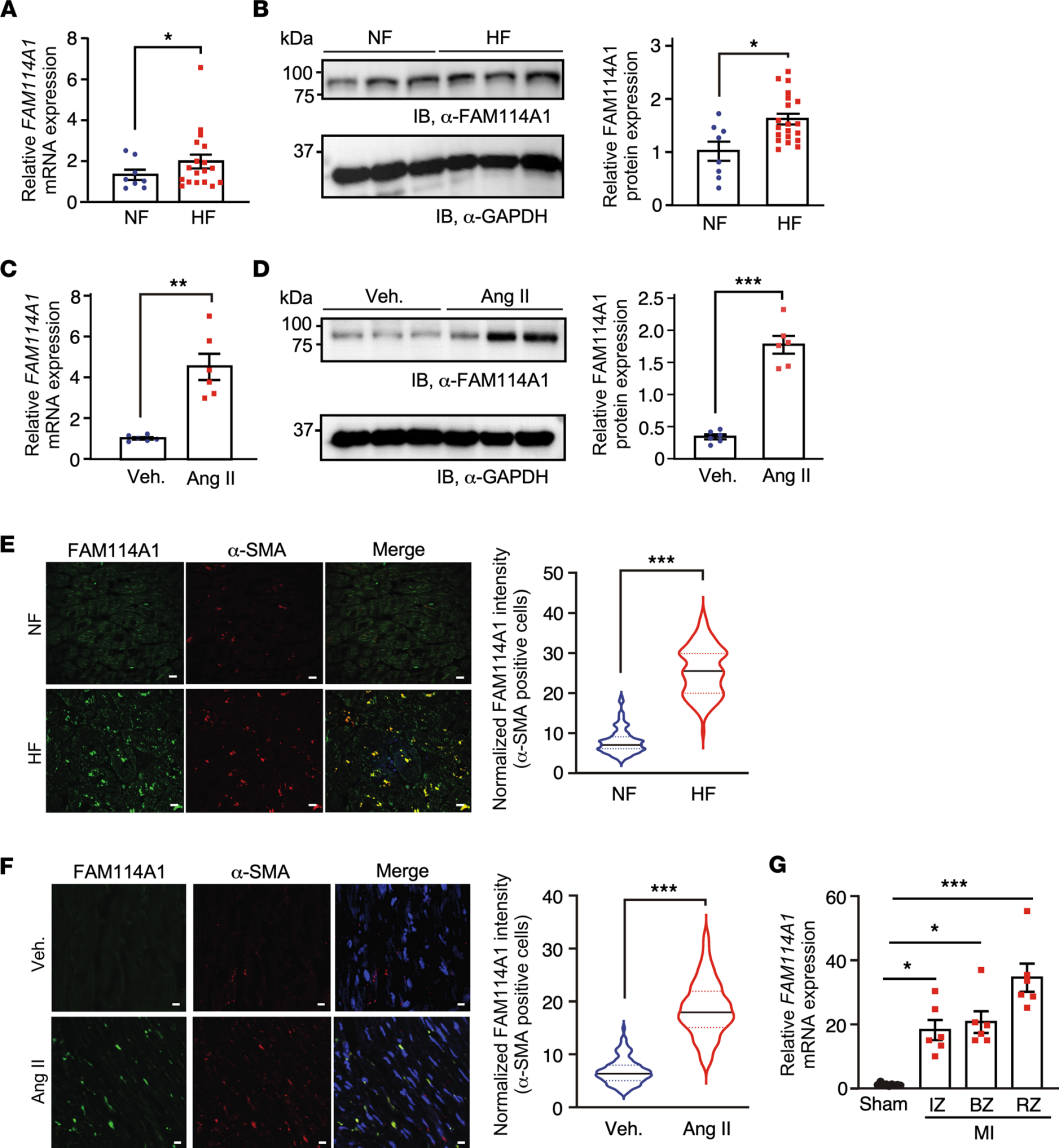
Increased FAM114A1 expression in activated myofibroblasts in human and mouse failing hearts. (**A**) *FAM114A1* mRNA expression is increased in failing human hearts (*n* = 18) compared with nonfailure hearts (*n* = 8). 18S rRNA was used as a normalizer. (**B**) FAM114A1 protein expression is increased in failing human hearts (*n* = 20) compared with nonfailure hearts (*n* = 8). GAPDH protein was used as a normalizer. (**C** and **D**) *FAM114A1* mRNA and protein expression is induced in the hearts from mice with Ang II infusion (4 weeks) compared with hearts from vehicle-treated mice (*n* = 6 for both groups). 18S rRNA and *Gapdh* mRNA were used as normalizers for mRNA and protein quantification, respectively. (**E** and **F**) IF analysis and quantification of FAM114A1 protein expression in human failing hearts and Ang II–treated mouse heart tissue sections. *n* = 100–115 cells were counted from multiple sections from murine hearts (*n* = 6). Scale bar: 10 μm (**E**); 5 μm (**F**). (**G**) *Fam114a1* mRNA expression is induced in the hearts from mice with MI surgery at infarct zone (IZ), border zone (BZ), and remote zone (RZ) compared with the hearts with Sham surgery (20 days) (*n* = 6). Data are presented as mean ± SEM. Comparisons of means between 2 groups were performed by unpaired 2-tailed Mann Whitney *U* test for data nonnormally distributed for **A**, **E**, and **F**, and unpaired Student *t* test for data normally distributed for **B**–**D**. Comparisons of means between > 2 groups were performed by 1-way ANOVA with Tukey’s multiple-comparison test for **G**. *P < 0.05; **P < 0.01; ***P <0.001.

Genetic associations of the *FAM114A1* gene with MI and CAD imply its potential impact on cardiac pathogenesis (26–28) (Supplemental Figure 1, A and B). Prior studies show that miR-574 is consistently induced in the human and mouse heart in response to pathological cardiac remodeling (29–32). The host gene of miR-574, *FAM114A1*, is coexpressed with the embedded intronic miRNA in the brain (33). Therefore, we attempted to examine whether cardiac stresses also induce FAM114A1 expression. Using human HF patient samples, we found that FAM114A1 was induced by ~50% at both mRNA and protein levels in failing human hearts compared with nonfailure donor hearts (Figure 1, A and B). To explore the gene expression of FAM114A1 in mouse HF, we infused the WT mice with Ang II (1.4 mg/Kg/day) for 4 weeks to mimic human hypertensive cardiomyopathy. Immunoblotting and qPCR showed that Ang II induced the expression of FAM114A1 by approximately 4-fold at both mRNA and protein levels in murine hearts (Figure 1, C and D).

Immunofluorescence (IF) studies revealed that FAM114A1 protein expression was significantly increased and strongly colocalized with α-SMA^+^ cells (probably activated MFs) but not colocalized with CMs in failing human hearts compared with nonfailure donor hearts (Figure 1E and Supplemental Figure 1C). We confirmed that severe fibrosis existed in failing human hearts, as indicated by high collagen deposition compared with nonfailure hearts (Supplemental Figure 1D). Pearson correlation coefficient analysis was performed to investigate whether FAM114A1 expression is associated with cardiac fibrosis during HF. The expression of *FAM114A1* mRNA showed a strong positive correlation with the expression level of collagen mRNAs such as *COL1A1* (*R* = 0.743) and *COL3A1* (*R* = 0.861) but not with that of *ANP* and *BNP* (Supplemental Figure 1E). An independent data set of human samples also shows increased expression of *FAM114A1* mRNA in the failing hearts of patients with idiopathic dilated cardiomyopathy (IDCM) and ischemic cardiomyopathy (ICM) compared with healthy participants (Supplemental Figure 1F). Consistently, FAM114A1 protein was colocalized with α-SMA in Ang II–treated murine hearts, suggesting high-level expression of FAM114A1 in activated MFs (Figure 1F). Besides, *FAM114A1* mRNA was highly induced in the ischemic myocardium in the mouse after MI surgery (Figure 1G). Moreover, FAM114A1 protein was significantly induced in the mouse ischemic myocardium (Supplemental Figure 1G). To confirm the FAM114A1^+^ cells are MFs rather than smooth muscle cells (SMCs), we performed costaining of α-SMA and FAM114A1 with vimentin, a CF marker that is not expressed in SMCs. FAM114A1 was highly enriched in CFs and MFs in both human and mouse heart tissue sections (Supplemental Figure 1, H and I, left panel). We further confirmed partial colocalization of FAM114A1 with another MF-expressing marker protein, Periostin, in tissue sections of both failing human hearts and mouse MI hearts (Supplemental Figure 1, H and I, right panel). Western blot measurement of the protein expression in isolated CFs from MI and sham-treated murine hearts further confirmed the high expression of FAM114A1 in CFs under cardiac stress conditions (Supplemental Figure 1J). We next searched for potential transcription factors (TFs) required for Ang II–activated FAM114A1 expression. We overlapped TFs identified in binding FAM114A1 DNA promoter region from TFBS database (TFBSDB) and well-established Ang II signaling–activated TFs (34) and found 5 candidates, including NFAT, FOXO3A, MEF2, NF-κB, and SMAD1 (Supplemental Figure 1K and Supplemental Table 1). Among these 5 candidate TFs, NFAT has been reported to be involved in Ang II activated CF-to-MF transition (35–39). The NFAT family consists of 5 gene members: *NFATC1* (or *NFAT2*), *NFATC2* (or *NFAT1*), *NFATC3* (or *NFAT4*), *NFATC4* (or *NFAT3*), and *NFAT5*. We knocked down each *Nfat* gene in primary mouse CFs (PMCFs) (not shown) and found that *Fam114a1* mRNA was significantly reduced when *Nfatc3* was knocked down, suggesting that NFATC3 is required for Ang II–activated transcription of *Fam114A1* (Supplemental Figure 1L). This observation is consistent with previous reports of Ang II activation of NFATC3 in CFs (35–37). Altogether, these findings indicate a strong positive correlation between FAM114A1 expression and CF activation in both human and mouse HF, and they show that NFATC3 contributes to the induction of FAM114A1 in CFs.

### Fam114a1 deficiency in mice reduces cardiac fibrosis, relieves CM pathology, and protects cardiac function under pathogenic stresses.

To determine the role of FAM114A1 in cardiac pathogenesis, we obtained *Fam114a1*–global KO mice created by the CRISPR-Cas9 technology (Supplemental Figure 2, A and B). The homozygous *Fam114a1*^–/–^ mice were viable, fertile, and normal in body weight and behavior. *Fam114a1* mRNA with a premature stop codon was detected by probes spanning exons 1–2 and 5–6 but not exons 2–3, suggesting a successful exon 3 deletion at both DNA and mRNA levels (Supplemental Figure 2C). FAM114A1 protein expression was abolished in the heart of *Fam114a1*^–/–^ mice (Supplemental Figure 2D). Thus, FAM114A1 is a nonessential gene for organismal viability, and the loss of both alleles does not cause obvious morphological changes in the mouse body at baseline.

**Figure 2:**
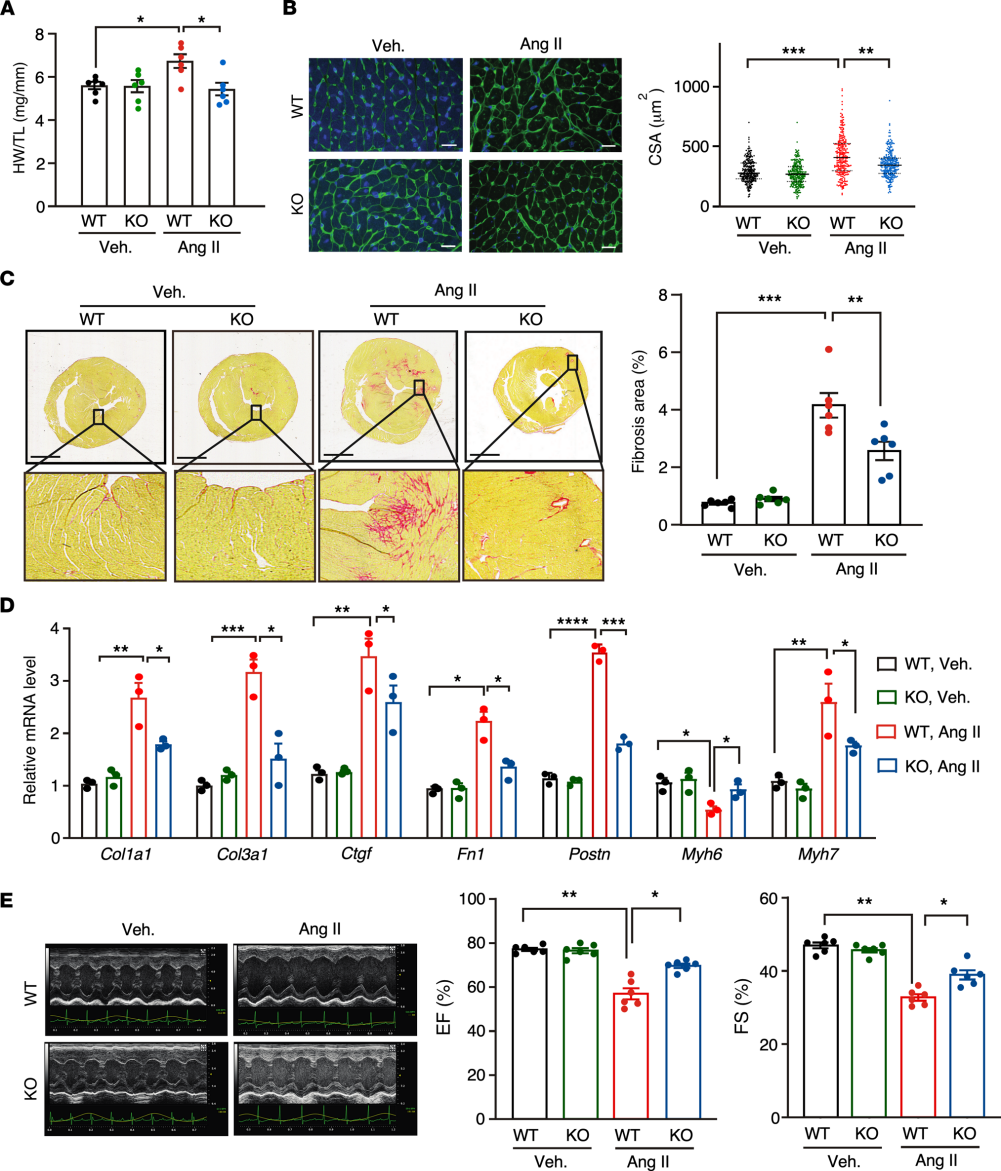
Deletion of *FAM114A1* mitigates Ang II–induced cardiac remodeling and heart failure in mice. (**A**) *Fam114a1*^–/–^ mice show reduced HW/TL (heart weight/tibia length) ratio after 4 weeks of Ang II infusion compared with WT mice. *n* = 6 for each group (male mice). (**B**) WGA staining of heart tissue sections of WT and *Fam114a1*^–/–^ mice under Ang II or vehicle treatment. Cross-sectional area of CMs was measured and quantified. *n* = 6 hearts per group with 250–270 CMs measured per heart. Scale bar: 20 μm. (**C**) Picrosirius red staining of heart tissue sections of WT and *Fam114a1*^–/–^ mice under Ang II or vehicle treatment. Scale bar: 1 mm. *n* = 6 for each group. (**D**) qPCR measurement of cardiac fibrosis and hypertrophy marker gene expression in WT and *Fam114a1*^–/–^ hearts after 4 weeks of Ang II versus vehicle infusion. *Rpl30* mRNA was used as a normalizer. *n* = 3 for each group. (**E**) Representative echocardiographic images suggest improved cardiac function in *Fam114a1*^–/–^ mice compared with WT mice after Ang II or vehicle infusion. Quantification of ejection fraction (EF) and fractional shortening (FS) was shown. *n* = 6 for each group. Data are presented as mean ± SEM. Statistical significance was confirmed by 2-way ANOVA with Tukey’s multiple-comparison tests for **A**–**E**. *P < 0.05; **P < 0.01; ***P <0.001;.****P <0.0001.

Given the induction of FAM114A1 expression in human and mouse failing hearts, we sought to determine the pathophysiological effects of FAM114A1 deletion on the murine heart under cardiac remodeling in vivo. We first used Ang II to treat mice via osmotic minipump implantation (1.4 mg/Kg/day) for 4 weeks. The hearts of WT and *Fam114a1*^–/–^ mice were comparable in size at baseline (Supplemental Figure 2E), as indicated by heart weight/tibia length (HW/TL) and HW/body weight (HW/BW) ratios (Figure 2A and Supplemental Figure 2F), as well as wheat germ agglutinin (WGA) staining (Figure 2B). Picrosirius red staining showed that *Fam114a1*^–/–^ mice had much less cardiac fibrosis (reduced by 38.2%) than WT mice after Ang II treatment, suggesting that *Fam114a1*^–/–^ mice are more resistant to cardiac remodeling in response to Ang II stimulation than WT mice (Figure 2C). Expression of multiple fibrosis marker genes (*Col1a1*, *Col3a1*, *Ctgf*, *Fn1*, and *Postn*) was not significantly altered in *Fam114a1*^–/–^ mice at baseline (Figure 2D). However, the expression of these genes was significantly lower in *Fam114a1*^–/–^ versus WT mice after Ang II treatment (Figure 2D). Also, the expression of cardiac hypertrophy marker genes (*Myh6*, *Myh7*) was partially normalized in *Fam114a1*^–/–^ mouse hearts compared with those of WT mice upon Ang II treatment (Figure 2D). Echocardiographic examinations showed a significant decrease in the left ventricular ejection fraction (EF), fractional shortening (FS), and chamber volume in Ang II–treated WT mice compared with vehicle-treated mice (Figure 2E and Supplemental Table 2). In contrast, cardiac function was markedly restored in *Fam114a1*^–/–^ mice with slightly reduced heart rates compared with WT mice after Ang II treatment (Figure 2E and Supplemental Table 2).

**Figure 3:**
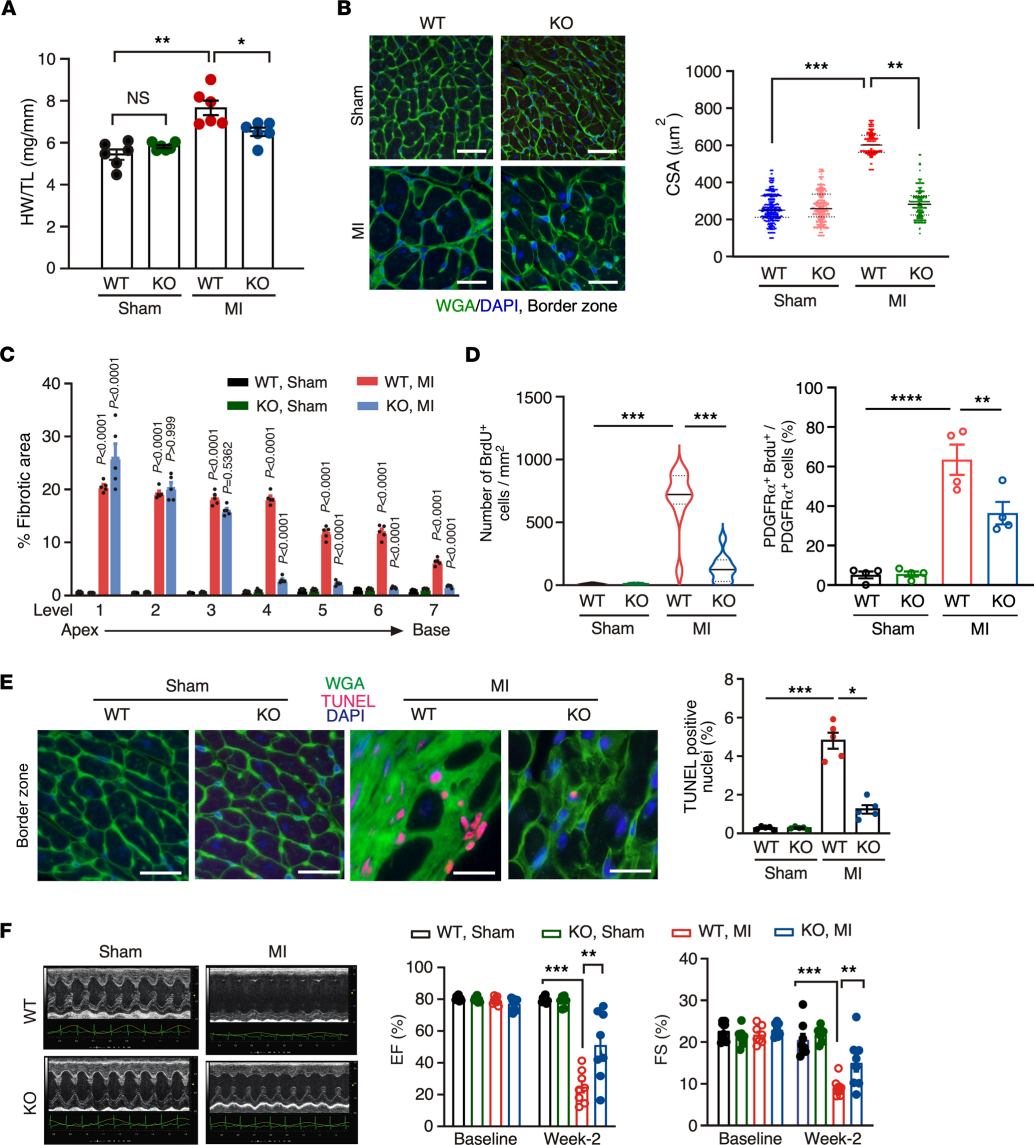
Deletion of *FAM114A1* reduces cardiac remodeling and improves cardiac function after MI surgery. (**A**) *Fam114a1*^–/–^ mice show reduced HW/TL ratio 20 days after MI surgery compared with WT mice. *n* = 6 for each group (male and female mice). (**B**) WGA staining of heart tissue sections of WT and *Fam114a1*^–/–^ mice 20 days after MI surgery. *n* = 6 (each group) with 100–150 CMs in the border zone measured per heart. Scale bar: 20 μm. (**C**) Quantification of Picrosirius red staining of heart tissue sections of WT and *Fam114a1*^–/–^ mice 20 days after MI surgery. *n* = 5 for each group. (**D**) Quantification of PDGFRα protein expression (% of the area) and percentage of PDGFRα^+^BrdU^+^ cells in BrdU^+^ cells in heart tissue sections of WT and *Fam114a1*^–/–^ mice 20 days after MI or Sham surgery. *n* = 4 for each group, and ~100–150 positive cells were counted. (**E**) TUNEL assay for heart tissue sections (border zone) of WT and *Fam114a1*^–/–^ mice 20 days after surgery. Scale bar: 20 μm. *n* = 5 for each group. (**F**) Representative echocardiographic images of WT and *Fam114a1*^–/–^ mice at baseline and 2 weeks after surgery. *n* = 8 for each group. Quantification of EF and FS was shown. Data are presented as mean ± SEM. Statistical significance was confirmed by 2-way ANOVA with Tukey’s multiple-comparison tests for **A–F**. *P < 0.05; **P < 0.01; ***P <0.001;.****P <0.0001.

To further confirm the phenotype of *Fam114a1*^–/–^ mice under Ang II infusion, we subjected *Fam114a1*^–/–^ mice to a more physiologically relevant HF model of permanent left anterior descending (LAD) coronary artery ligation-mediated MI. Ischemia-induced cardiac hypertrophy was significantly blunted in *Fam114a1*^–/–^ mice 20 days after MI (Figure 3, A and B). We next assessed the effects of Fam114a1 deficiency on the ischemic myocardium by staining for 2, 3, 5-triphenyltetrazolium chloride (TTC). The infarct areas were markedly reduced in *Fam114a1*^–/–^ mice (Supplemental Figure 3A). The fibrosis areas were also significantly reduced in *Fam114a1*^–/–^ mice (Figure 3C and Supplemental Figure 3B). Consistently, fibrosis marker gene expression was decreased in *Fam114a1*^–/–^ mice, including *Col1a1* and *Col3a1* (Supplemental Figure 3C). During MI, CFs start proliferation while CMs undergo apoptosis and cell death upon the cardiac injury. Therefore, we examined the cellular phenotypes of CFs and CMs. We observed less CF cell proliferation (Figure 3D and Supplemental Figure 3D) and CM apoptosis (Figure 3E and Supplemental Figure 3E) in *Fam114a1-*KO mice than in WT mice after MI surgery, using PDGFRα and BrdU staining and TUNEL assay, respectively. To further explore whether the reduced CM death is due to an autonomous effect, we isolated primary adult CMs from WT and *Fam114a1*-KO mice at baseline and stressed the cells with Ang II for 48 hours. We observed that KO CMs showed significantly reduced apoptosis and compromised hypertrophy compared with the WT CMs (Supplemental Figure 3, F and G). Of note, the cardiac function in *Fam114a1*^–/–^ mice was significantly improved compared with WT mice 14 days after MI surgery, indicated by partially recovered EF and FS and reduced left ventricle volumes (Figure 3F, Supplemental Figure 3H, and Supplemental Table 3). Taken together, these results suggest that *Fam114a1* loss in CFs and CMs synergistically contributes to the cardioprotective outcome.

To test whether prolonged MI in *Fam114a1*^–/–^ mice causes accelerated decompensation and cardiac rupture, we subjected *Fam114a1*^–/–^ mice to a LAD ligation surgery and monitored cardiac function for 8 weeks after MI, and we examined the morphological and histological changes in the hearts. We did not observe any cardiac ruptures in the *Fam114a1*^–/–^ mice 8 weeks after MI surgery (Supplemental Figure 4A). Echocardiographic examinations during 8 weeks after MI surgery revealed consistent protection against MI triggered cardiac damage, as indicated by improved EF and FS and reduced left ventricular chamber size at systolic and diastolic phases, without any significant change in heart rates (Supplemental Figure 4, B–E, and Supplemental Table 4). Picrosirius red staining results confirmed reduced cardiac fibrosis in KO hearts compared with WT hearts (Supplemental Figure 4F). In summary, *Fam114a1*^–/–^ mice exhibited reduced cardiac fibrosis and myocyte hypertrophy, enhanced myocyte survival, and improved cardiac function in HF models under neurohumoral and ischemic stress conditions.

### Fam114a1 deficiency in mice reduces cardiac inflammation and oxidative stress under pathogenic stresses.

**Figure 4:**
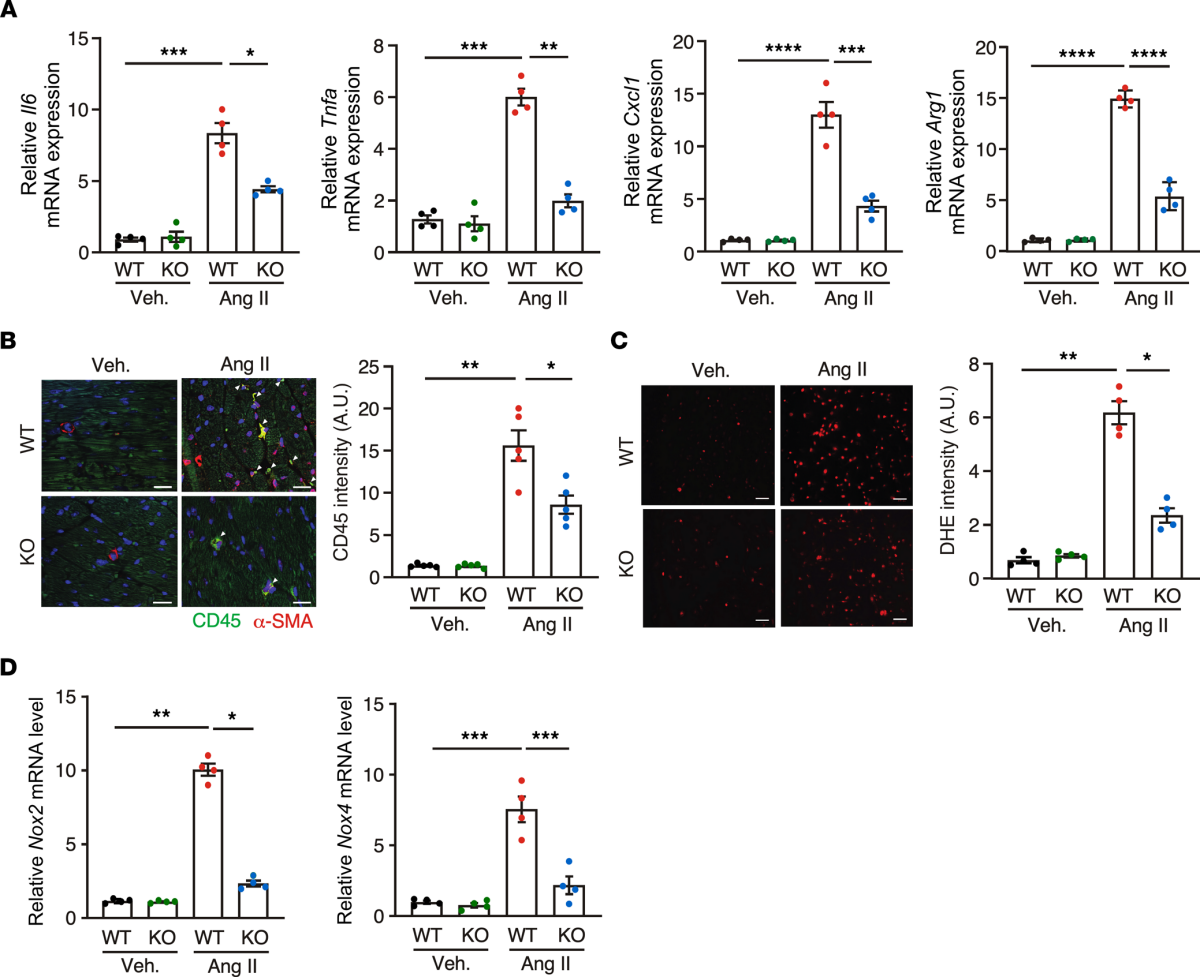
*FAM114A1* deletion antagonizes Ang II–induced inflammation and oxidative stress. (**A**) qPCR measurement of mRNA expression levels of *Il6*, *Tnfa*, Cxcl1, and *Arg1* in WT and *Fam114a1*^–/–^ mice. *n* = 4 for each group. *Gapdh* mRNA was used as normalizer. (**B**) Representative IF images show CD45 (green) and α-SMA (red) in heart tissue sections of WT and *Fam114a1*^–/–^ mice after 4 weeks of Ang II infusion. Scale bar: 20 μm. *n* = 5 for each group. (**C**) DHE staining of cultured CF cells isolated from WT and *Fam114a1*^–/–^ mice. Scale bar: 100 μm. *n* = 4 for each group. (**D**) qPCR measurement of mRNA expression level of NADPH oxidase genes. *Gapdh* mRNA was used as a normalizer. *n* = 4 for each group. Data are presented as mean ± SEM. Statistical significance was confirmed by 2-way ANOVA with Tukey’s multiple-comparison tests for **A**–**D**. *P < 0.05; **P < 0.01; ***P <0.001;.****P <0.0001.

One major downstream effect of Ang II stimulation and MI is increased inflammatory responses and consequent injury to the heart. Therefore, we examined whether FAM114A1 deletion influences inflammation in Ang II–infused mouse hearts. We showed that the mRNA expression of inflammatory genes (such as *Il6*, *Tnfa*, *Cxcl1*, and *Arg1*) was reduced in *Fam114a1*^–/–^ hearts compared with WT hearts (Figure 4A), suggesting that *Fam114a1* deficiency inhibits local inflammatory responses in the Ang II–infused hearts. Cytokine array analyses in mouse serum samples showed that loss of FAM114A1 reduced circulating CXCL9 and CXCL10 levels but not any other cytokines or chemokines (Supplemental Figure 5A), indicating modest alterations in systemic inflammation. Also, we observed less infiltration of CD45^+^ leukocyte cells in the myocardium of *Fam114a1*^–/–^ mice than WT mice upon Ang II infusion, suggesting reduced inflammatory responses in the KO hearts (Figure 4B). Likewise, less immune cell infiltration was observed in both infarct and remote areas of MI hearts of *Fam114a1*^–/–^ mice compared with WT mice, while no difference was seen under sham conditions between the WT and *Fam114a1*^–/–^ mice (Supplemental Figure 5, B and C). Reactive oxygen species (ROS) are primary inflammatory response triggers in the heart after cardiac injury. Thus, we next examined the ROS generation in cultured CF cells from WT and *Fam114a1*^–/–^ mice based on the high level of FAM114A1 expression in CFs. Dihydroergotamine (DHE) staining showed that ROS production was drastically induced in cultured WT CFs under Ang II treatment and significantly reduced in *Fam114a1*^–/–^ CFs (Figure 4C). One primary source of ROS is produced by nicotinamide adenine dinucleotide phosphate (NADPH) oxidases such as NOX2 and NOX4. We measured the expression of NADPH oxidases and found that *Nox2* and *Nox4* mRNA levels were significantly reduced in *Fam114a1*^–/–^ hearts compared with WT hearts after Ang II infusion (Figure 4D). Altogether, these results suggest that deletion of *Fam114a1* ameliorates Ang II–induced cardiac inflammation and oxidative stress in the genetic animal model.

### Loss of FAM114A1 in CFs attenuates the response to Ang II signaling transduction and inhibits fibroblast proliferation and activation.

**Figure 5:**
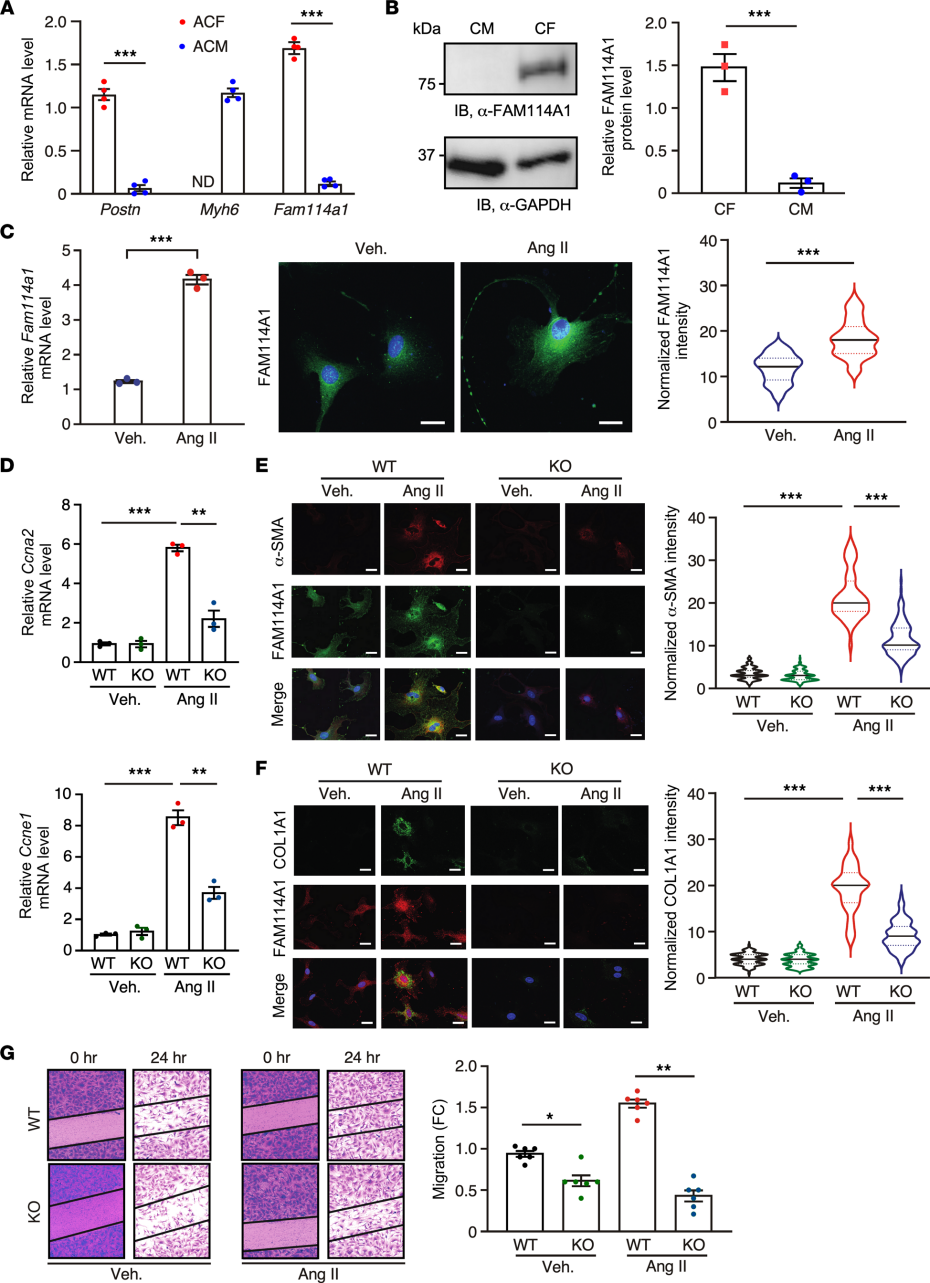
*FAM114A1* deletion abolishes Ang II–induced cardiac fibroblast proliferation, myofibroblast activation, and migration. (**A**) *Fam114a1* mRNA expression in PMCFs and CMs. *Gapdh* mRNA was used as normalizer. *n* = 4 for each group. (**B**) Endogenous FAM114A1 protein expression in PMCFs and CMs detected by Western blot. GAPDH protein was used as a loading control. *n* = 3 for each group. (**C**) Ang II treatment (1 μM) induces *FAM114A1* mRNA and protein expression in PMCFs. *n* = 3 for qPCR and *n* = 100–120 cells per treatment for IF. 18S rRNA was used as normalizer for mRNA measurement. Scale bar: 20 μm. (**D**) qPCR measurement of mitotic cyclin gene expression in CFs from WT and *Fam114a1*^–/–^ mice after Ang II treatment. *n* = 3 for each group. 18S rRNA was used as a normalizer. (**E** and **F**) Representative images of IF staining and quantification of normalized intensity of MF activation markers in CFs of WT and *Fam114a1*^–/–^ mice after vehicle or Ang II treatment. *n* = 150–200 cells were analyzed for each group. Scale bar: 20 μm. (**G**) Representative images of migrating CFs from WT and *Fam114a1*^–/–^ mice after vehicle or Ang II treatment. Quantification of scratch closure after 24 hours was shown. *n* = 6 biological replicates for each group. Data are presented as mean ± SEM. Statistical significance was confirmed by 2-tailed Student *t* test for **A**–**C** and 2-way ANOVA with Tukey’s multiple-comparison test for **D**–**G**. *P < 0.05; **P < 0.01; ***P <0.001.

To determine the function of FAM114A1 in specific cardiac cell types, we first surveyed the GENEVESTIGATOR database (https://genevestigator.com ). We found that *FAM114A1* mRNA was dominantly expressed in multiple human fibroblast cells from different organs (e.g., skin, liver, heart) and modestly expressed in immune cell types (e.g., T cells, monocytes) (Supplemental Figure 6A, left panel). We also examined the single-cell RNA-Seq data of the human heart from the Human Protein Atlas database (https://www.proteinatlas.org) and confirmed that *FAM114A1* mRNA was highly expressed in CFs but lowly expressed in CMs (Supplemental Figure 6A, right panel). We further demonstrated that Fam114a1 mRNA and protein were dominantly expressed in isolated PMCFs and barely detected in PMCMs (Figure 5, A and B). This observation indicates that FAM114A1 may primarily regulate gene expression and cellular function in CFs. Thus, we isolated PMCFs for in vitro Ang II treatment. The purity of cultured CFs isolated from WT and KO mice was confirmed by immunostaining with cell type–specific markers, including vimentin (CF), CD31 (endothelial cell), and CD45 (all hematopoietic cells) (Supplemental Figure 6B). Ang II significantly induced Fam114a1 mRNA and protein expression in cultured mouse CFs (Figure 5C). Ang II infusion induced a CF gene signature of accelerated cell proliferation and cell cycle progression (Figure 5D). Expression of CF proliferation and promitotic cell cycle marker genes *Ccna2* and *Ccne1* and MF activation marker genes *Acta2* and *Col1a1* were significantly reduced in the CFs from *Fam114a1*^–/–^ hearts compared with those of WT hearts upon Ang II treatment (Supplemental Figure 6C). Consequently, α-SMA and COL1A1 protein expression was markedly reduced in the CFs from *Fam114a1*^–/–^ hearts than in WT hearts after Ang II treatment (Figure 5, E and F). Moreover, the cell migration ability of *Fam114a1*^–/–^ CFs was weaker than that of WT CFs at baseline and upon Ang II treatment (Figure 5G). Taken together, our results indicate that FAM114A1 is dominantly expressed in CFs, inducible upon Ang II stimulation, and is required for efficient CF cell proliferation, activation, and migration.

### FAM114A1 interacts with AGTRAP and regulates AGTRAP and AT1R expression in CFs.

**Figure 6:**
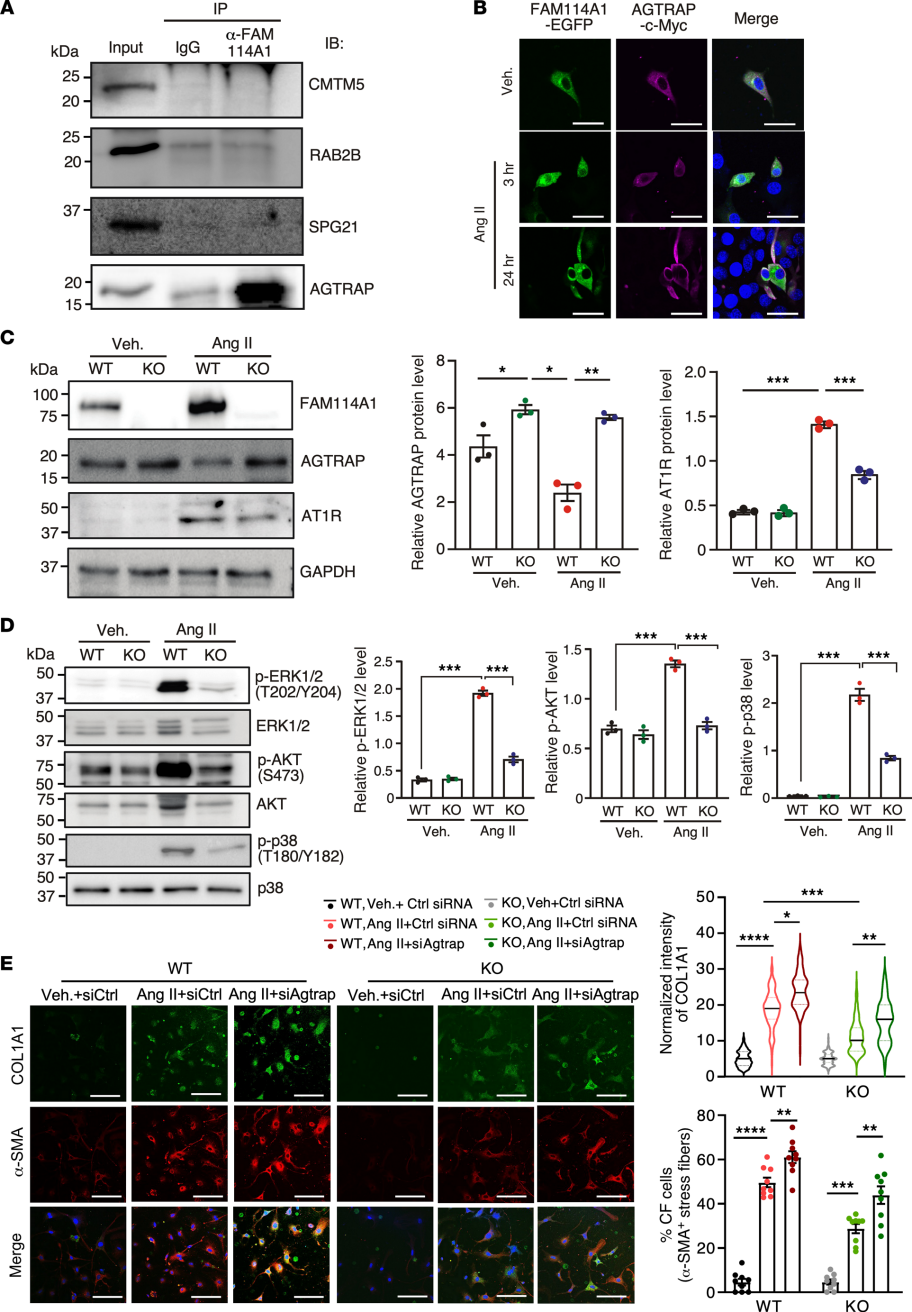
FAM114A1 depletion stabilizes AGTRAP, enhances AT1R degradation, and influences Ang II signaling in cardiac fibroblasts. (**A**) Immunoprecipitation and immunoblot confirm the interaction of FAM114A1 with AGTRAP in the mouse heart tissue lysates. This experiment was repeated 3 times, and representative images are shown. (**B**) IF detection of overexpressed EGFP-tagged FAM114A1 and Myc-tagged AGTRAP in mouse NIH/3T3 fibroblast cells. Protein expression changes were observed after 3 hours and 24 hours after Ang II or vehicle treatment. Scale bar: 50 μm. This experiment was repeated 3 times, and representative images are shown. (**C**) Western blot measurement and quantification of FAM114A1, AGTRAP, and AT1R in vehicle or Ang II–treated primary CF cells isolated from WT and *Fam114a1*^–/–^ mice. Separate gels were run for detecting FAM114A1 and AGTRAP compared with AT1R and GAPDH. (**D**) Western blot measurement and quantification of p-ERK1/2, ERK1/2, p-AKT, AKT, p-p38, and p38 in Ang II– or vehicle-treated CFs (24 hours) from hearts of WT and *Fam114a1*^–/–^ mice (*n* = 3). (**E**) Representative images of IF staining and quantification of normalized intensity of MF activation markers in CFs of WT and *Fam114a1*^–/–^ mice with control or Agtrap siRNA transfection followed by vehicle or Ang II (1 μM) treatment. *n* = 100 cells were analyzed for each group. Scale bar: 30 μm. Data are presented as mean ± SEM. Statistical significance was confirmed by 2-way ANOVA with Tukey’s multiple-comparison test for **C**–**E**. *P < 0.05; **P < 0.01; ***P <0.001;.****P <0.0001.

To determine the molecular function of FAM114A1 in CF cells, we first searched for its proteomic interactome. Publicly available protein-to-protein interaction data have uncovered several potential interacting partners of FAM114A1, including CMTM5, RAB2B, SPG21, and AGTRAP, among others (Supplemental Figure 7A) (25). The yeast 2-hybrid screen discovered these candidate interacting proteins, suggesting a direct physical interaction between the candidate protein and FAM114A1. Out of 4 tested candidate proteins, we validated the interaction between AGTRAP and FAM114A1 in mouse heart lysates and cultured PMCFs by IP and Western blotting (Figure 6A and Supplemental Figure 7B). FLAG-tagged FAM114A1 recombinant protein was also associated with AGTRAP when overexpressed in NIH/3T3 fibroblast cells (Supplemental Figure 7C). AGTRAP has been reported to bind AT1R and promote its internalization and degradation (22–24). Therefore, we sought to examine the role of FAM114A1 in the regulation of the Ang II signaling via interacting with AGTRAP. At baseline, EGFP-tagged FAM114A1 was partially colocalized with Myc-tagged AGTRAP in vehicle-treated fibroblast cells (Figure 6B). Upon Ang II stimulation of fibroblast cells, the expression level of EGFP-tagged FAM114A1 was enhanced. In contrast, Myc-tagged AGTRAP level was decreased in the cytoplasm (Figure 6B). Using PMCF cell culture, we found that AGTRAP protein expression (but not mRNA) was increased in Ang II–treated *Fam114a1*^–/–^ CFs compared with WT CFs (Figure 6C and Supplemental Figure 7D). In contrast, AT1R protein expression was decreased in Ang II–treated *Fam114a1*^–/–^ CFs (Figure 6C). Moreover, we observed reduced AGTRAP and increased AT1R together with induced FAM114A1 protein expression in failing human hearts compared with nonfailure donor hearts (Supplemental Figure 7E). These observations in human samples are consistent with the data obtained in Ang II–treated primary CFs of WT and *Fam114a1*^–/–^ mice (Figure 6C). Collectively, these results suggest that FAM114A1 interacts with AGTRAP in CFs and that loss of FAM114A1 increases AGTRAP protein expression and may promote AT1R protein turnover.

Since FAM114A1 regulates protein expression of AGTRAP and AT1R, we next examined the role of FAM114A1 in Ang II–induced signaling transduction in cultured primary CFs from WT and *Fam114a1*^–/–^ mice. Activation of ERK1/2, AKT, and p38 is a hallmark of Ang II signaling transduction in cardiac cells. Therefore, we measured the phosphorylation of these kinases, and our immunoblot results reveal that, in Ang II–treated *Fam114a1*^–/–^ CFs, phosphorylation of ERK1/2, AKT, and p38 was significantly reduced compared with WT CFs (Figure 6D). These data suggest that the deletion of *Fam114a1* antagonizes the Ang II–induced activation of downstream effectors and reduces collagen deposition.

To examine whether the interaction between FAM114A1 and AGTRAP is causal for the fibrotic phenotype, we knocked down *Agtrap* using specific siRNA in *Fam114a1*-KO PMCFs (Supplemental Figure 7F). We found that MF marker protein expression (COL1A1) and α-SMA^+^ stress fiber containing CF cells were significantly increased in the *Fam114a1-*KO (in light green) and WT PMCFs after Ang II treatment (Figure 6E). After siRNA knockdown of *Agtrap*, α-SMA^+^ stress fiber containing CF cells were increased in population (in dark green and dark red). Cell proliferation was increased to the level of WT PMCFs (Supplemental Figure 7G). In addition, we overexpressed FAM114A1 and AGTRAP by cotransfection. We found that, in Ang II–treated PMCFs, the α-SMA^+^ stress fiber containing MF cells and COL1A1 expression were increased by FAM114A1 overexpression alone but restored to the level of control lentivirus-treated group by cooverexpression of AGTRAP (Supplemental Figure 7H). Furthermore, we found that PMCF cell proliferation was enhanced by FAM114A1 overexpression and reduced to the level of control lentivirus-treated group by coexpression of AGTRAP (Supplemental Figure 7I). These data suggest that the FAM114A1/AGTRAP interaction is required for enhanced CF proliferation and activation under Ang II stimulation.

### Transcriptome profiling reveals dysregulated gene pathways in Fam114a1-null CFs.

**Figure 7:**
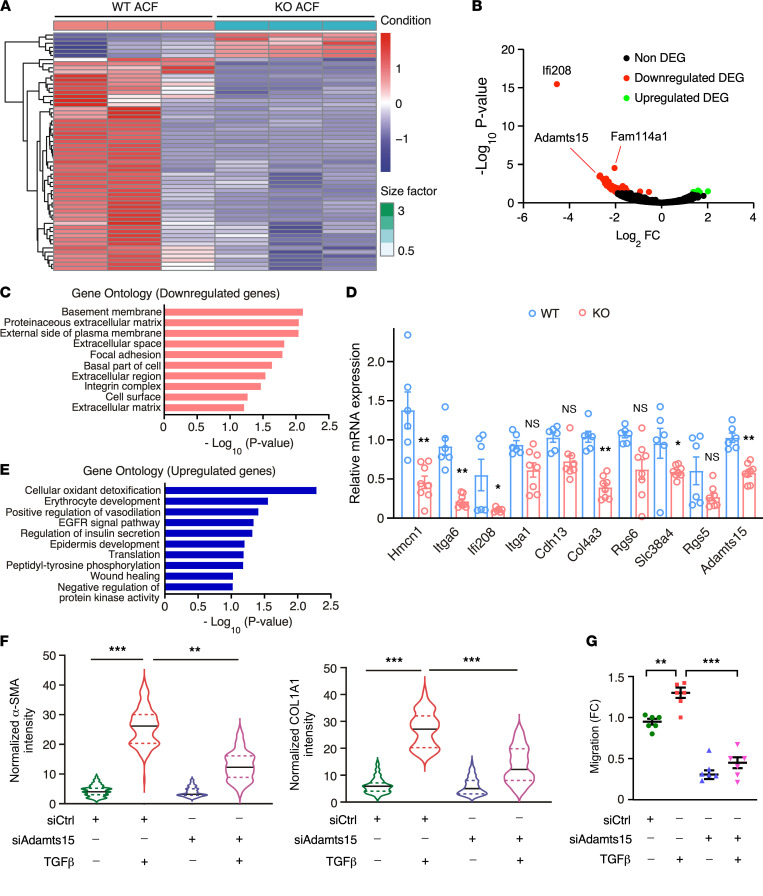
*ADAMTS15*, a differentially regulated gene in *Fam114a1*-null cardiac fibroblasts, is required for CF-to-MF activation. (**A**) Heatmap of gene expression in CFs isolated from WT and *Fam114a1*^–/–^ mice at baseline analyzed by RNA-Seq. P60 male mice, *n* = 3 per group, *P*_adj_ < 0.05. (**B**) Volcano plot of differentially expressed genes in CFs isolated from WT and *Fam114a1*^–/–^ mice at baseline analyzed by RNA-Seq. (**C**) Gene Ontology analysis of enriched pathways of downregulated genes in RNA-Seq. The top pathways are listed with enriched gene sets (cellular component). (**D**) qPCR validation of multiple downregulated genes in *Fam114a1*^–/–^ mice derived CFs. 18S rRNA was used as a normalizer (*n* = 3). (**E**) Gene Ontology analysis of enriched pathways of upregulated genes in RNA-Seq (biological process). (**F**) IF quantification of COL1A1 and α-SMA protein expression after knockdown of *Adamts15* in TGF-β–treated PMCFs. *n* = 100–120 cells from 3 biological replicates were analyzed. (**G**) Knockdown of *Adamts15* reduced PMCF cell migration indicated by scratch assays (*n* = 6). Data are presented as mean ± SEM. Statistical significance was confirmed by unpaired Student *t* test for **D** and 1-way ANOVA with Tukey’s multiple-comparison test for **F** and **G**. *P < 0.05; **P < 0.01; ***P <0.001.

To further understand global gene expression changes in *Fam114a1*-null CF cells and evaluate the safety of complete inactivation of FAM114A1, we performed RNA-Seq in isolated primary adult CFs from WT and *Fam114a1*^–/–^ mice. Multiple dysregulated genes were uncovered at the steady-state mRNA level in *Fam114a1*^–/–^ CFs (Figure 7, A and B). *Fam114a1* mRNA was reduced 4-fold, probably due to a robust nonsense-mediated mRNA decay triggered by the presence of a premature termination codon introduced by CRISPR-Cas9–mediated gene editing (Supplemental Figure 2A). Several genes showed a more drastic reduction of mRNA expression compared with *Fam114a1*, such as *Ifi208*, *Adamts15*, and *Serpinc1*, among others (Supplemental Table 5). DAVID gene ontology analyses suggest that the downregulated genes are mainly involved in 2 aspects, including basement membrane and ECM (Figure 7C). The decrease of a select of genes was confirmed by qPCR (Figure 7D). On the other hand, we also observed increased gene expression in pathways of cellular oxidant detoxification and positive regulation of vasodilation (Figure 7E). These transcriptomic profiling data suggest that deletion of FAM114A1 in CFs reduces the expression of a small cohort of ECM genes, among others, without triggering dramatic global gene expression changes at baseline.

### FAM114A1 downstream effector ADAMTS15 is required for CF activation and migration.

Findings of FAM114A1-mediated regulation of CF activation prompted us to identify further critical downstream effectors involved in the pathological process of FAM114A1-driven cardiac fibrosis. Among the validated downregulated genes in *Fam114a1*-null CFs, *Adamts15* is among the most downregulated genes with high baseline mRNA expression. Furthermore, it has been identified as a potential modulator of hepatic fibrosis (40). Therefore, we sought to test whether ADAMTS15 plays a role in CF activation during cardiac fibrosis. We found that *ADAMTS15* mRNA expression was increased in failing human hearts compared with nonfailure donor hearts (Supplemental Figure 8A) and positively correlated with *ACTA2* mRNA expression (Supplemental Figure 8B). ADAMTS15 protein was colocalized with α-SMA^+^ CF cells in failing human hearts (Supplemental Figure 8C) and mouse MI hearts (Supplemental Figure 8D). siRNA-mediated knockdown of *Adamts15* in mouse primary CFs significantly reduced protein expression of α-SMA and COL1A1 upon TGF-β treatment (Figure 7F and Supplemental Figure 8E). In addition, ADAMTS15 knockdown significantly inhibited CF cell migration at baseline and after TGF-β stimulation (Figure 7G). Taken together, we discovered that FAM114A1 depletion in CFs reduces ECM gene expression at baseline, and 1 critical downstream effector gene, *ADAMTS15*, is required for TGF-β–triggered CF activation and migration.

## Discussion

This study found that a potentially novel MI- and CAD-associated gene, FAM114A1, plays an important role in pathological cardiac remodeling and fibrosis. FAM114A1 is induced in failing human hearts and HF mouse models of Ang II infusion and MI surgery. We observed high expression of FAM114A1 in CFs but low expression in CMs and immune cells. We used a global *Fam114a1*-KO mouse model to demonstrate that deletion of *FAM114A1* antagonizes pathological cardiac remodeling, including cardiac fibrosis, CM hypertrophy, and inflammation in vivo using Ang II and MI models. We have identified AGTRAP as a direct interacting partner of FAM114A1 in CFs. Cardiac stress–induced FAM114A1 downregulates the expression of AGTRAP and increases AT1R level, thereby enhancing Ang II signaling. Based on transcriptome profiling in *Fam114a1*-null CFs, we identified multiple dysregulated genes involved in cardiac fibrosis, including a critical ECM protein ADAMTS15 required for TGF-β–triggered CF activation and migration. Our work indicates the importance of FAM114A1 in the pathogenesis of cardiac remodeling and establishes FAM114A1 as a potentially novel therapeutic target for heart disease with high safety upon complete inactivation.

To date, multiple signaling pathways have been reported to induce cardiac fibrosis, including TGF-β (15), ERK (16), SMAD2/3 (41), p38 MAPK (42), and EPRS (43). Since all these pathways are downstream of Ang II/AT1R signaling transduction (44), therapeutic strategies targeting molecules associated with Ang II signal transduction are promising to treat cardiac fibrosis and prevent HF. Our studies have 2 conceptual advances in treating cardiac remodeling by abolishing FAM114A1-mediated amplification of Ang II signaling.

First, this work is a pioneering study of the FAM114A1 and FAM114A1/AGTRAP axis that modulates cardiac remodeling. We demonstrate that FAM114A1 is a potentially novel regulatory factor of angiotensin signaling through modulating AGTRAP protein expression in CFs. Multiple pathological cardiac stresses induce FAM114A1 such as Ang II stimulus and ischemia. Under cardiac stresses, increased FAM114A1 may promote cardiac fibrosis and remodeling through the FAM114A1/AGTRAP/AT1R axis in CFs. MG132 inhibition of proteasomal degradation pathway stabilized AGTRAP in PMCFs and reduced expression of MF marker protein Periostin under Ang II treatment (Supplemental Figure 7J), while siRNA knockdown of *Agtrap* in *Fam114a1*-KO mouse CFs restored MF marker protein expression (Figure 6E), suggesting that FAM114A1 regulates AGTRAP expression at the protein but not mRNA level (Supplemental Figure 7K). We assume that FAM114A1 may act as an adaptor to escort AGTRAP for proteasomal degradation. We knocked down a related immunoproteasome catalytic subunit β5i-encoding gene *Psmb8* (45) but found no change in AGTRAP expression in *Fam114a1*-KO PMCFs (not shown). Thus, unbiased screening of E3 ubiquitin ligases is required to identify factors for FAM114A1-mediated regulation of AGTRAP protein stability, and the detailed mechanism needs to be elucidated in the future.

Second, we provide evidence to support the antiremodeling and antifibrosis effects of genetic inactivation of FAM114A1. Global *Fam114a1*-KO animals do not exhibit noticeable pathological changes at baseline. A prolonged period of MI insult for 8 weeks did not trigger any cardiac ruptures in *Fam114a1*-KO mice (Supplemental Figure 4, A–F). Also, we did not find any accelerated decompensation, and the cardiac function was still improved. We assume that inactivation of FAM114A1 as a positive regulator of Ang II signaling may not abolish the reparative fibrosis in the acute phase after MI and, instead, can weaken the persistent pathological fibrosis in the chronic phase. This indicates that FAM114A1 is a potential drug target for antifibrosis treatment without triggering deleterious side effects when inhibited or depleted. Using transcriptome-wide profiling of isolated *Fam114a1-*null CF cells, we discovered that FAM114A1 inactivation led to reduced expression of a selective cohort of ECM genes without causing global changes in gene expression (Figure 7). One downstream effector of FAM114A1, ADAMTS15, plays a vital role in regulating CF activation and migration. We also observed increased gene expression of cellular oxidant detoxification and positive regulation of vasodilation, suggesting a compensatory response that may facilitate the relief of oxidative stress and pressure overload during cardiac pathogenesis when the heart is stressed. Given the notion of Ang II activation of the canonical TGF-β signaling pathway (15, 46), we examined the response of *Fam114a1*-KO CFs after TGF-β stimulation. We found that *Fam114a1*-KO reduced CF activation (Supplemental Figure 9A). This observation suggests that FAM114A1 may influence TGF-β signaling at a node merging with a downstream signaling effector of the Ang II/AT1R pathway such as p38 MAP kinase (Figure 6D) (47).

### Limitation.

*FAM114A1* has multiple SNPs associated with human inflammatory diseases such as ankylosing spondylitis and host immune response to bacterial infection (48, 49). Importantly, human GWAS databases (GWASATLAS; https://atlas.ctglab.nl) demonstrate that *FAM114A1* has a genetic SNP mutation related to hypertension phenotype in human CAD (ranked as 89 of 19,057 genes; *P* = 3.64 × 10^–9^) (Supplemental Figure 1A) (26). In another independent study, one *FAM114A1* SNP (rs1873197; *P* = 3.32 × 10^–6^) was identified as a CAD-associated locus with CAD diagnosis (e.g., MI, acute coronary syndrome, chronic stable angina, or coronary stenosis > 50%) (27). The genetic association of the same *FAM114A1* SNP (rs1873197) with MI was further confirmed by a recent large-scale genome-wide analysis (*n* = 639,000 human MI participants; effect allele frequency is 6.7743%; *P* = 8.48 × 10^–5^) (Supplemental Figure 1B) (28). These GWAS data suggest a potential functional connection of *FAM114A1* with immune or vascular cell types. The Ang II infusion model involves multiple cardiac cell types in addition to CMs and CFs such as endothelial cells and SMCs because Ang II signaling acts on both vascular and cardiac cells. Consistent with the feature of this model, we observed mildly reduced systolic blood pressure in *Fam114a1*-KO mice compared with WT mice after Ang II infusion for 4 weeks (Supplemental Figure 9B). This antihypertensive effect from *Fam114a1* loss may contribute to the reduction of cardiac hypertrophy. To exclude the compounded effects from vascular cells and systemic blood pressure changes, we used isolated mouse primary CFs from *Fam114a1*-KO mice to prove cell type–specific autonomous effects in CFs (Figure 5). To further support this idea, we used the MI model, which is less dependent on SMCs than the Ang II model. We also demonstrated similar antifibrotic effects of deletion of *Fam114a1* in MI mice. FAM114A1 is modestly expressed in immune cells, including monocyte, T cells and B cells (Supplemental Figure 5A). However, some effects of FAM114A1 loss of function in immune cells may contribute to reduced cardiac inflammation and compromised fibrotic response. Interestingly, we also observed reduced CM death in *Fam114a1*-KO hearts under cardiac stresses (Figure 3E and Supplemental Figure 3, E and F), suggesting that enhanced CM survival contributes to the cardiac protective phenotypes in *Fam114a1*-null mice. This may be driven by either CM-specific autonomous effect despite low expression in CMs or a nonautonomous effect via CF-CM intercellular communication (50). The function of FAM114A1 in vascular cells, immune cells, and CMs warrants further studies to understand its biological role fully.

## Methods

Supplemental Methods are available online with this article.

### Human specimens.

All human samples of frozen cardiac tissues — including 20 samples from explanted failing hearts (10 ischemic HF [ISHF] and 10 dilated cardiomyopathy hearts) and 8 samples from nonfailing donor hearts, as well as paraffin section slides from dilated cardiomyopathy (DCM; *n* = 5), ISHF (*n* = 5), or nonfailing hearts (*n* = 7) — were acquired from the Cleveland Clinic. In Figure 1A, total RNA samples from 2 failing hearts showed degradation during the quality control process and were excluded from qPCR analysis. All human samples were picked randomly based on the presence or absence of HF. We are blinded from any clinical data. The identity numbers of the frozen cardiac tissues and histology tissues are listed in Supplemental Methods. The experiments conformed to the principles outlined in the Declaration of Helsinki.

### Mice.

The global *Fam114a1*-KO mice were created by CRISPR technology in International Mouse Phenotyping Consortium. The alteration caused the deletion of a 314 bp nucleotide sequence, which results in the deletion of exon 3, amino acid sequence change after the residue 116, and early termination 3 amino acids later. For experiments with *Fam114a1*^–/–^ mice, control mice of the same age and sex from littermates or sibling mating were used. All animal procedures were performed in accordance with the NIH and the University of Rochester Institutional guidelines. Two different disease models of HF were used: (a) Ang II infusion model (1.4 μg/g/day) and (b) permanent LAD ligation model (MI). The mice were randomized for experiments using simple randomization with a specific ID number before animal procedures. Ang II and MI models, as well as sections and histology analyses, were performed in a blinded fashion.

### Data availability.

RNA-Seq data produced in this study were deposited under accession no. GSE165074 in Gene Expression Omnibus database.

### Statistics.

All quantitative Data are presented as mean ± SEM and analyzed using the GraphPad Prism (9.0.0) software. For normally distributed data, an unpaired 2-tailed Student *t* test was performed to compare 2 groups and 1-way or 2-way ANOVA with Tukey’s multiple-comparison test for the comparisons between more than 3 groups. For data that are not normally distributed, a nonparametric Mann-Whitney *U* test was performed for the comparisons between 2 groups and Kruskal-Wallis test with Dunn’s multiple-comparison test for the comparisons between more than 3 groups. Statistical significance was assumed at a value of *P* < 0.05.

### Study approval.

The experiments of gene expression measurement in human tissue samples were approved by Material Transfer Agreement between the University of Rochester Medical Center and the Cleveland Clinic and were conformed to the principles outlined in the Declaration of Helsinki. The animal studies have been approved by the University of Rochester Medical Center Animal Care. All animal procedures were performed in accordance with the NIH and the University of Rochester Institutional guidelines.

## Author contributions

PY conceived the study, provided the funding, and wrote the manuscript. KCVS and PY designed the experiments and analyzed the data. KCVS carried out all the experimental work and contributed to the writing of the manuscript. JW contributed to technical support, conceptual ideas, and manuscript editing. WHWT provided critical human samples for our studies. All the authors discussed the results and had the opportunity to comment on the manuscript.

## Supplementary Material

Supplemental data

## Version 1 06/07/2022

In-Press Preview

## Version 2 07/08/2022

Electronic publication
