# Supplementary material for: FAM114A1 influences cardiac pathological remodeling by regulating angiotensin II signaling
Source: JCI Insight. 2022 Jul 8;7(13):e152783. doi: 10.1172/jci.insight.152783 (PMC9310534; doi:10.1172/jci.insight.152783)

## Supplementary Information for

# FAM114A1 Influences Cardiac Pathological Remodeling by Regulating Angiotensin II Signaling

Kadiam C Venkata Subbaiah, Jiangbin Wu, Wai Hong Wilson Tang, Peng Yao*

Correspondence to Peng Yao

E-mail: peng_yao@urmc.rochester.edu

**This document file includes:**

Figures S1 to S9

Tables S1 to S5

SI Materials and Methods

SI References

## Supplemental figures

**Figure S1. Genome-wide association of *FAM114A1* SNP with human heart disease and increased FAM114A1 expression in human and mouse heart failure and correlation with collagen expression.**

(**A**) Manhattan plot of human genome-wide association study containing *FAM114A1* SNP rs1873197 associated with coronary artery disease (from GWASATLAS database).

(**B**) *FAM114A1* SNP rs1873197 as a human genomic locus associated with myocardial infarction (n=639,000 human MI subjects and n=192,000 human coronary atherosclerosis subjects).

(**C**) IF analysis and quantification of FAM114A1 protein expression in failing human hearts. $\alpha$-actinin IF indicates FAM114A1 protein expression in non-myocyte cells such as CFs (100-130 cells were quantified). Scale bar: 20 $\mu$m.

(**D**) Picrosirius red staining of failing human hearts versus non-failure donor hearts (n=7 for NF; n=10 for HF). Scale bar: 0.1 mm.

(**E**) FAM114A1 expression is correlated with the expression of collagens (not ANP or BNP) in human heart samples (n=26; 8 for NF and 18 for HF). Pearson correlation coefficient was presented. 18S rRNA was used as a normalizer.

(**F**) *FAM114A1* mRNA expression is increased in idiopathic dilated cardiomyopathy (IDCM, n=15) and ischemic cardiomyopathy (ICM, n=11) patients compared to non-failure (NF, n=11) heart tissues from a public microarray dataset GDS651 / 213455_at.

(**G**) IF analysis and quantification of FAM114A1 protein expression in mouse MI-derived failing hearts. $\alpha$-actinin IF indicates FAM114A1 protein expression in non-CM cells such as CFs (n=4 per group).100-150 cells were counted for quantification. Scale bar: 10 $\mu$m.

(**H**) Tandem IF analysis of human heart failure and non-failing heart tissue sections using antibodies against FAM114A1, vimentin, and $\alpha$-SMA. Scale bar: 10 $\mu$m.

(**I**) Tandem IF analysis of mouse MI and sham heart tissue sections using antibodies against FAM114A1, Periostin, and $\alpha$-SMA. Scale bar: 10 $\mu$m.

(**J**) Western blot detection of FAM114A1 protein expression in isolated CF/MF from the WT Sham and MI (2 weeks post LAD ligation) mouse hearts (n=3).

(**K**) Venn diagram of candidate transcription factors involved in potential regulation of FAM114A1 expression.

(**L**) siRNA KD of *Nfatc3* reduces *Fam114a1* mRNA expression in PMCFs.

Data were represented as mean ± SEM. $^{**}P<0.01$; $^{***}P<0.001$; $^{****}P<0.0001$. Statistical significance was confirmed by unpaired two-tailed Mann Whitney test for C, unpaired two-tailed Student *t* test for F, G, J, L.

**Figure S2. Characterization of *Fam114a1*$^{-/-}$ mice at baseline.**

**(A)** Schematic of the generation of *Fam114a1*$^{-/-}$ mice by CRISPR technology from IMPC.

**(B)** Genotyping of WT (wild type), *Fam114a1*$^{+/-}$ (het: heterozygote)*, and Fam114a1*$^{-/-}$ (homo: homozygote) mice.

**(C)** RT-qPCR detection of *Fam114a1* mRNA using different primers spanning exon 1 and 2 (Set-1), exon 2-3 (Set-2), and exon 5-6 (Set-3). N=4 per group.

**(D)** Immunoblot shows FAM114A1 protein expression in the hearts of WT, *Fam114a1*$^{+/-}$*, and Fam114a1*$^{-/-}$ mice**.** Quantification of FAM114A1 protein intensity is shown. N=4 per group. The rabbit anti-FAM114A1 antibody (Cat. No. TA335404; Origene) was used in this experiment.

**(E)** Body size and heart morphology of WT and *Fam114a1*$^{-/-}$ mice at baseline. Scale bar: 1 mm.

**(F)** *Fam114a1*$^{-/-}$ mice show reduced HW/BW (heart weight/body weight) ratio after 4 weeks of Ang II infusion compared to WT mice. n=6 per group.

Data were represented as mean ± SEM. ***P*<0.01; ****P*<0.001. Statistical significance was confirmed by unpaired two-tailed Student *t* test for D and two-way ANOVA with Tukey's multiple comparisons test for F.

**Figure S3. Phenotypic characterization of *Fam114a1*$^{-/-}$ mice and CF and CM pathology after MI.**

**(A)** TTC (2,3,5-Triphenyltetrazolium chloride) staining of hearts of WT and *Fam114a1*$^{-/-}$ mice 20 days post-surgery (n=5 per group). The infarct area is calculated from the apex to the base of the heart sections. Scale bar: 1 mm.

**(B)** Representative images of Picrosirius red staining of cross sections of heart tissues of WT and *Fam114a1*$^{-/-}$ mice 20 days post-surgery. N=5 per group. Scale bar: 1 mm.

**(C)** RT-qPCR measurement of cardiac fibrosis marker gene expression in WT and *Fam114a1*$^{-/-}$ hearts after surgery. 18S rRNA was used as a normalizer. N=3 per group.
**(D)** CF cell proliferation was measured using BrdU/PDGFRα staining in the mouse heart tissue sections 20 days post-surgery (n=4). The percentage of PDGFα+ CFs in BrdU+ CFs. 100-150 cells were counted for the quantification in the border zone. Scale bar: 20 μm.

**(E)** Representative images of CM apoptosis in hearts of WT and *Fam114a1* KO mice subjected to the Sham or MI operation. Red color indicates TUNEL positive (apoptotic) nuclei; green indicates α-actinin (myocyte) staining; blue (DAPI) represents nuclei. TUNEL positive CMs were considered apoptotic cells for quantification. Scale bar: 10 μm.

**(F)** Trypan blue staining of isolated primary WT or *Fam114a1* KO mouse adult CMs treated with vehicle or Ang II (100 nM) for 48 hrs. Black color arrows indicate trypan blue positive myocytes. For the quantification 10 random fields and ~150-200 CMs were counted per dish. N=3 replicates for mice per group. Scale bar: 10 μm.

(**G**) Surface area quantification of primary WT and *Fam114a1* KO adult CMs treated with Ang II (200 nM) for 48 hrs. CMs were fixed with 4% PFA and stained with Alexa Fluor 594™ Phalloidin. N=50 CMs from each group were counted. Scale bar: 30 μm.

(**H**) Echocardiography measurement of end diastolic and systolic volume in WT and *Fam114a1*$^{-/-}$ mice after 2 weeks post-surgery. N=8 per group.

Data were represented as mean ± SEM. **P*<0.05; ***P*<0.01; ****P*<0.001; *****P*<0.0001. Statistical significance was confirmed by two-way ANOVA with Tukey's multiple comparisons test for A, C, E, G, H. Kruskal-Wallis test with Dunn's multiple comparisons test was performed for F.

**Figure S4. Phenotypic characterization of *Fam114a1*$^{-/-}$ mice after 8-week MI.**

**(A)** No cardiac rupture was found in *Fam114a1*$^{-/-}$ mice after 8-week MI (n=9 for WT and n=7 for KO, including male and female mice). Images of hearts harvested from WT and KO mice at 8 weeks post MI are shown.

(**B-E**) Heart rate (B), representative echocardiography images (C), EF, FS (D), LVESV, and LVEDV (E) of WT and *Fam114a1*$^{-/-}$ mice at baseline, 4 week, 6 week, and 8 week post-MI (n=9 for WT, n=7 for KO except week 4 (n=6 for KO due to technical difficulty in obtaining echo values).

(**F**) Representative images of Picrosirius red staining of cross sections of heart tissues of WT and *Fam114a1*$^{-/-}$ mice at 8 week post MI (n=7 for WT, n=6 for KO). The fibrosis area is calculated from the apex to the base of the heart sections. Scale bar: 1 mm.

Data were represented as mean ± SEM. ns: not significant; **P*<0.05; ***P*<0.01. Statistical significance was confirmed by two-way ANOVA with Tukey's multiple comparisons test for B, D, E, and F.

**Figure S5. Cytokine production and immune cell infiltration in *FAM114A1*$^{-/-}$ mice after MI.**

**(A)** Cytokine array analyses of serum samples of WT and *Fam114a1*$^{-/-}$ mice after vehicle or Ang II infusion. N=3 for each group. Three samples were pooled together for analysis.
**(B)** CD45 IF (red) and WGA (green) staining in WT and *Fam114a1*$^{-/-}$ mice after 20 days post Sham surgery. N=4 per group. Scale bar: 20 μm.
**(C)** CD45 IF (red) and WGA (green) staining in the remote and infarct area of WT and *Fam114a1*$^{-/-}$ hearts 20 days post MI surgery. N=4 per group. Scale bar: 20 μm.
Data were represented as mean ± SEM. ns: not significant; ***P*<0.01. Statistical significance was confirmed by unpaired two-tailed Student *t* test for B, C.

**Figure S6. FAM114A1 deletion reduces MF marker gene expression upon Ang II stimulation.**

**(A)** Left panel: The Genevestigator database provides expression ranking of FAM114A1 across multiple human cell types. Right panel: The Protein Atlas database provides expression ranking of *FAM114A1* mRNA across different cardiac cell types in the human heart.
**(B)** Cardiac fibroblast cells were isolated from WT mouse hearts (n=3) and cultured for 48 hrs. Cells were fixed with 4% PFA and stained with vimentin, CD31, and CD45 antibodies to check isolated CF cell purity.

**(C)** RT-qPCR measurement of *Fam114a1* and MF activation marker genes in Ang II or vehicle treated primary CFs isolated from WT and *Fam114a1*$^{-/-}$ mice. 18S rRNA was used as a normalizer (n=5).
Data were represented as mean ± SEM. ns: not significant; **P*<0.05; ***P*<0.01. Statistical significance was confirmed by two-way ANOVA with Tukey's multiple comparisons test for C.

**Figure S7. FAM114A1 interacts with AGTRAP and regulates CF proliferation and CF-to-MF activation.**

**(A)** Candidate interacting proteins of FAM114A1 from public databases (BioGRID and BioPlex). Proteins in bold were discovered by yeast two-hybrid genetic screen, while others were found by co-fractionation and mass spectrometry.
**(B)** IP and immunoblot confirm the interaction of FAM114A1 with AGTRAP using the AGTRAP antibody in isolated primary mouse CFs from WT mice. This experiment was repeated 3 times, and representative images are shown.
**(C)** IP and immunoblot confirm the interaction of FLAG-FAM114A1 with AGTRAP using FLAG antibody in mouse NIH/3T3 fibroblast cells. This experiment was repeated 3 times, and representative images are shown.
**(D)** *Agtrap* mRNA expression in the vehicle or Ang II treated (24 hrs) primary mouse CFs from WT and *Fam114a1*$^{-/-}$ mice (n=5 per group).
**(E)** Protein expression of endogenous FAM114A1, AGTRAP, and AT1R in failing human hearts compared to non-failure hearts by Western blot analysis.
**(F)** Knockdown efficiency of *Agtrap* siRNA in mouse CFs. *Gapdh* mRNA was used as a normalizer.
(**G, I**) CF proliferation assay using the MTT in *Fam114a1*$^{-/-}$ primary mouse CFs transfected with *Agtrap* siRNA (100 nM) and WT mouse CFs with co-overexpression of AGTRAP (1.5 μg DNA plasmids) and FAM114A1 (30 μL lentivirus) (n=3).
(**H**) Representative images of IF staining and quantification of normalized intensity of MF activation marker COL1A1 in PMCFs of WT and KO mice with control or Agtrap overexpression (1.5 μg DNA plasmid) or Fam114a1 lentivirus (30 μL) followed by vehicle or Ang II (1 μM) treatment. N=100 cells were analyzed per group. Percentage of CFs that are positive for α-SMA stress fibers was also quantified. Scale bar: 20 μm.
(**J**) Western blot analysis of protein expression levels of FAM114A1, AGTRAP, and Periostin in CFs from WT and KO mouse hearts (n=3) from (I). CFs were treated with Ang II (1 μM) and MG132 (5 μM) for 24 hrs.
(**K**) RT-qPCR analysis of mRNA expression levels of *Fam114a1* and *Agtrap* in CFs from WT and KO mouse hearts (n=3). CFs were treated with Ang II (1 μM) and MG132 (5 μM) for 24 hrs. The qPCR primers for *Fam114a1* mRNA are Set 2 (spanning exon 2-3).
Data were represented as mean ± SEM. ns: not significant; **P*<0.05; ***P*<0.01; ****P*<0.001; *****P*<0.0001. Statistical significance was confirmed by two-way ANOVA with Tukey's multiple comparisons test for D, J, K, Kruskal-Wallis test with Dunn's multiple comparisons test for H, and unpaired student *t* test for G, I.

**Figure S8. ADAMTS15 upregulation is associated with human and mouse cardiac fibrosis and heart failure, and ADAMTS15 is required for CF-to-MF activation.**

(**A**) *ADAMTS15* mRNA expression in failing human hearts (n=18) compared to non-failure hearts (n=8). 18S rRNA was used as a normalizer.

(**B**) *ADAMTS15* expression is correlated with the expression of *ACTA2* in human heart samples (n=26; 8 for NF and 18 for HF). Pearson correlation coefficient was presented. 18S rRNA was used as a normalizer.

(**C**) FAM114A1 protein expression is increased in failing human hearts (n=5) compared to non-failure hearts (n=5). ~100-130 cells with positive signals located in MI infarct areas were counted for the quantification. Scale bar: 10 μm.

(**D**) IF analysis and quantification of FAM114A1 protein expression in MI (20 days post-surgery) treated mouse heart tissue sections after Sham or MI surgery (n=6 for both groups). 100-150 cells per heart were counted for the quantification. Scale bar: 10 μm.

(**E**) IF quantification of COL1A1 and α-SMA protein expression after knockdown of *Adamts15* in TGFβ-treated primary mouse CFs. N=100-120 cells from three biological replicates were analyzed. Representative images are shown. Scale bar=20 μm.

Data were presented as mean ± SEM. ****P*<0.001. Comparisons of means between two groups were performed by unpaired two-tailed Mann Whitney test for A and unpaired Student *t* test for C, D.

**Figure S9. IF analysis of protein expression of MF marker α-SMA upon TGFβ stimulation after *FAM114A1* deletion.**

(**A**) Representative images of IF staining and quantification of normalized intensity of the CF activation marker (α-SMA) in isolated primary CFs from WT and *Fam114a1*$^{-/-}$ mouse hearts. CFs were treated with vehicle or TGFβ (10 nM) for 24 hrs, followed by serum starvation. For the quantification, 100-120 cells were analyzed for each group. Scale bar: 30 μm.

(**B**) Systolic blood pressure is lower in *Fam114a1*$^{-/-}$ mice than in WT control mice.

Data were presented as mean ± SEM. ns: not significant; ***P*<0.01; ****P*<0.001; *****P*<0.0001. Non-parametric Kruskal Wallis with Dunn's multiple comparison test was performed for A. Two-way ANOVA with Tukey's multiple comparisons test was performed for B. ***: 4 weeks (WT-Ang II vs. WT-Veh); **: 4 weeks (WT-Ang II vs. KO-Ang II); ns: 2 weeks (WT-Ang II vs. KO-Ang II) and 4 weeks (WT-Veh vs. KO-Veh).

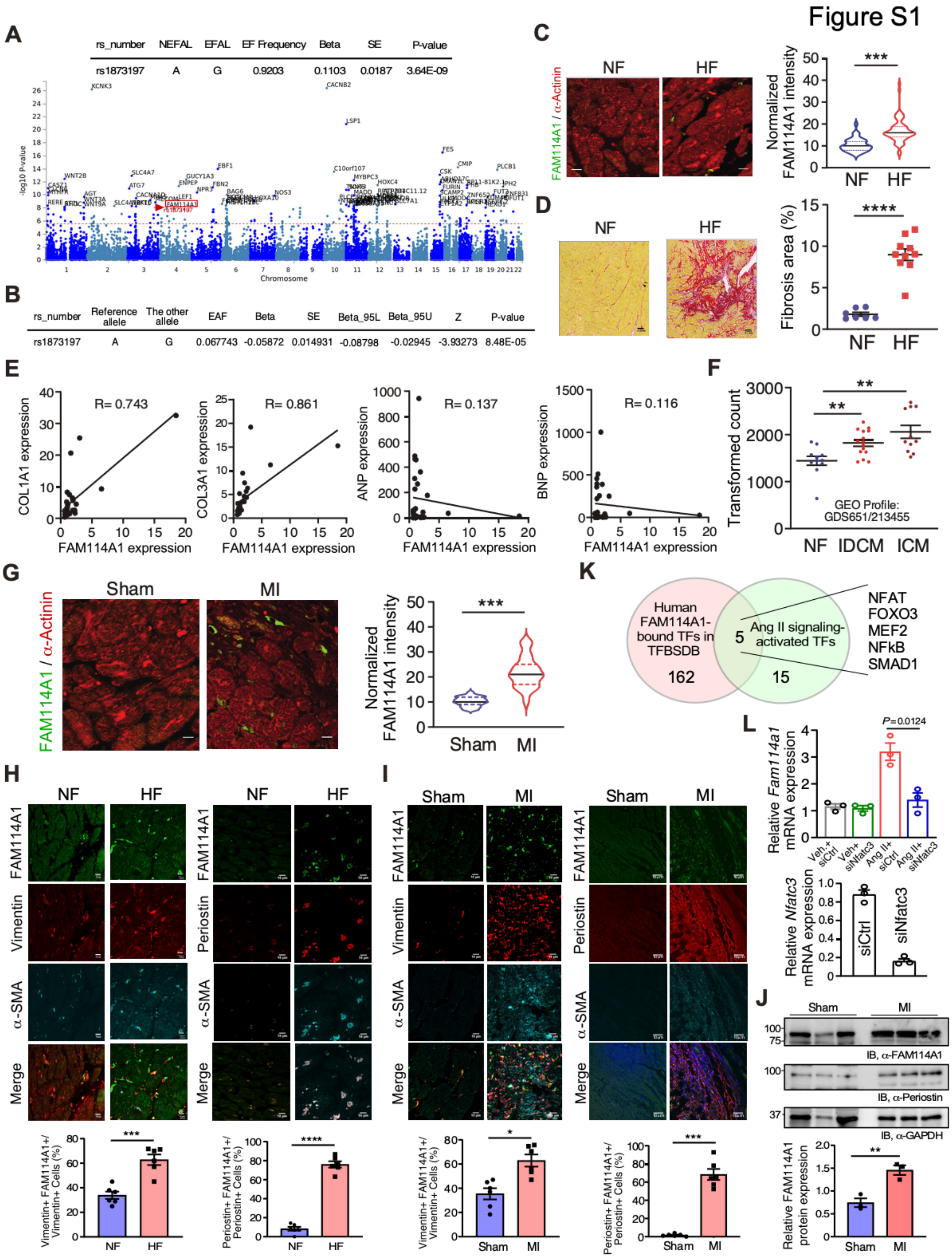
Figure S1
A
rs_number NEFAL EFAL EF Frequency Beta SE P-value
rs1873197 A G 0.9203 0.1103 0.0187 3.64E-09
-log10 P-value
Chromosome
FAM114A1
rs1873197
B
rs_number Reference allele The other allele EAF Beta SE Beta_95L Beta_95U Z P-value
rs1873197 A G 0.067743 -0.05872 0.014931 -0.08798 -0.02945 -3.93273 8.48E-05
C
NF HF
FAM114A1 / α-Actinin
Normalized FAM114A1 intensity
***
D
NF HF
Fibrosis area (%)
****
E
R= 0.743
R= 0.861
R= 0.137
R= 0.116
COL1A1 expression
COL3A1 expression
ANP expression
BNP expression
FAM114A1 expression
F
Transformed count
**
GEO Profile: GDS651/213455
NF IDCM ICM
G
Sham MI
FAM114A1 / α-Actinin
Normalized FAM114A1 intensity
***
K
Human FAM114A1-bound TFs in TFBSDB
162
5
Ang II signaling-activated TFs
15
NFAT
FOXO3
MEF2
NFkB
SMAD1
L
Relative Fam114a1 mRNA expression
P=0.0124
Veh+ siCtrl
Veh+ siNfatc3
Ang II+ siCtrl
Ang II+ siNfatc3
Relative Nfatc3 mRNA expression
siCtrl
siNfatc3
H
NF HF
FAM114A1
Vimentin
Periostin
α-SMA
Merge
Vimentin+ FAM114A1+/ Vimentin+ Cells (%)
Periostin+ FAM114A1+/ Periostin+ Cells (%)
I
Sham MI
J
Sham MI
IB, α-FAM114A1
IB, α-Periostin
IB, α-GAPDH
Relative FAM114A1 protein expression
**

Figure S2

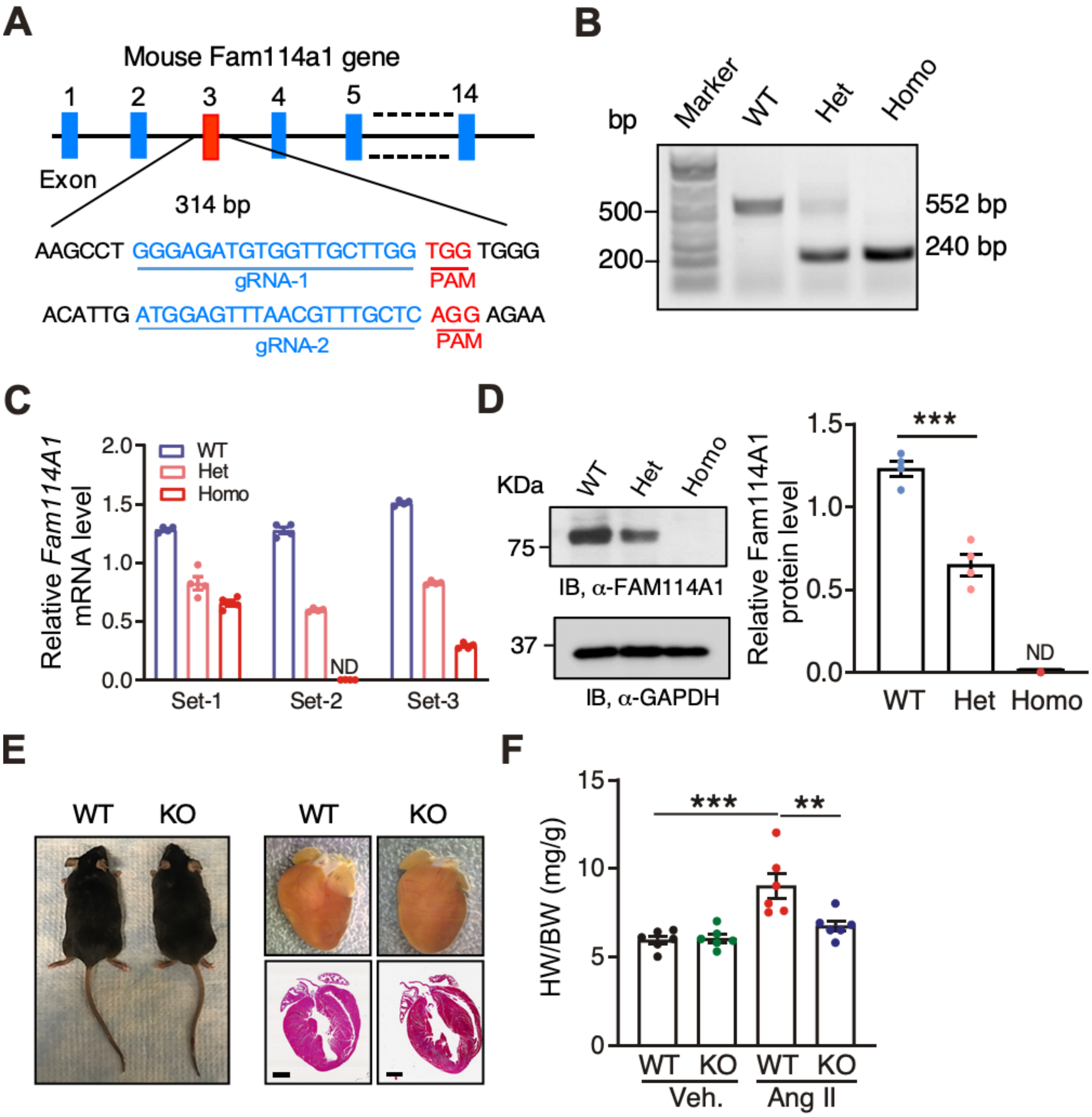
A
Mouse Fam114a1 gene
1
2
3
4
5
14
Exon
314 bp
AAGCCT GGGAGATGTGGTTGCTTGG TGG TGGG
gRNA-1
PAM
ACATTG ATGGAGTTTAACGTTTGCTC AGG AGAA
gRNA-2
PAM
B
bp
Marker
WT
Het
Homo
500
200
552 bp
240 bp
C
Relative Fam114A1 mRNA level
2.0
1.5
1.0
0.5
0.0
WT
Het
Homo
ND
Set-1
Set-2
Set-3
D
KDa
WT
Het
Homo
75
IB, α-FAM114A1
37
IB, α-GAPDH
Relative Fam114A1 protein level
1.5
1.0
0.5
0.0
***
ND
WT
Het
Homo
E
WT
KO
WT
KO
F
HW/BW (mg/g)
15
10
5
0
***
**
WT
KO
WT
KO
Veh.
Ang II

Figure S3

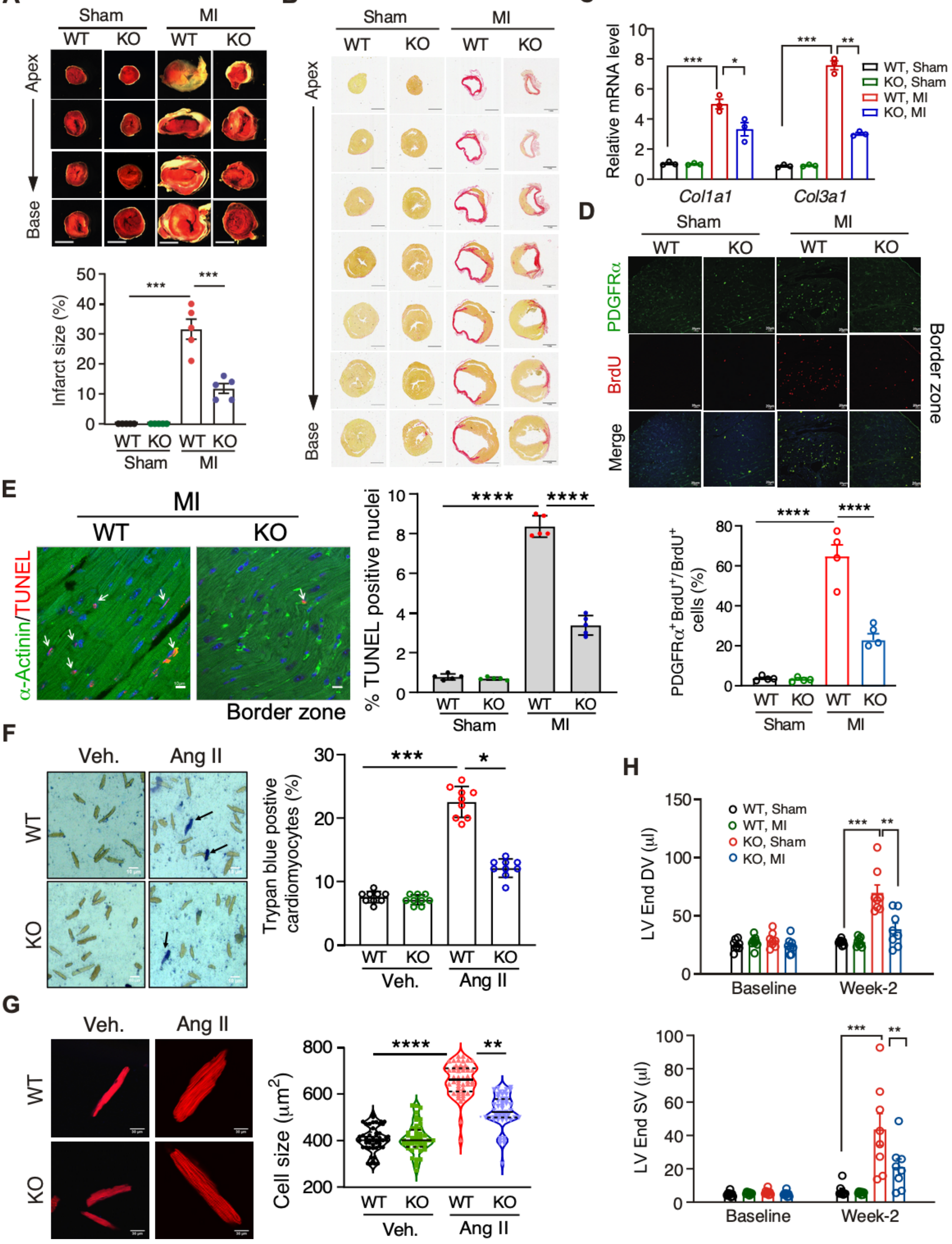
A
Sham
MI
WT
KO
Apex
Base
Infarct size (%)
***
B
Apex
Base
C
Relative mRNA level
Col1a1
Col3a1
WT, Sham
KO, Sham
WT, MI
KO, MI
D
PDGFRα
BrdU
Merge
Border zone
PDGFRα+BrdU+/BrdU+ cells (%)
****
E
α-Actinin/TUNEL
Border zone
% TUNEL positive nuclei
F
Veh.
Ang II
Trypan blue postive cardiomyocytes (%)
G
Cell size (μm2)
H
LV End DV (μl)
LV End SV (μl)
WT, Sham
WT, MI
KO, Sham
KO, MI
Baseline
Week-2

Figure S4

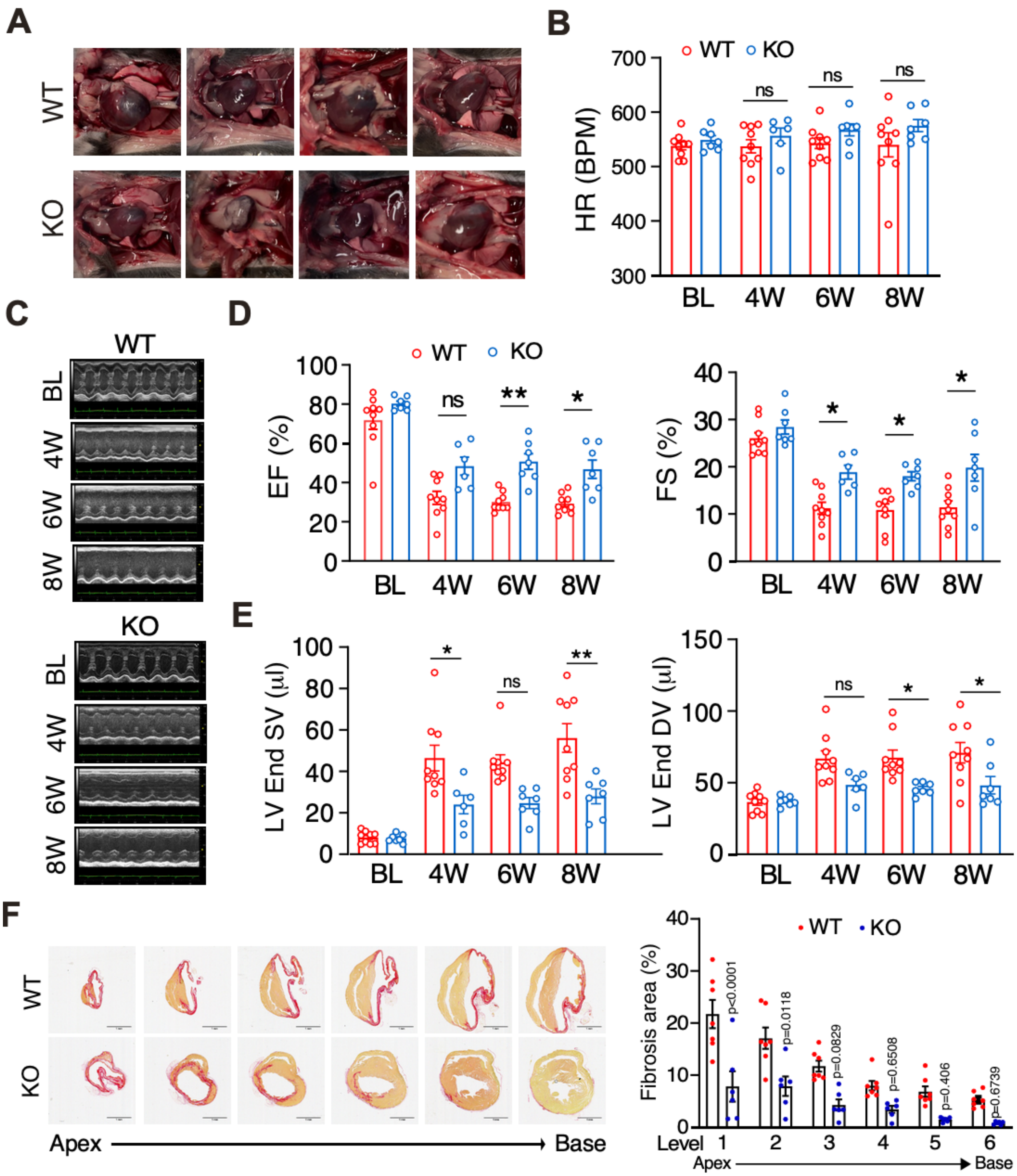

A
WT
KO
B
WT
KO
HR (BPM)
700
600
500
400
300
ns
ns
ns
BL
4W
6W
8W
C
WT
BL
4W
6W
8W
KO
BL
4W
6W
8W
D
WT
KO
EF (%)
100
80
60
40
20
0
ns
**
*
BL
4W
6W
8W
FS (%)
40
30
20
10
0
*
*
*
BL
4W
6W
8W
E
LV End SV (µl)
100
80
60
40
20
0
*
ns
**
BL
4W
6W
8W
LV End DV (µl)
150
100
50
0
ns
*
*
BL
4W
6W
8W
F
WT
KO
Apex
Base
Fibrosis area (%)
40
30
20
10
0
WT
KO
p<0.0001
p=0.0118
p=0.0829
p=0.6508
p=0.406
p=0.6739
Level
1
2
3
4
5
6
Apex
Base

Figure S5

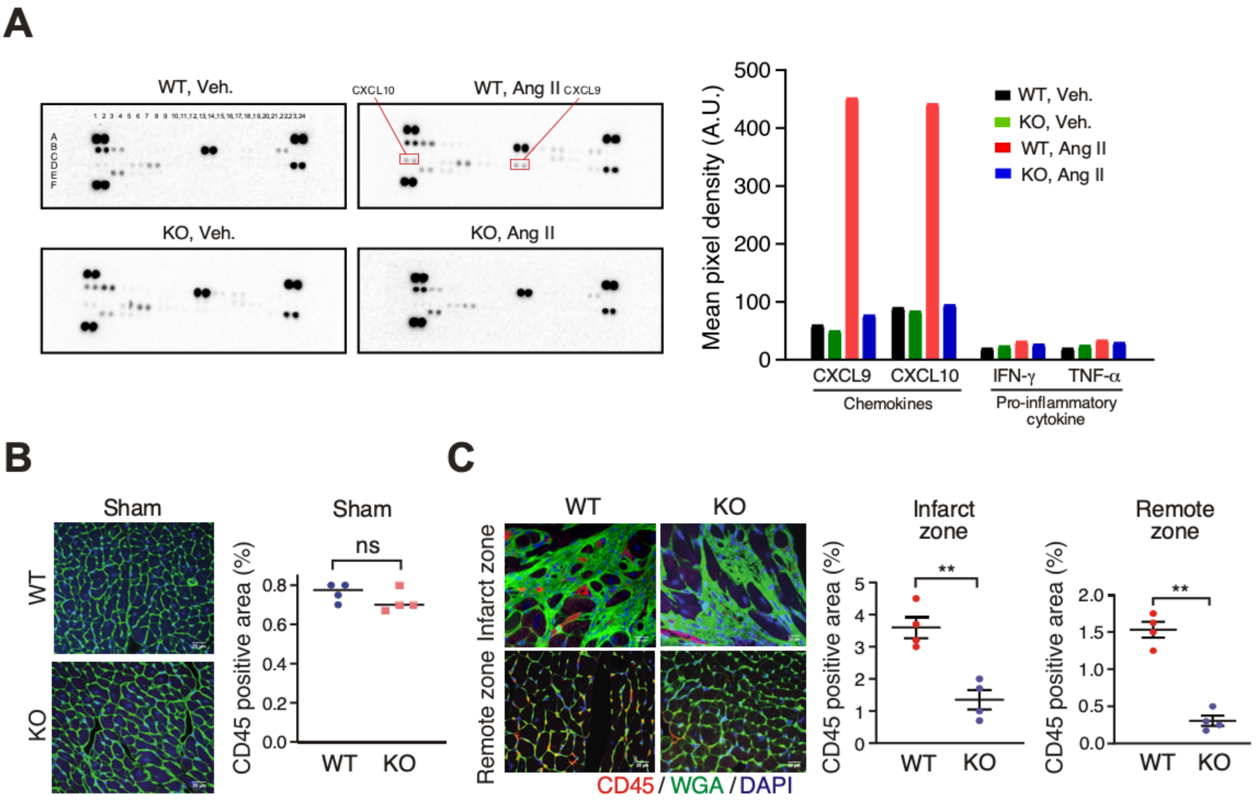
A
WT, Veh.
CXCL10
WT, Ang II
CXCL9
KO, Veh.
KO, Ang II
Mean pixel density (A.U.)
WT, Veh.
KO, Veh.
WT, Ang II
KO, Ang II
CXCL9
CXCL10
Chemokines
IFN-γ
TNF-α
Pro-inflammatory cytokine
B
Sham
WT
KO
CD45 positive area (%)
ns
C
Infarct zone
Remote zone
CD45 / WGA / DAPI
Infarct zone
Remote zone
**

Figure S6

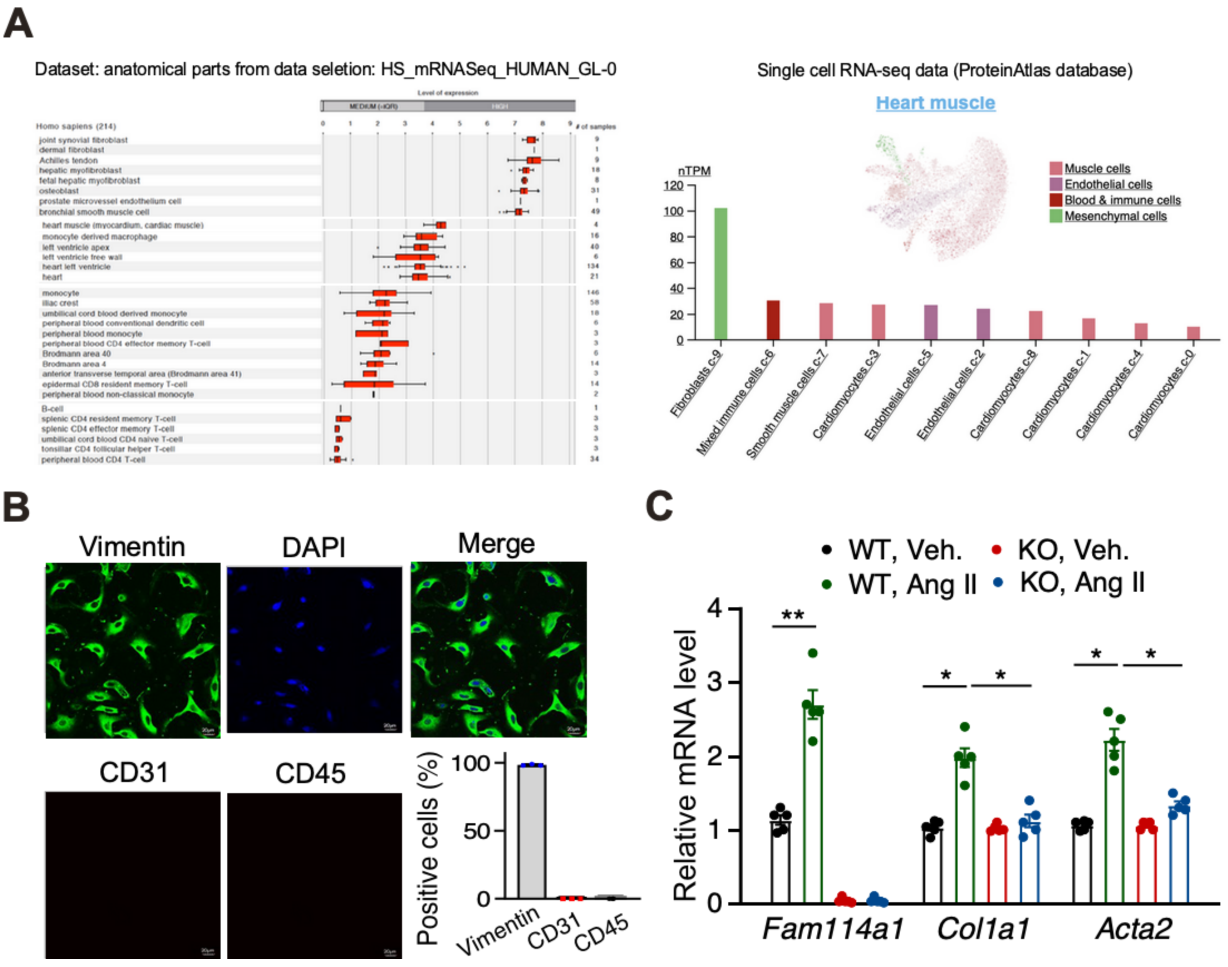

A
Dataset: anatomical parts from data seletion: HS_mRNASeq_HUMAN_GL-0
Single cell RNA-seq data (ProteinAtlas database)
Heart muscle
nTPM
Muscle cells
Endothelial cells
Blood & immune cells
Mesenchymal cells
Fibroblasts c-9
Mixed immune cells c-6
Smooth muscle cells c-7
Cardiomyocytes c-3
Endothelial cells c-5
Endothelial cells c-2
Cardiomyocytes c-8
Cardiomyocytes c-1
Cardiomyocytes c-4
Cardiomyocytes c-0
B
Vimentin
DAPI
Merge
CD31
CD45
Positive cells (%)
C
WT, Veh.
KO, Veh.
WT, Ang II
KO, Ang II
Relative mRNA level
Fam114a1
Col1a1
Acta2

Figure S7

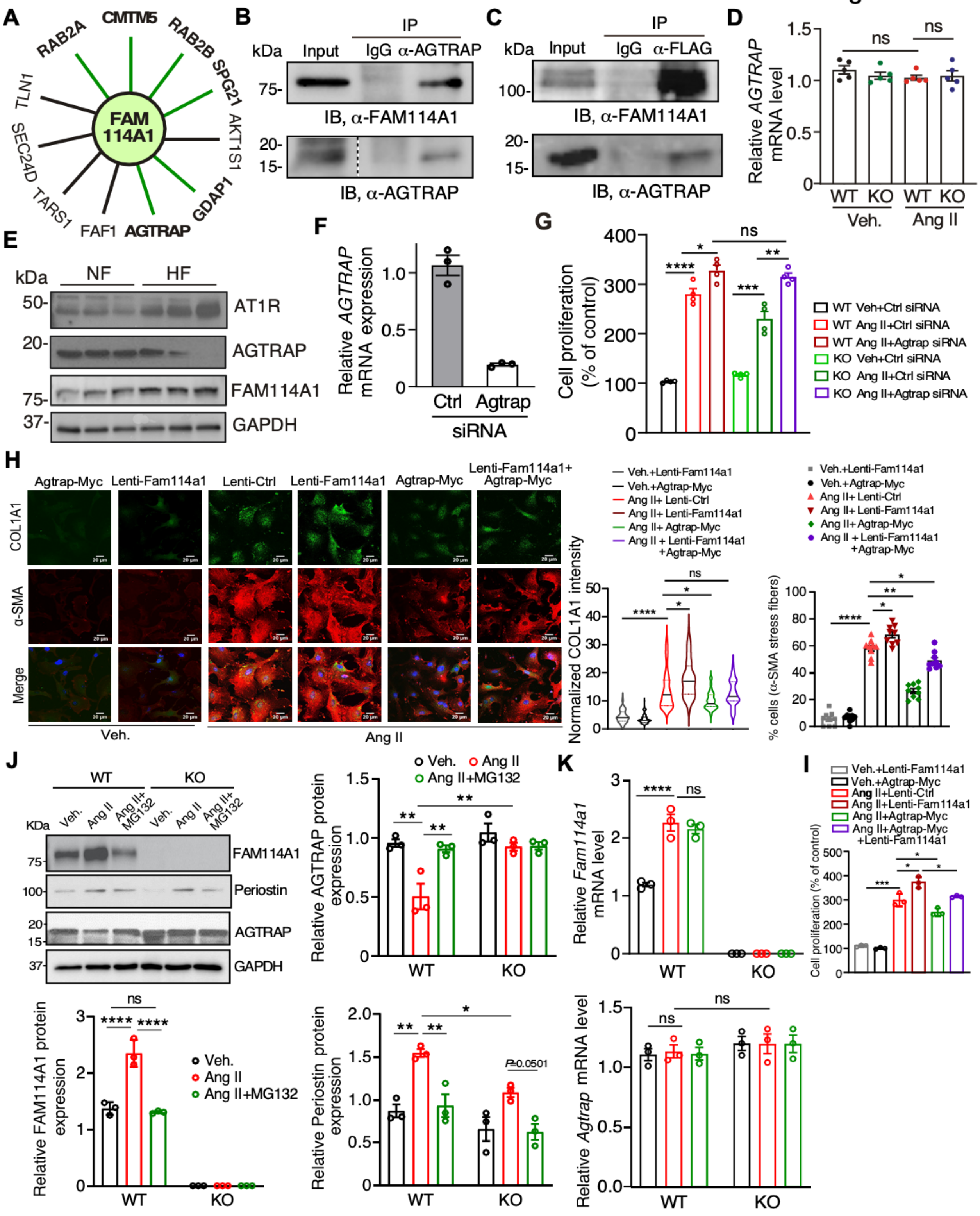
A
FAM 114A1
CMTM5
RAB2B
SPG21
AKT1S1
GDAP1
AGTRAP
FAF1
TARS1
SEC24D
TLN1
RAB2A
B
IP
kDa Input IgG α-AGTRAP
IB, α-FAM114A1
IB, α-AGTRAP
C
IP
kDa Input IgG α-FLAG
IB, α-FAM114A1
IB, α-AGTRAP
D
Relative AGTRAP mRNA level
WT KO WT KO
Veh. Ang II
E
kDa NF HF
AT1R
AGTRAP
FAM114A1
GAPDH
F
Relative AGTRAP mRNA expression
Ctrl Agtrap
siRNA
G
Cell proliferation (% of control)
WT Veh+Ctrl siRNA
WT Ang II+Ctrl siRNA
WT Ang II+Agtrap siRNA
KO Veh+Ctrl siRNA
KO Ang II+Ctrl siRNA
KO Ang II+Agtrap siRNA
H
Agtrap-Myc
Lenti-Fam114a1
Lenti-Ctrl
Lenti-Fam114a1
Agtrap-Myc
Lenti-Fam114a1+ Agtrap-Myc
COL1A1
α-SMA
Merge
Veh.
Ang II
Veh.+Lenti-Fam114a1
Veh.+Agtrap-Myc
Ang II+ Lenti-Ctrl
Ang II+ Lenti-Fam114a1
Ang II+ Agtrap-Myc
Ang II + Lenti-Fam114a1 +Agtrap-Myc
Normalized COL1A1 intensity
% cells (α-SMA stress fibers)
J
WT KO
KDa Veh. Ang II Ang II+ MG132 Veh. Ang II Ang II+ MG132
FAM114A1
Periostin
AGTRAP
GAPDH
Veh. Ang II Ang II+MG132
Relative AGTRAP protein expression
WT KO
K
Relative Fam114a1 mRNA level
WT KO
I
Veh.+Lenti-Fam114a1
Veh.+Agtrap-Myc
Ang II+Lenti-Ctrl
Ang II+Lenti-Fam114a1
Ang II+Agtrap-Myc
Ang II+Agtrap-Myc +Lenti-Fam114a1
Cell proliferation (% of control)
Relative FAM114A1 protein expression
Veh.
Ang II
Ang II+MG132
WT KO
Relative Periostin protein expression
P=0.0501
WT KO
Relative Agtrap mRNA level
WT KO

Figure S8

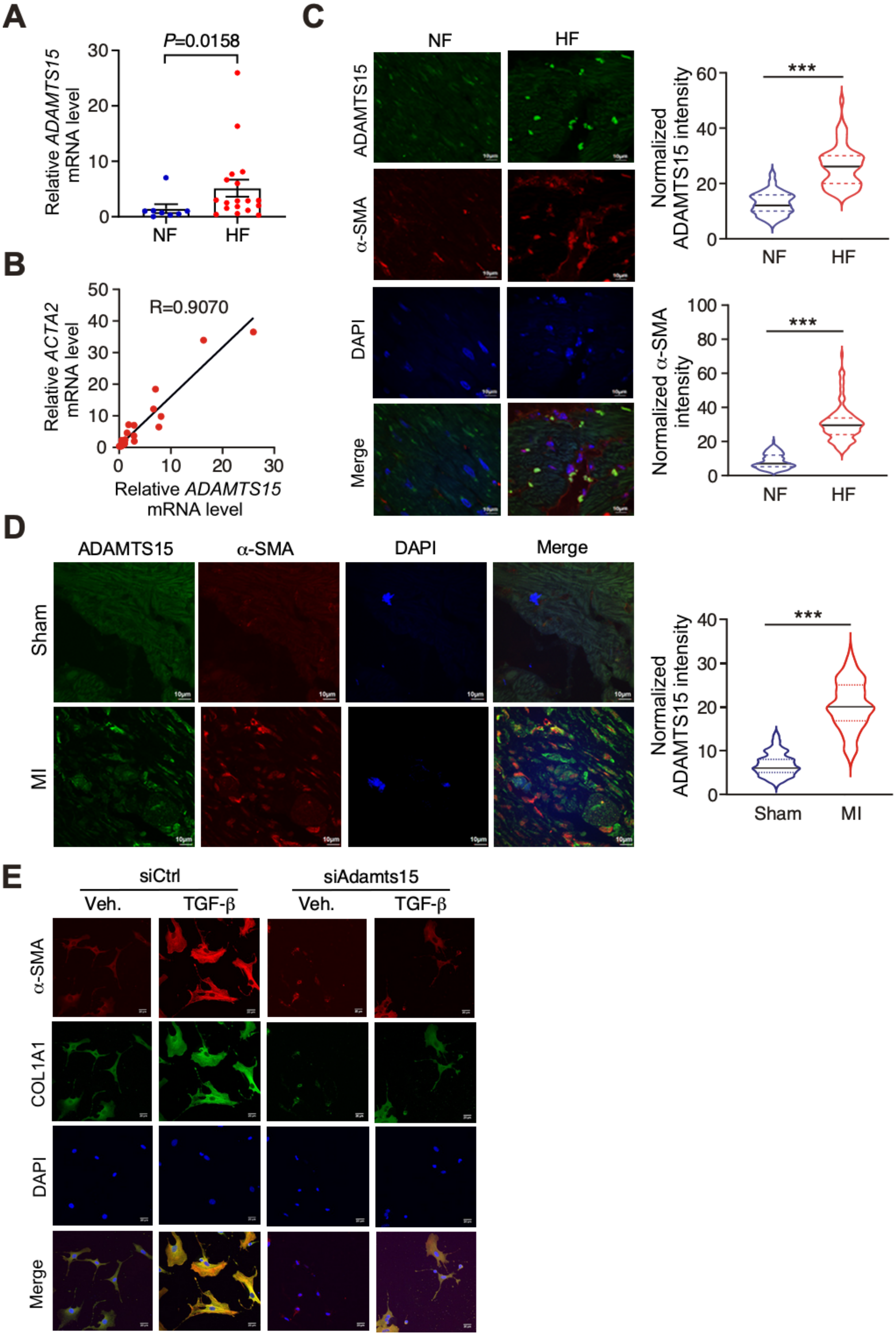
A
P=0.0158
Relative ADAMTS15 mRNA level
NF
HF
B
R=0.9070
Relative ACTA2 mRNA level
Relative ADAMTS15 mRNA level
C
NF
HF
ADAMTS15
α-SMA
DAPI
Merge
***
Normalized ADAMTS15 intensity
Normalized α-SMA intensity
D
ADAMTS15
α-SMA
DAPI
Merge
Sham
MI
E
siCtrl
siAdamts15
Veh.
TGF-β
COL1A1

Figure S9

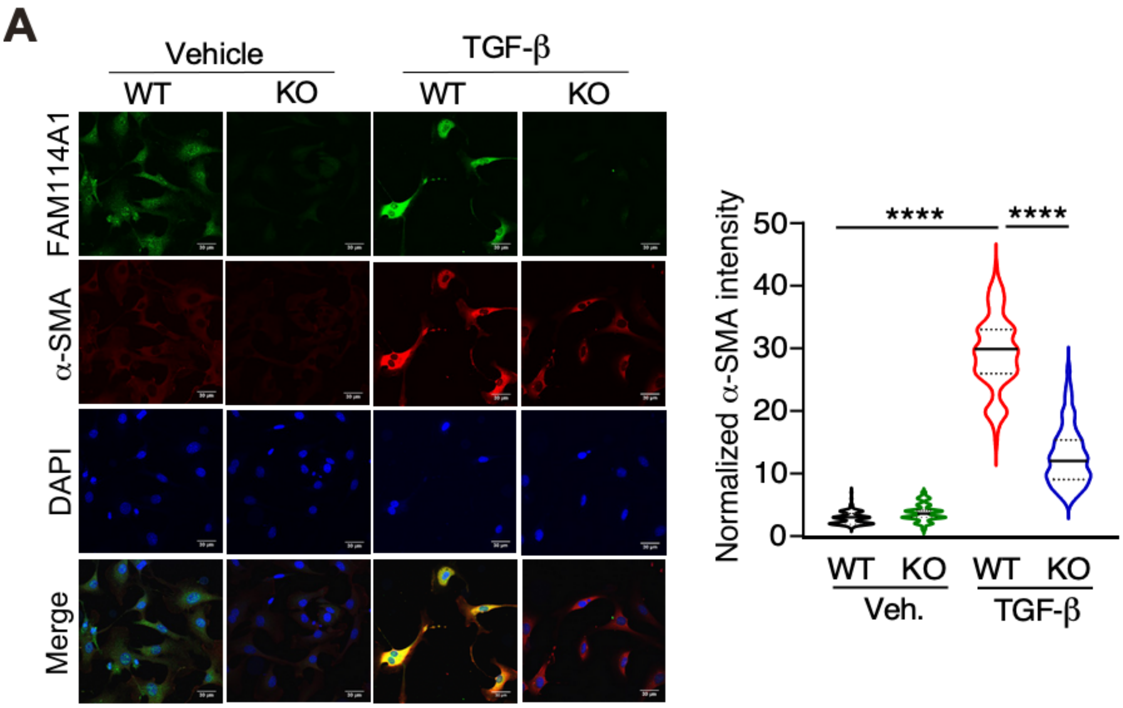
A
Vehicle
TGF-β
WT
KO
WT
KO
FAM114A1
α-SMA
DAPI
Merge
Normalized α-SMA intensity
50
40
30
20
10
0
****
****
WT KO WT KO
Veh.
TGF-β

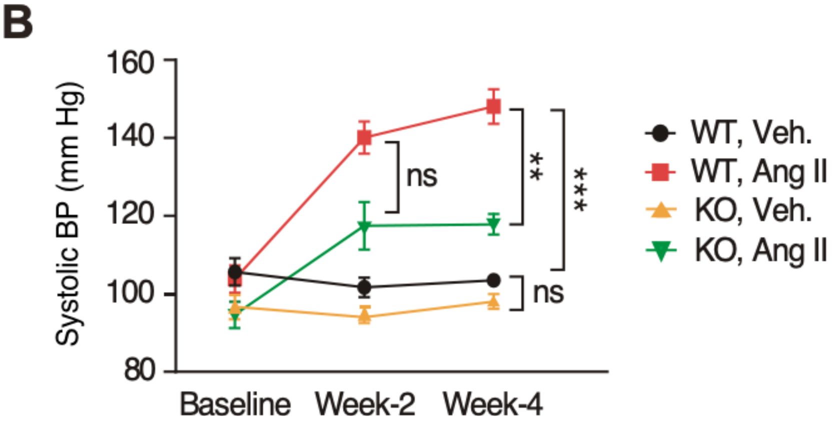
B
Systolic BP (mm Hg)
160
140
120
100
80
Baseline Week-2 Week-4
ns
**
***
ns
WT, Veh.
WT, Ang II
KO, Veh.
KO, Ang II

**Table S1**. Transcription factors identified in binding FAM114A1 DNA promoter region from TFBS Database (TFBSDB) and well-established Ang II signaling activated transcription factors(1).

| TFs binding to FAM114A1 gene | TFs downstream of Ang II signaling | Overlapped TFs |
|---|---|---|
| FOXB1 | AP1 | NFAT |
| POU4F2 | AP2a | FOXO3 |
| FOXJ2 | MEF2 | MEF2 |
| Sox1 | NFAT | NFkB |
| SOX10 | FOXO3A | SMAD1 |
| RARA | KLF2 | |
| SOX9 | KLF4 | |
| FOXG1 | HIF1a | |
| FOXC2 | GATA4 | |
| POU3F2 | NF-kB | |
| FOXL1 | CREB | |
| SOX8 | p53 | |
| CUX1 | PPARr | |
| EWSR1 | SMAD1 | |
| NFIX | SMAD2 | |
| Rarb | SMAD3 | |
| ZIC4 | SMAD5 | |
| POU4F3 | SMAD7 | |
| RARG | SRF | |
| REL | STAT3 | |
| NR4A2 | | |
| ONECUT1 | | |
| Foxc1 | | |
| FOXJ3 | | |
| FOXI1 | | |
| ZIC3 | | |
| Creb3l2 | | |
| NFAT | | |
| NFATC1 | | |
| Lhx3 | | |
| SOX7 | | |
| PBX1 | | |
| ZSCAN | | |
| TEAD3 | | |

| | | |
|---|---|---|
| POU1F1 | | |
| Foxd3 | | |
| CREB3L1 | | |
| SPDEF | | |
| SOX21 | | |
| TEAD1 | | |
| RORA | | |
| CPEB1 | | |
| THRB | | |
| POU3F3 | | |
| HMBOX1 | | |
| POU4F1 | | |
| RREB1 | | |
| HNF1A | | |
| DMRT4 | | |
| HOXA9 | | |
| FOXP1 | | |
| CEBPG | | |
| RXR | | |
| HNF3B | | |
| TST1 | | |
| DBX1 | | |
| TCF3 | | |
| NFKB | | |
| LEF1 | | |
| BTEB3 | | |
| FOXA2 | | |
| DMRT3 | | |
| CEBP | | |
| ZEC | | |
| HMGIY | | |
| EVI1 | | |
| BCL6 | | |
| FOXO4 | | |
| AR | | |
| SPDEF | | |
| SP1 | | |
| DR4 | | |
| GLI1 | | |

| | | |
|---|---|---|
| RP58 | | |
| FOXO3A | | |
| HNF3 | | |
| STAT6 | | |
| SPZ1 | | |
| HFH4 | | |
| OCTAMER | | |
| LHX3 | | |
| PRX2 | | |
| LYF1 | | |
| GM497 | | |
| TCFAP2E | | |
| PAX6 | | |
| LDSPOLYA | | |
| SRF | | |
| HNF6 | | |
| ELF4 | | |
| HMBOX1 | | |
| TCF3 | | |
| DBX2 | | |
| ZFP105 | | |
| HNF3G | | |
| NKX61 | | |
| CREL | | |
| ZBP89 | | |
| CDX2 | | |
| STAT5A | | |
| CDX2 | | |
| LHX2 | | |
| LXR | | |
| LEF1 | | |
| HNF1 | | |
| SOX18 | | |
| SFPI1 | | |
| PBX1 | | |
| NFE4 | | |
| CDX1 | | |
| OCT1. | | |
| UNCX4.1 | | |

| | | |
|---|---|---|
| HBP1 | | |
| PIT1 | | |
| TCF7 | | |
| RREB1 | | |
| PAX8 | | |
| CDC5 | | |
| NKX3A | | |
| HB24 | | |
| ARID3A | | |
| PMX2B | | |
| TBP | | |
| HIC1 | | |
| IK3 | | |
| SOX11 | | |
| BLIMP1 | | |
| PAX8 | | |
| FOXO1 | | |
| GR | | |
| SP4 | | |
| HFH8 | | |
| ZFP105 | | |
| HOXA7 | | |
| MTF1 | | |
| AP4 | | |
| FAC1 | | |
| BRN4 | | |
| FOXL1 | | |
| MYOGNF1 | | |
| GFI1B | | |
| CPHX | | |
| TCF7L2 | | |
| DMRT7 | | |
| FPM315 | | |
| HNF1B | | |
| PBX1 | | |
| PLZF | | |
| T3RBETA | | |
| ZFP281 | | |
| SOX14 | | |

| | | |
|---|---|---|
| HFH3 | | |
| EWSR1FLI1 | | |
| SOX2 | | |
| CTF1 | | |
| MEF2 | | |
| TFE | | |
| BRN3C | | |
| HOXD10 | | |
| DMRT1 | | |
| GLIS2 | | |
| FOXK1 | | |
| SMAD1 | | |
| NANOG | | |
| T3RALPHA | | |
| HOXC9 | | |
| TCF4 | | |

**Table S2**. Echocardiographic analysis (M-mode short axis) of WT and *Fam114a1*$^{-/-}$ mice (males) after 4 weeks of Ang II infusion.

| | **WT Veh.** | | ***Fam114a1*$^{-/-}$ Veh.** | | **WT Ang II** | | ***Fam114a1*$^{-/-}$ Ang II** | |
|---|---|---|---|---|---|---|---|---|
| | **BL** | **4 wk** | **BL** | **4 wk** | **BL** | **4 wk** | **BL** | **4 wk** |
| **Heart rate (bpm)** | 602.16 ±1.85 | 595.56 ±2.0 | 595.56 ±2.0 | 585.86 ±1.94 | 574.56 ±2.22 | 624.98 ±1.31$^{**}$ | 563.30 ±1.52 | 596.47 ±1.31$^{\#}$ |
| **EF (%)** | 78.30 ±0.87 | 76.02 ±1.50 | 76.02 ±1.50 | 76.54 ±1.10 | 76.50 ±0.56 | 56.94 ±2.50$^{**}$ | 76.14 ±1.50 | 69.90 ±0.96$^{\#}$ |
| **FS (%)** | 45.70 ±0.65 | 44.34 ±1.20 | 44.34 ±1.20 | 45.66 ±.0.67 | 45.30 ±0.67 | 30.32 ±1.10$^{**}$ | 46.20 ±1.51 | 38.40 ±1.20$^{\#}$ |
| **LVV,s (µl)** | 8.89 ±0.58 | 8.62 ±1.90 | 8.62 ±1.90 | 8.11 ±1.05 | 8.10 ±0.65 | 13.62 ±1.91$^{*}$ | 8.27 ±0.90 | 10.10 ±0.99$^{\#}$ |
| **LVV,d (µl)** | 40.93 ±1.74 | 40.13 ±2.40 | 40.13 ±2.40 | 41.02 ±1.20 | 39.32 ±2.50 | 51.84 ±2.41$^{*}$ | 40.82 ±2.31 | 44.56 ±1.76$^{\#}$ |
| **LVAWDs (mm)** | 1.10 ±0.02 | 1.10 ±0.05 | 1.10 ±0.05 | 1.11 ±0.03 | 1.09 ±0.02 | 1.13 ±0.03 | 1.09 ±0.01 | 1.10 ±0.01 |
| **LVAWDd (mm)** | 0.69 ±0.02 | 0.68 ±0.08 | 0.68 ±0.08 | 0.67 ±0.02 | 0.67 ±0.01 | 0.79 ±0.02 | 0.67 ±0.18 | 0.70 ±0.01 |
| **LVIDs (mm)** | 1.65 ±0.06 | 1.66 ±0.02 | 1.66 ±0.02 | 1.67 ±0.01 | 1.67 ±0.04 | 2.17 ±0.04 | 1.60 ±0.06 | 1.97 ±0.06 |
| **LVIDd (mm)** | 3.18 ±0.08 | 3.07 ±0.07 | 3.07 ±0.07 | 3.02 ±0.08 | 3.10 ±0.08 | 3.57 ±0.09 | 3.11 ±0.06 | 3.20 ±0.09 |
| **LVPWDs (mm)** | 0.85 ±0.03 | 0.84 ±0.02 | 0.84 ±0.02 | 0.84 ±0.01 | 0.87 ±0.02 | 0.93 ±0.02 | 0.81 ±0.02 | 0.86 ±0.01 |
| **LVPWDd (mm)** | 0.52 ±0.03 | 0.50 ±0.02 | 0.50 ±0.02 | 0.50 ±0.01 | 0.51 ±0.02 | 0.75 ±0.03$^{*}$ | 0.49 ±0.01 | 0.54 ±0.04 |
| **SV (µl)** | 32.50 ±0.74 | 31.86 ±0.53 | 31.86 ±0.53 | 29.90 ±0.62 | 31.02 ±0.83 | 43.83 ±2.62$^{**}$ | 31.92 ±0.47 | 35.30 ±0.49$^{\#}$ |
| **CO (ml/min)** | 19.57 ±0.61 | 20.59 ±0.31 | 20.59 ±0.31 | 18.05 ±0.48 | 17.80 ±0.63 | 26.82 ±0.34$^{*}$ | 21.85 ±0.51 | 21.71 ±0.45$^{\#}$ |
| **LV mass (mg)** | 59.63 ±0.73 | 60.42 ±0.60 | 60.42 ±0.60 | 60.96 ±0.69 | 60.13 ±1.26 | 71.60 ±0.70$^{**}$ | 58.92 ±0.82 | 63.53 ±0.67 |
| **BW (g)** | 19.12 ±0.36 | 19.98 ±0.39 | 19.98 ±0.39 | 20.16 ±0.44 | 19.73 ±0.39 | 18.68 ±0.32 | 19.96 ±0.44 | 20.67 ±0.45 |

| | |
|---|---|
| EF | Ejection fraction |
| FS | Fractional shortening |
| LVVs | Left ventricle volume, systolic |
| LVVd | Left ventricle volume, diastolic |
| LVAWDs | LV anterior wall diameter, systole |
| LVAWDd | LV anterior wall diameter, diastole |

| | |
|---|---|
| LVIDs | LV internal diameter, systole |
| LVIDd | LV internal diameter, diastole |
| LVPWDs | LV posterior wall diameter, systole |
| LVPWDd | LV posterior wall diameter, diastole |
| SV | Stroke volume |
| CO | Cardiac output |
| LV mass | Left ventricle mass |

Values are expressed as mean±SEM (n=6). *$P$<0.05 and **$P$<0.01: comparisons between WT-Veh. and WT-Ang II (4 wk). #$P$<0.05: comparisons between WT-Ang II (4 wk) and KO-Ang II (4 wk). Statistical analyses were performed using two-way ANOVA with Tukey's multiple comparisons test. BL: Baseline; wk: weeks.

**Table S3**. Echocardiographic analysis (B-mode long axis) of WT and *Fam114a1*$^{-/-}$ after permanent LAD ligation at baseline, 1 week, and 2 weeks post MI.

| | **WT Sham** | | | ***FAM114A1***$^{-/-}$ **Sham** | | | **WT MI** | | | ***FAM114A1***$^{-/-}$ **MI** | | |
|---|---|---|---|---|---|---|---|---|---|---|---|---|
| | **BL** | **1 wk** | **2 wk** | **BL** | **1 wk** | **2 wk** | **BL** | **1 wk** | **2 wk** | **BL** | **1 wk** | **2 wk** |
| **HR (BPM)** | 554.87 ±6.25 | 585.52 ±18.36 | 614.75 ±11.40 | 559.53 ±5.68 | 605.87 ±4.12 | 617.26 ±5.48 | 578.15 ±12.70 | 598.92 ±9.77 | 609.17 ±10.40 | 592.26 ±7.74 | 641.39 ±9.39 | 626.80 ±9.64 |
| **EF (%)** | 80.39 ±0.50 | 78.42 ±0.96 | 79.73 ±0.67 | 79.74 ±0.53 | 79.13 ±0.65 | 78.75 ±1.17 | 79.06 ±0.83 | 29.15 ±3.96* | 24.80 ±3.59** | 77.06 ±1.37 | 47.08 ±6.12 | 51.08 ±7.54# |
| **FS (%)** | 22.64 ±0.78 | 21.40 ±1.46 | 20.49 ±1.44 | 21.23 ±0.72 | 22.19 ±0.55 | 21.89 ±0.61 | 21.74 ±0.74 | 11.11 ±1.06* | 9.14 ±0.75** | 22.89 ±0.55 | 14.65 ±1.49 | 14.97 ±2.09# |
| **LV End, DV (μl)** | 24.56 ±1.60 | 24.06 ±0.83 | 27.03 ±0.80 | 27.12 ±1.76 | 27.94 ±1.27 | 27.17 ±1.38 | 28.73 ±2.24 | 69.65 ±6.83* | 69.42 ±6.71** | 24.01 ±2.35 | 43.46 ±5.06 | 38.19 ±5.27# |
| **LV End, SV (μl)** | 4.84 ±0.47 | 6.25 ±1.03 | 6.91 ±1.26 | 5.30 ±0.24 | 5.55 ±0.28 | 5.58 ±0.27 | 5.97 ±0.52 | 47.33 ±8.00* | 43.60 ±8.91** | 4.93 ±0.51 | 24.81 ±4.93 | 21.05 ±4.52# |

All values are expressed in mean ±SEM (n=8). EF: Ejection Fraction; FS: Fractional Shortening; LV End DV: Left ventricular end systolic volume; LV End SV: Left ventricular end diastolic volume. BL: Baseline; wk: week. **P*<0.05 ***P*<0.01: comparisons between WT-Sham and WT-MI. #p<0.05: comparisons between WT-MI and KO-MI. Statistical analyses were performed using two-way ANOVA with Tukey's multiple comparisons test. BL: Baseline; wk: weeks.

The number and gender of animals for echocardiography in the MI model in Table S3:

| | WT, Sham (n=8) | *Fam114a1*$^{-/-}$, Sham (n=8) | WT, MI (n=8) | *Fam114a1*$^{-/-}$, MI (n=8) |
|---|---|---|---|---|
| Number of males | 4 | 5 | 4 | 5 |
| Number of females | 4 | 3 | 4 | 3 |

**Table S4**. Echocardiographic analysis (B-mode long axis) of WT and *Fam114a1*$^{-/-}$ after permanent LAD ligation at baseline, 4 weeks, 6 weeks, and 8 weeks post MI.

| | **WT (n=9) MI** | | | | ***FAM114A1***$^{-/-}$ **(n=7) MI** | | | |
|---|---|---|---|---|---|---|---|---|
| | **BL** | **4 wk** | **6 wk** | **8 wk** | **BL** | **4 wk** | **6 wk** | **8 wk** |
| **HR (BPM)** | 538.20 ±7.27 | 537.46 ±12.17 | 543.17 ±10.02 | 540.01 ±22.14 | 549.24 ±7.45 | 556.67 ±13.16 | 567.48 ±11.12 | 575.24 ±11.09 |
| **EF (%)** | 71.78 ±4.69 | 32.19 ±3.31 | 30.11 ±1.58 | 29.31 ±1.62 | 80.28 ±1.26 | 48.36 ±4.24 | 50.67 ±4.01** | 46.78 ±4.63* |
| **FS (%)** | 26.04 ±1.18 | 11.22 ±1.24 | 10.85 ±1.29 | 11.42 ±1.35 | 28.42 ±1.40 | 18.85 ±1.36* | 18.01 ±0.95* | 19.80 ±2.81* |
| **LV End, DV (μl)** | 36.62 ±2.28 | 66.94 ±5.36 | 67.54 ±5.28 | 70.94 ±7.08 | 37.11 ±1.28 | 48.60 ±3.45 | 45.81 ±1.65* | 48.25 ±6.07* |
| **LV End, SV (μl)** | 8.12 ±0.96 | 46.43 ±6.22 | 44.38 ±3.61 | 56.10 ±6.90 | 7.79 ±0.89 | 23.31 ±4.91* | 25.40 ±3.06 | 25.90 ±3.48** |

All values are expressed in mean ±SEM (n=9 for WT and n=7 for KO). EF: Ejection Fraction; FS: Fractional Shortening; LV End DV: Left ventricular end systolic volume; LV End SV: Left ventricular end diastolic volume. BL: Baseline; wk: week. *$P$<0.05 **$P$<0.01: comparisons between WT-Sham and WT-MI. #p<0.05: comparisons between WT-MI and KO-MI. Statistical analyses were performed using two-way ANOVA with Tukey's multiple comparisons test.

The number and gender of animals for echocardiography in the MI model in Table S4:

| | WT, MI (n=9) | *Fam114a1*$^{-/-}$, MI (n=7) |
|---|---|---|
| Number of males | 5 | 4 |
| Number of females | 4 | 3 |

**Table S5**. Differentially expressed genes in Fam114a1 null cardiac fibroblasts compared to WT CF cells identified by RNA-Seq.

| Downregulated genes: | | | | | | |
|---|---|---|---|---|---|---|
| Gene symbol | baseMean | log2FoldChange | lfcSE | stat | pvalue | padj |
| Ifi208 | 336.445499 | -3.3030428 | 0.30343632 | -11.270495 | 1.84E-29 | 2.68E-25 |
| Fam114a1 | 420.980292 | -1.9289519 | 0.22134545 | -8.6379111 | 5.73E-18 | 4.17E-14 |
| Zfp366 | 90.4649281 | -1.7506059 | 0.29125241 | -5.7108675 | 1.12E-08 | 2.54E-05 |
| Adgrl4 | 149.466056 | -1.3803042 | 0.24945404 | -5.4054233 | 6.47E-08 | 0.00011786 |
| Col4a3 | 688.7584 | -1.2871402 | 0.24598038 | -5.060323 | 4.19E-07 | 0.00055488 |
| Ptprb | 646.554474 | -1.3587641 | 0.25644542 | -4.9683583 | 6.75E-07 | 0.00075744 |
| Adamts15 | 157.548437 | -1.2886709 | 0.25621539 | -4.8467208 | 1.26E-06 | 0.00114403 |
| Hmcn1 | 513.230538 | -1.2942578 | 0.26973617 | -4.4409816 | 8.95E-06 | 0.00621857 |
| Cdh13 | 157.214452 | -1.0646798 | 0.24073082 | -4.3005064 | 1.70E-05 | 0.00920394 |
| Hist1h4c | 57.8534824 | -1.2592517 | 0.28395817 | -4.077528 | 4.55E-05 | 0.0204676 |
| Palmd | 111.518489 | -1.2311616 | 0.28601587 | -4.046779 | 5.19E-05 | 0.02103485 |
| Pgm5 | 115.90321 | -1.1929564 | 0.28045557 | -4.018889 | 5.85E-05 | 0.02166309 |
| Sacs | 194.885889 | -1.1081813 | 0.26164945 | -4.0151952 | 5.94E-05 | 0.02166309 |
| Olfr558 | 9.10835853 | -0.7521089 | 0.23441053 | -3.9726664 | 7.11E-05 | 0.02410351 |
| Igf2 | 22.9823625 | -1.2451641 | 0.2922767 | -3.9345863 | 8.33E-05 | 0.02642063 |
| Rgs6 | 60.4677702 | -1.1544234 | 0.29179101 | -3.9238396 | 8.71E-05 | 0.02647689 |
| Hist1h1d | 64.7122979 | -1.0643808 | 0.26643229 | -3.9183283 | 8.92E-05 | 0.02652963 |
| Prrt4 | 87.7719185 | -1.1554315 | 0.29162004 | -3.9135188 | 9.10E-05 | 0.02652963 |
| Slc38a4 | 104.284925 | -1.0468021 | 0.26362301 | -3.9051242 | 9.42E-05 | 0.02692908 |
| Itga1 | 353.785684 | -0.9433694 | 0.23429455 | -3.8972571 | 9.73E-05 | 0.02728377 |
| Rgs5 | 1951.74483 | -1.2246132 | 0.29992052 | -3.8926237 | 9.92E-05 | 0.0272856 |
| Itga6 | 272.988868 | -1.0915133 | 0.28851931 | -3.7443383 | 0.00018087 | 0.04186705 |
| Serpinc1 | 94.8932021 | -1.1042097 | 0.30196851 | -3.6707081 | 0.00024188 | 0.04641221 |
| Vash1 | 128.427452 | -1.1568177 | 0.29291302 | -3.6623661 | 0.0002499 | 0.04732777 |
| Adgrf5 | 1167.32955 | -1.0681848 | 0.27777687 | -3.6483694 | 0.00026391 | 0.04825211 |
| Hook3 | 865.527592 | -0.6852541 | 0.18141298 | -3.644404 | 0.00026801 | 0.04825211 |
| Ralgapa2 | 269.651411 | -0.8913907 | 0.23156471 | -3.6368806 | 0.00027596 | 0.04907709 |
| | | | | | | |
| Upregulated genes: | | | | | | |
| Hspb1 | 1813.10504 | 1.4428178 | 0.22490545 | 6.39180813 | 1.64E-10 | 7.97E-07 |
| Per2 | 693.365945 | 0.91721376 | 0.19839291 | 4.71777455 | 2.38E-06 | 0.00204538 |
| Efemp1 | 1498.51109 | 1.22599744 | 0.26708562 | 4.4232357 | 9.72E-06 | 0.00644526 |
| Kcnip1 | 59.638558 | 1.36213787 | 0.30621496 | 4.39091174 | 1.13E-05 | 0.00685865 |

| | | | | | | |
|---|---|---|---|---|---|---|
| Aebp1 | 2874.2104 | 0.76393339 | 0.17482729 | 4.37423384 | 1.22E-05 | 0.00710832 |
| Gsta3 | 1085.19349 | 1.22470554 | 0.28308745 | 4.1722663 | 3.02E-05 | 0.01570719 |
| Podn | 1117.25604 | 1.13774542 | 0.26830775 | 4.10138121 | 4.11E-05 | 0.01996371 |
| Arc | 452.674352 | 1.0295254 | 0.26322897 | 4.05439654 | 5.03E-05 | 0.02094281 |
| Egfr | 1705.9345 | 0.82130466 | 0.20886299 | 4.00982461 | 6.08E-05 | 0.02166309 |
| Hbb-bs | 251.788042 | 0.06065555 | 0.1275473 | -4.0162271 | 5.91E-05 | 0.02166309 |
| Serpina3n | 1413.70826 | 1.18558645 | 0.2814697 | 4.00927435 | 6.09E-05 | 0.02166309 |
| C4b | 894.519554 | 1.09060376 | 0.27821157 | 3.80236244 | 0.00014332 | 0.03620852 |
| Wif1 | 21.0972673 | 0.69195456 | 0.2802807 | 3.80117811 | 0.00014401 | 0.03620852 |
| Vit | 416.245056 | 1.01133101 | 0.26117814 | 3.77709756 | 0.00015867 | 0.03793172 |
| Rpl28 | 3324.10385 | 0.79552214 | 0.20443925 | 3.74779829 | 0.00017839 | 0.04186705 |
| Dennd2a | 1221.38218 | 0.67814079 | 0.18359232 | 3.70939183 | 0.00020776 | 0.04498988 |
| Rplp0 | 8889.34972 | 0.66218671 | 0.17421412 | 3.70325082 | 0.00021285 | 0.04498988 |
| Rpl18a | 4861.06529 | 0.68618757 | 0.18065189 | 3.69941473 | 0.0002161 | 0.04501923 |
| Hba-a2 | 71.9194931 | 0.04814612 | 0.10583483 | -3.679164 | 0.000234 | 0.04627632 |
| Ppard | 742.019363 | 0.90169483 | 0.24281811 | 3.62926451 | 0.00028423 | 0.04993885 |

## SI Materials and Methods

### Reagents, antibodies, plasmids, and siRNAs

Human angiotensin II (Cat. No. A9525), E-64d (Cat. No. E8640), 2,3 Butanedione Monoxime (Cat. No. B0753), and 5-Bromo-2'-deoxyuridine (Cat. No. 10280879001) were purchased from Sigma Aldrich, USA. Trypan blue (Cat. No. 97063-702) was purchased from VWR. Alexa Fluor™ 594 Phalloidin (Cat. No. No. A12381) was from Thermo Fisher Scientifics. MG132 (Cat. No. 474790) was obtained from Calbiochem, USA. The type II collagenase was purchased from Worthington company (Cat. No. LS004177). Taurin (Cat. No. 1665100) was purchased from Acros Organics. In situ cell death detection kit TMR red (Cat. No. 12156792910) was procured from the Roche company. The antifade mounting medium with DAPI (Cat. No. H-1500) was obtained from Vectorlabs company.

The Myc-AGTRAP plasmid (Cat. No. MG53324-CM) was purchased from Sino Biological and AT1R-P2A-mCherry plasmid (Cat. No. 112934) was from Addgene. siRNAs including mouse Fam114a1 (Cat. No. 4392421), mouse Agtrap (Cat. No. 4331182), mouse Nfatc3 (Cat. No. 4390771), and negative control siRNA were procured from ThermoFisher Scientifics and transfected into cardiac fibroblasts by lipofectamine 3000 (ThermoFisher) following the instruction manual.

The overexpression vector and lentivirus (titer >$10^8$TU/ml) of pLV[Exp]-EGFP:T2A:Puro-EF1A>mFam114a1[NM_026667.3] (LVM(VB900013-3673muy)-C) and control GFP-expressing lentivirus (VB010000-9298rtf)-C) were purchased from VectorBuilder and used for infection in primary mouse cardiac fibroblasts.

The FAM114A1 overexpression vector for NIH/3T3 cell transfection and IF in Figure 6B is pFam114a1-EGFP. The recombinant FAM114A1 protein contains an EGFP fusion protein in the C-terminus. Mouse Fam114a1 cDNA was cloned in the pEGFP-N1 vector (Clontech) using primers as follows.

Forward primer (SalI): ACGC<u>GTCGAC</u> ATGTCTGATGATGCTCGTGAC

Reverse primer (XmaI): CCC<u>CCCGGG</u>A GCTGCAATCTGGCTGGGCTG

The FAM114A1 overexpression vector for NIH/3T3 cell transfection and IP-IB in Figure S7C is pSFB-mFam114a1-IRES-EGFP. The recombinant FAM114A1 protein contains a N-terminal 2xFLAG tag. Mouse Fam114a1 cDNA was cloned in the vector using primers as follows.

Forward primer (SalI): ACGC<u>GTCGAC</u>GG ATGTCTGATGATGCTCGTGAC

Reverse primer (XmaI): CCC<u>CCCGGG</u> CTAGCTGCAATCTGGCTGGG

Antibodies used in this study:

| Primary antibody | Cat. No. and vendor | Dilution | Detection |
|---|---|---|---|
| Rabbit anti-FAM114A1 | 21638-1-AP; ProteinTech | IF (1:200)<br>WB (1:1000) | NIH/3T3; Cardiac fibroblasts; Mouse and human heart tissue sections |
| Rabbit anti-FAM114A1 | TA335404; Origene | WB (1:1000) | Mouse heart tissue lysates in Fig. S2D |
| Rabbit anti-AGTRAP | 11559-1-AP; ProteinTech | WB (1:1000) | NIH/3T3; Cardiac fibroblasts |
| Rabbit anti-AT1R | Ab18801; Abcam | WB (1:1000) | NIH/3T3; Cardiac fibroblasts |
| Rabbit anti-CMTM5 | PA5-62589; ThermoFisher | WB (1:1000) | Heart tissue lysates |
| Rabbit anti-SPG21 | PA5-59176; ThermoFisher | WB (1:1000) | Heart tissue lysates |
| Mouse anti-RAB2B | sc-81921; Santa Cruz | WB (1:500) | Heart tissue lysates |
| Mouse anti-vimentin | 60330-1-Ig; ProteinTech | IF (1:300) | Mouse and human heart tissue sections; CFs |
| Mouse anti-GAPDH | 60004-1-Ig; ProteinTech | WB (1:5000) | Multiple cell types |
| Mouse anti-FLAG | 66008-2-Ig; ProteinTech | WB (1:1000) | Multiple cell types |
| Mouse anti-α-Tubulin | 66031-1-Ig; ProteinTech | WB (1:5000) | Multiple cell types |
| Goat anti-Mouse AF488 | A11001; Life Technologies | IF (1:500) | Multiple cell types |
| Goat anti-Rabbit AF488 | A11034; Life Technologies | IF (1:500) | Multiple cell types |
| Goat anti-Mouse AF594 | A1105; Invitrogen | IF (1:500) | Multiple cell types |
| Goat anti-Rabbit AF594 | A11012; Invitrogen | IF (1:500) | Multiple cell types |
| Rabbit anti-COL1A1 | SAB2109131; Sigma Aldrich | IF (1:300) | Myofibroblasts (MFs) |
| Mouse anti-Periostin | 66491-1-Ig; ProteinTech | IF (1:500) | Mouse and human heart tissue sections; MFs |
| Mouse anti-α-SMA (monoclonal) | A2547; Sigma Aldrich | IF (1:300)<br>WB (1:1000) | Mouse and human heart tissue sections; MFs |
| Goat anti-α-SMA (polyclonal) | PA5-18292; ThermoFisher | IF (1:500) | Mouse and human heart tissue sections; MFs |
| Mouse anti-CD45 | BDB56050; BD biosciences | IF (1:200) | Hematopoietic cells |
| Mouse anti-α-Actinin | A7811; Sigma Aldrich | WB (1:5000) | Cardiomyocytes |
| Mouse anti-CD31 | 3528; Cell Signaling Technology | IF (1:400) | Cardiac fibroblasts culture for EC detection |
| Wheat Germ Agglutinin AF-488 | W11261; Invitrogen | IF (1:1000) | Cardiomyocytes |
| Mouse anti-BrdU | 66241-1-Ig; ProteinTech | IF (1:200) | Proliferating cells |
| Mouse anti-PDGFRα | AF1062-SP; R&D Systems | IF (1:100) | Cardiac fibroblasts |
| Mouse anti-Cardiac Troponin | MA5-12960; ThermoFisher | IF (1:200) | Cardiomyocytes |
| Rabbit ERK1/2 Polyclonal antibody | 11257-1-AP; ProteinTech | WB (1:1000) | Cardiac fibroblasts |
| Rabbit phospho-ERK1/2 (Thr202/Tyr204) polyclonal antibody | 28733-1-AP; ProteinTech | WB (1:1000) | Cardiac fibroblasts |
| Mouse AKT monoclonal antibody | 60203-2-Ig; ProteinTech | WB (1:1000) | Cardiac fibroblasts |

| | | | |
|---|---|---|---|
| Mouse phospho-AKT (Ser473) monoclonal antibody | 66444-1-Ig; ProteinTech | WB (1:1000) | Cardiac fibroblasts |
| Rabbit p38 MAPK antibody | 9212; Cell Signaling Technology | WB (1:1000) | Cardiac fibroblasts |
| Rabbit phospho-p38 MAPK (Thr180/Tyr182) antibody | 9211; Cell Signaling Technology | WB (1:1000) | Cardiac fibroblasts |

Vector maps for mouse Fam114a1-overexpressing lentivirus constructs used in this study (generated by VectorBuilder): pLV[Exp]-EGFP:T2A:Puro-EF1A>mFam114a1 ($4.43 \times 10^8$ TU/ml)

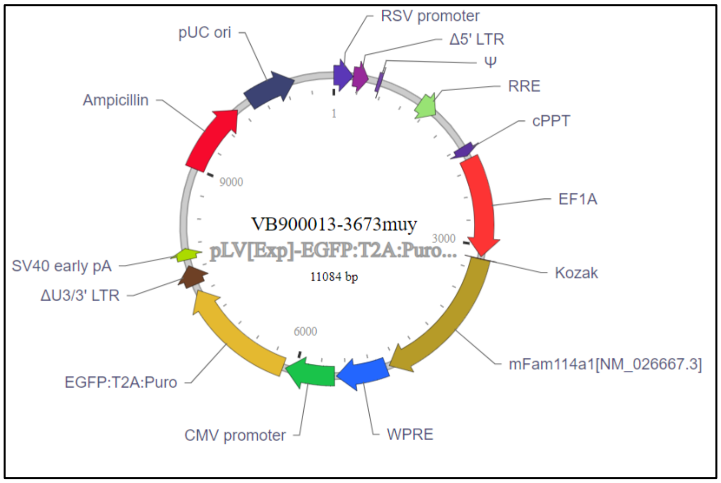


## Human tissue samples

All human samples of frozen cardiac tissues, including 20 samples from explanted failing hearts (10 ischemic heart failure and 10 dilated cardiomyopathy hearts) and 8 samples from non-failing donor hearts, as well as paraffin section slides from dilated cardiomyopathy (DCM; n=5), ischemic heart failure (ISHF; n=5) and non-failing hearts (n=7) were acquired from the Cleveland Clinic. All surgical procedures and tissue harvesting were performed following the Cleveland Clinic procedures and guidelines. This study was approved by Material Transfer Agreement between the URMC and the Cleveland Clinic. In Figure 1A, total RNA samples from two failing hearts showed degradation during the quality control process and were excluded from RT-qPCR analysis. All human samples were picked up randomly based on the presence or absence of heart failure by our collaborator, Dr. Waihong Wilson Tang at Cleveland Clinic. We are blinded from any clinical data. The identity numbers (IDs) of the frozen cardiac and histology tissues are listed below.

**Cardiac Tissue**

| **Non-Failing ID** | **Dilated Failing ID** | **Ischemic Failing ID** |
|---|---|---|
| D44 | X2870 | X2854 |
| D156 | X2866 | X2834 |
| D158 | X2860 | X2749 |
| D188 | X2853 | X2679 |
| D204 | X2848 | X2614 |

| | | |
|---|---|---|
| D214 | X2846 | X2607 |
| D218 | X2833 | X2590 |
| D247 | X2823 | X2549 |
| | X2768 | X2445 |
| | X2755 | X2348 |

**Histology Tissue**

| **Non-Failing ID** | **Dilated Failing ID** | **Ischemic Failing ID** |
|---|---|---|
| D195 | X2670 | X2669 |
| D199 | X2690 | X2732 |
| D214 | X2698 | X2739 |
| D222 | X2675 | X2712 |
| D226 | X2659 | X2679 |
| D237 | | |
| D224 | | |

## Maintenance of experimental animals and mouse heart failure models

The University of Rochester Medical Center Animal Care and Use of Committee approved all experimental animal procedures. This study used WT C57BL/6J and *Fam114a1*$^{-/-}$ (global knockout with the C57BL/6J background) mice generated at Jackson Laboratories (IMPC program). They generated the mice by injecting the Cas9 RNA and four guide sequences ACCAAGCAACCACATCTCCC, AGATGTGGTTGCTTGGTGGT, TTTGACATAGCGTACATTGA, and ATGGAGTTTAACGTTTGCTC, which resulted in a 314 base pair of nucleotides deletion beginning at chromosome 5 positive-strand position 64,995,684 bp, GGGAGATGTGGTTGCTTGGT, and ending after GGAGTTTAACGTTTGCTCAG at 64,995,997 bp (GRCm38/mm10). The mutation deletes exon 3 and 226 bp of flanking intronic sequence, including the splice acceptor and donor, and is predicted to cause a change of amino acid sequence after residue 116 and early truncation 3 amino acids later. Mice were procured at 8 weeks of age and maintained in a vivarium facility and ad libitum free access to standard chow and water.

In this study, we used two mouse heart failure models, including angiotensin II (Ang II) osmotic minipump (ALZA Corporation, CA) implantation and myocardial infarction (MI) surgery (left anterior descending coronary artery ligation). In the entire study, we used age-matched male mice at ~8-12 weeks. All the mouse surgeries were done by the mouse Microsurgical Core facility at URMC. We used 6-8 mice from each genetic background for individual treatment groups to get the statistical significance of different groups of the study.

For the osmotic minipump implantation, WT and *Fam114a1*$^{-/-}$ male mice from sibling mating of heterozygous *Fam114a1*$^{+/-}$ mice at 8–12 weeks of age were subjected to subcutaneous infusion with vehicle saline or Ang II for 4 weeks using osmotic mini-pumps in a randomized and blinded manner by microsurgeons from the microsurgical core facility of Aab CVRI. Mice were anesthetized using 2.0% isoflurane and placed on a heated surgical board. A side/upper back area skin incision was made, and the mini-osmotic pump was inserted subcutaneously and set to deliver Ang II or vehicle at a rate of 1.4

mg/Kg/day. The incision was then closed with 6-0 coated vicryl in a subcuticular manner, and the animals were allowed to recover. The sutures were removed 2 weeks after the pumps were transplanted. The pumps were not removed and remained for 4 weeks. The animals were euthanized after 4 weeks of Ang II infusion, and mouse hearts were harvested for experiments, including RNA, protein extraction, and sectioning.

The LAD ligation-based MI surgery was performed by the Mouse Microsurgical Core of Aab CVRI(2). For MI surgery, male or female mice were placed on a heating pad, and the airway was stabilized by endotracheal intubation and mechanical ventilation provided (inspiratory tidal volume of 250 μL at 130 breaths/min). The mice were given SR Buprenorphine 2.5 mg/Kg via subcutaneous injection. Isoflurane flow was continually maintained at approximately 1.5% along with oxygen. A midline cervical incision was made to expose the trachea for intubation with a PE90 plastic catheter. The catheter was connected to a Harvard mini vent supplying supplemental oxygen with a tide volume of 225-250 μl and a respiratory rate of 130 strokes/min. Surgical plane anesthesia was subsequently maintained at 1-1.5% isoflurane. The skin was incised and the chest cavity opened at the level of the 4th intercostal space. Oral intubation was employed by placing PE 90 tubing in the mouth and advancing slowly into the trachea. Mechanical P.I. ventilation (tidal volume of approximately 0.4 ml at 130 breaths/min) was then begun. After intubation, a midline incision was made between the sternum and the left internal mammalian artery. Alternatively, a lateral incision (left thoracotomy) was made in the fourth intercostal space. The mouse heart was exposed and the left coronary artery branch points were visualized under 10x magnification before ligation, and the LAD coronary artery was ligated intramurally 2 mm from its ostial origin for standard MI with a 9-0 proline suture. Transmural ischemia was assured by color loss on the left ventricle wall and ST-segment elevation noted on the electrocardiogram. Lungs were inflated and the chest was closed in two layers; the ribs (inner layer) were closed with 6-0 coated vinyl sutures in an interrupted pattern. The skin was closed using 6-0 nylon or silk sutures in a subcuticular manner. The anesthesia was stopped and once the mouse was breathing on its own, the mouse was removed from the ventilator and allowed to recover in a clean cage on a heated pad. A sham operation was performed using the same procedure, but a suture was passed under the LAD coronary artery without ligation.

Anesthetic and analgesic agents used in the study are listed below. After Buprenorphine SR treatment, lab fellows inspected mice once a day for 3 days and registered in the recording card. Carbon dioxide euthanasia is used for terminating mice.

| Drug or drug combination | Dose range (mg/Kg) and route | Purpose | Frequency | Maximum # doses |
|---|---|---|---|---|
| Buprenorphine SR | 0.5 – 1 mg/Kg, SQ | Relieve pain/stress before pump infusion or MI surgery | 1 time | 1 |
| Isoflurane | 2%, inhale | Anesthetic treatment | 1 time before the operation | 1 |
| Ketamine/Xylazine | 100 mg/Kg for Ketamine, 10 mg/Kg for Xylazine, IP | Analgesic treatment | 1 time before harvest | 1 |

For mouse experiments, age/sex/genetic background matched mice were randomly separated into indicated groups. Animal operations, including Ang II infusion, MI surgery, and echocardiography measurement, were performed blindly by the Microsurgical Core surgeons. The Histology Core prepared heart sections. For group size justification, we have performed a power analysis using G*power version 3.1.9.6. The assumptions include the same standard variance in each study group, effect size=$\frac{\text{Difference of the means between study groups}}{\text{common standard deviation}}$, alpha level=0.05, power=0.9, and the number of study groups. The effect size for specific experiments is assumed based on similar studies or literature. As an exemplary experiment, the standard deviation for weight after MI or Ang II treatment is about 10%. The minimum difference to be considered significant is 25% in MI- or Ang II-induced cardiac hypertrophy and heart weight gain. With an overall type I error rate (alpha level) of 5%, at least 5 mice per treatment group are required to achieve 90% power to detect the difference in heart weight. In previous experiences from our Microsurgical Core, we have observed a survival rate of ~90% after the MI procedure. To offset the possible loss of one mouse per treatment, we used at least 6 mice per treatment group. In rare cases, mice might die after surgery, reducing the number of mice.

**Echocardiography**

For the Ang II infusion mouse model, M-mode short-axis echocardiographic image collection was performed using a Vevo2100 echocardiography machine (VisualSonics, Toronto, Canada) and a linear-array 40 MHz transducer (MS-550D). Heart rate was monitored during echocardiography measurement. Image capture was performed in mice under general isoflurane anesthesia with heart rate maintained at around 550-650 beats/min. The HR could vary in individual mice due to the potential effect of anesthesia or the surgeon's operation variation. LV systolic and diastolic measurements were captured in M-mode from the parasternal short axis. Fraction shortening (FS) was assessed as follows: % FS = (end diastolic diameter - end systolic diameter) / (end diastolic diameter) x 100%. Left ventricular ejection fraction (EF) was measured and averaged in both the parasternal short axis (M-Mode) using the tracing of the end diastolic dimension (EDD) and end systolic dimension (ESD) in the parasternal long axis: % EF=(EDD-ESD)/EDD. Hearts were harvested at multiple endpoints depending on the study. In addition to EF and FS, left ventricular end-diastolic diameter (LVEDD), left ventricular end-systolic diameter (LVESD), wall thickness of left ventricular anterior (LVAWT) and posterior (LVPWT) were also assessed. B-mode long axis echocardiographic imaging was performed for the MI mouse model, and left ventricle EF, FS, end systolic volume (LVESV), and end diastolic volume (LVEDV) were measured.

**Adult cardiomyocyte and cardiac fibroblasts isolation and culturing**

Langendorff perfusion system was used to isolate adult cardiomyocytes (CMs) and cardiac fibroblasts from the murine heart. Mice were fully anesthetized via intraperitoneal injection of ketamine/xylazine. Once losing pedal reflex, the mouse was secured in a supine position. The heart was excised and blood was removed using perfusion buffer. The heart was then fastened onto the CM perfusion apparatus, where perfusion was initiated using the Langendorff mode. Our Langendorff perfusion and digestion consisted

of three steps at 37°C: 4 mins with perfusion buffer (0.6 mM $KH_2PO_4$, 0.6 mM $Na_2HPO_4$, 10 mM HEPES, 14.7 mM KCl, 1.2 mM $MgSO_4$, 120.3 mM NaCl, 4.6 mM $NaHCO_3$, 30 mM taurine, 5.5 mM glucose, and 10 mM 2,3-butanedione monoxime), then switched to digestion buffer (300 U/ml collagenase II [Worthington] in perfusion buffer) for 3 mins, and finally perfused with digestion buffer supplemented with 40 $\mu$M $CaCl_2$ for 8 mins. After perfusion, the ventricle was placed in a sterile 35 mm dish with 2.5 ml digestion buffer and shredded into several pieces with forceps. 5 ml stopping buffer (10% FBS, 12.5 $\mu$M $CaCl_2$ in perfusion buffer) was added and pipetted several times until tissues dispersed readily, and the solution turned cloudy. The cell solution was passed through a 100 $\mu$m strainer. CMs were settled by incubating the cell suspension at 37°C for 30 mins. The CMs were resuspended in 10 ml stopping buffer and subjected to several steps of calcium ramping: 100 $\mu$M $CaCl_2$, 2 mins; 500 $\mu$M $CaCl_2$, 4 mins; 1.4 mM $CaCl_2$, 7 mins. Then the CMs were seeded onto a glass bottom dish (Nest Biotechnology) pre-coated with laminin (ThermoFisher). Plates were centrifuged for 5 mins at 1,000 g at 4°C to increase the adherence, cultured at 37°C for ~1 hr, and then switched to CM media (MEM [Corning] with 0.2% BSA, 10 mM HEPES, 4 mM $NaHCO_3$, 10 mM creatine monohydrate, 1% penicillin/streptomycin, 0.5% insulin-selenium-transferrin and blebbistatin) for cell culture and further treatments.

Cardiac fibroblasts (CFs) from the supernatant were pelleted for 5 mins at 1,200 rpm at 4°C. CFs were plated in 4-5 ml CF media (DMEM with 10% FBS and 1% penicillin/streptomycin) in a 60 mm plate and washed vigorously 3-5 times with 2 ml 1x PBS several times after 2-3 hrs to remove the unattached cells and debris, and replaced with fresh CF media. For CF only isolation, pre-weaned mice were fully anesthetized. The heart was directly cut into small pieces and digested in the digestion buffer for 4x 10 mins at 37°C with slow stirring, and CFs were plated the same as Langendorff isolation of CFs.

### Cell culture and transfection

NIH/3T3 cells (gift from Dr. Eric Small lab in Aab CVRI, Rochester) were cultured in DMEM supplemented with 10% bovine calf serum (VWR) and 1% penicillin/streptomycin (ThermoFisher). Primary CFs isolated from mouse hearts of both genders were cultured in DMEM supplemented with 10% FBS (ThermoFisher) and 1% penicillin/streptomycin. Primary cells were used at P0 for CF activation assays. We use the Polyjet to transfer the NIH 3T3 cells and DNA plasmids. siRNA transfection (100 nM) in primary CFs was performed using lipofectamine 3000, following the manufacturer's instructions.

### Lentivirus infection

Cardiac fibroblasts were cultured using DMEM with 10% FBS and 1% penicillin/streptomycin. When cells reached 50% in confluency, frozen stocks of overexpression lentivirus of mFam114a1 ($4.43 \times 10^8$ TU/ml) and control lentivirus ($3.59 \times 10^8$ TU/ml) were thawed on ice and mixed well. Then, 30 $\mu$l of virus particles were added to the cells along with 5 $\mu$g/ml of Polybrene. After 10 hrs of transduction, the culture medium was replaced with a fresh medium, and the plate was swirled gently and incubated at 37°C in a humidified 5% $CO_2$ incubator overnight.

### RNA isolation and RT-qPCR

For heart tissues (human and mouse) or cell samples, the RNA extraction was performed using TRIzol reagent (ThermoFisher) following instructions in the manual and used to detect the expression of specific genes. Briefly, the tissues were homogenized in TRIzol using Minilys Personal Homogenizer (Bertin Technologies) and placed on ice for 15 mins to lyse the tissue. Genomic DNA was removed using DNase I treatment followed by the phenol-chloroform-isoamyl alcohol extraction method. For the mRNA detection, 1 μg of total RNA was used as a template for reverse transcription using the iScript cDNA Synthesis Kit (Bio-Rad). RT-qPCR was performed with cDNA, primers of specific targets of interest, and IQ SYBR Green Supermix (Bio-Rad). Data were analyzed using the formula of the ΔΔC(t) method. cDNA was used for detecting the expression of Fam114a1, Agtrap, At1r, and the marker genes, Nppa, Nppb, Col1a1, and Col3a1. 18S rRNA or Gapdh was used as a normalization control for mRNA expression. The SYBR Green primer sequences or the Taqman probes are listed below.

SYBR green qPCR procedure: 1) initial denaturation at 95°C for 60 seconds. 2) 40 cycles of denaturation at 95°C for 10 seconds and annealing/extension at 60°C for 45 seconds. 3) melt curve analysis by 0.5°C increments at 5 seconds/step between 65-95°C. Taqman assay qPCR procedure: 1) initial denaturation at 95°C for 10 mins. 2) 40 cycles of denaturation at 95°C for 15 seconds and annealing/extension at 60°C for 60 seconds.

qPCR primers used in this study (SYBR green and Taqman probes):

| **SYBR Green RT-qPCR primers** | | | |
|---|---|---|---|
| **Species** | **Target** | **Forward Primer (5'-3')** | **Reverse Primer (5'-3')** |
| Human | 18S rRNA | TGACTCAACACGGGAAACCTC | CATGCCAGAGTCTCGTTCGTT |
| Human | COL1A1 | CGCCATCAAGGTCTACTGCAA | CTCGCTTCCGTACTCGAAC |
| Human | COL3A1 | TAAAATTCTGCCACCCCGAAC | TGCACCAGAATCTGTCCAC |
| Mouse | Fn1 | TCCTGTCTACCTCACAGACTAC | GTCTACTCCACCGAACAACAA |
| Mouse | Myh6 | GGAGGAGTATGTTAAGGCCAAG | CATCACCTGGTCCTCCTTTATG |
| Mouse | Myh7 | CCACCCAAGTTCGACAAGAT | AAGAGGCCCGAGTAGGTATAG |
| Mouse | Gapdh | AACAGCAACTCCCACTCTTC | CCTGTTGCTGTAGCCGTATT |
| Mouse | Ccna2 | CTTCACCAGACCTACCTCAAAG | CTGGTGGGTTGAGAAGAGAAA |
| Mouse | Ccne1 | CTGGACTCTTCACACAGATGAC | GCCTATCAACAGCAACCTACA |
| Mouse | Il-6 | GTCTGTAGCTCATTCTGCTCTG | GAAGGCAACTGGATGGAAGT |
| Mouse | Tnf-α | CTACCTTGTTGCCTCCTCTTT | GAGCAGAGGTTCAGTGATGTAG |
| Mouse | Nox-2 | ATGAGTTCCACACCTTCCTTC | GGCTTGAGACAACCTGGTATTA |
| Mouse | Nox-4 | CCAGAATGAGGATCCCAGAAAG | GGTAGAAGCTGTAACCATGAGG |
| Mouse | Nfatc3 | CCTCCATTAGACTGGCCTTTAC | CTCGGCTACCTTCAGTTTCATAA |
| **Taqman probes** | | | |
| **Species** | **Gene** | **Assay ID.** | **Company** |
| Human | COL1A1 | Hs00164004_m1 | ThermoFisher Scientific |
| Human | COL3A1 | Hs00943809_m1 | ThermoFisher Scientific |
| Human | GAPDH | Hs02786624_g1 | ThermoFisher Scientific |
| Human | ACTB | Hs01060665_g1 | ThermoFisher Scientific |
| Mouse | RN18S | Mm03928990_g1 | ThermoFisher Scientific |
| Mouse | Nppa | Mm01255747_g1 | ThermoFisher Scientific |
| Mouse | Nppb | Mm01255770_g1 | ThermoFisher Scientific |
| Mouse | Myh6 | Mm00440359_m1 | ThermoFisher Scientific |
| Mouse | Myh7 | Mm01319006_g1 | ThermoFisher Scientific |

| | | | |
|---|---|---|---|
| Mouse | Gapdh | Mm99999915_g1 | ThermoFisher Scientific |
| Mouse | Actb | Mm00607939_s1 | ThermoFisher Scientific |
| Mouse | Ctgf | Mm01192931g1 | ThermoFisher Scientific |
| Mouse | Fam114a1 | Mm1211743_m1 (Set-1) | ThermoFisher Scientific |
| Mouse | Fam114a1 | Mm004471784_m1 (Set-2) | ThermoFisher Scientific |
| Mouse | Fam114a1 | Mm00471781_m1 (Set-3) | ThermoFisher Scientific |
| **Genotype Primers** | | | |
| Mouse | Fam114a1 | TTCACACTGGGCCAAGTCCATAG | GGTGGTTCAGCGTTTTGAATCTG |
| | | AACACCTCCATTACTGTGGTTCC | CGCTGACTTGAACAATGTCCTTG |

### Immunofluorescence staining of Heart tissue sections and CFs

Mice were sacrificed and hearts were immediately removed, washed in ice-cold PBS, fixed in 10% neutral buffered formalin, and processed for paraffin-embedded sections in the Histological Core of Aab CVRI. Tissue sections were cut at a cross-section of 5 μm thickness 250 μm intervals were used for immunohistochemical analysis and to quantify the scar area. For immunofluorescence, paraffin-embedded slides were deparaffinized in a series of xylenes followed by 3 mins of incubations in 100% ethanol, 95% ethanol, and then placed in a distilled water. Antigen retrieval was performed in citric acid buffer (pH 6.0), followed by quenching in 3% $H_2O_2$ in PBS for 30 mins at RT. Sections were blocked in blocking buffer (2% BSA, 0.5% Triton X-100, 5% goat serum) for 2 hrs. Then slides were incubated with primary antibodies (as listed in the antibody table) overnight at 4°C. After primary antibody incubation, slides were washed in 1x PBS followed by the secondary antibody (AlexFluor-488 or AlexFluor-594 conjugated) incubation in blocking solution for 1 hr at room temperature (RT). Slides were then washed with 1x PBS (3x 5 min) and mounted with DAPI (Vectorlabs), covered by coverslips and air-dried (or kept in PBS buffer inside before imaging). The images were obtained using the Olympus FV1000 confocal microscope and the intensity was measured by NIH Image J software. Four sections for each condition were used, and for each section 5-7 randomized fields of images were captured (4 sections x 7).

### Picrosirus Red staining

Paraffin-embedded heart tissue sections were deparaffinized, and the sections were incubated with Picrosirius red reagent (Abcam) for 1 hr at RT. Slides were then washed with 1% acetic acid followed by 100% ethyl alcohol, and mounted with a mounting medium. Images were captured using the Prime Histo XE Slide Scanner (Carolina), and the fibrosis area was measured by Image J software (NIH, USA).

### Wheat germ agglutinin (WGA) staining and phalloidin staining

WGA staining was used to quantify the size of CMs in the murine heart. Deparaffinization, antigen retrieval, and quenching of auto-fluorescence were performed as described above. Heart tissue sections of WT and *FAM114A1*$^{-/-}$ mice from different treatments were probed with 10 μg/ml WGA-Alexa Fluor-488 (ThermoFisher) to stain the cardiomyocyte membrane for 1 hr at RT and followed by 3x 5 mins washes with 1x PBS. The slides were covered by coverslips with antifade solution (containing DAPI) for imaging. Cardiomyocytes were measured in the whole heart of a vehicle and Ang II infused mice and in remote and border zone areas of hearts of MI mice. The images were taken in the

fluorescence microscope, and cross-sectional areas were quantified and measured using Image J Version 2.0. software (NIH, USA) using the hand drawing tool to outline the myocytes. Myocyte size was taken from images of at least 3-4 fields per heart, and in total, 300-400 cells were measured.

For primary mouse CM cells, Alexa Fluor™ 594 Phalloidin (ThermoFisher Scientific, Cat. No. A12381) was used to measure the cell size following the instruction from the manual. Primary CM cells were treated with 10 mM ISO for 24 hrs for measuring cell size using Phalloidin. Additionally, primary CM cells were treated with 200 nM Ang II for 48 hrs for cell surface area measurement using Phalloidin staining. Cultured CMs were fixed using a 4% paraformaldehyde in PBS for 10 mins, washed with PBS, and permeabilized using 0.2% Triton X-100 for 10 mins. Cells were blocked in 2% BSA/PBS for 1 hr and stained with Alexa Fluor™ 594 Phalloidin in 1:1000 dilution for 30 mins at RT. The stained cells were gently washed with PBS for 3x 5 mins, and the slides were mounted using a mounting medium with DAPI.

**Terminal deoxynucleotidyl transferase dUTP nick end labeling (TUNEL) staining**

The tissue sections were washed with PBS twice and fixed using 4% paraformaldehyde for 20 mins. Cells were permeabilized with 0.5% of Triton X-100 for 5 mins, and incubated in a TUNEL reaction mixture (In Situ Cell Death Detection Kit; Sigma, 11684795910) for 1 hr at 37°C in the dark. Finally, cells were washed with PBS for 3x 5 mins, air dried, and mounted with DAPI-containing antifade medium. Images were captured using a BX51 microscope (Olympus).

**Trypan blue staining**

Isolated primary adult CMs from mice were cultured in 40-mm glass dishes, treated with 100 nM of Ang II for 48 hrs, and stained with Trypan blue for the assessment of cell viability of CMs. CM viability was analyzed by trypan blue dye exclusion assay. In brief, CM cultured medium was removed, and 0.04% (w/v) of trypan blue solution (VWR) was added for incubation at RT for 3-4 mins. The dead CMs appeared blue. CMs were visualized under the microscope. For each experiment, ~150-200 CMs were analyzed from different fields and dishes. CMs excluded from the trypan blue dye were considered to be viable cells. The percentage of viability was calculated.

**Cardiac fibroblast activation assay**

Adult cardiac fibroblasts were isolated from ~2-3 months old mice of WT and *FAM114A1*$^{-/-}$ and placed in 35 mm glass-bottom dishes. After 2 hrs, attached cells were washed with 1x PBS 3 times, changed to fresh CF culturing medium (DMEM containing 10% FBS and 1% penicillin/streptomycin), and cultured at 37°C for 12 hrs (or overnight). Then, cells were treated with serum starvation for 12 hrs, followed by stimulation with Ang II (1 μM) for 24 hrs. Immediately cells were fixed with 4% paraformaldehyde (PFA) for immunofluorescence staining and/or lysed in TRIzol reagent for RNA isolation and to detect the gene expression levels of myofibroblast activation markers by RT-qPCR. Cardiac fibroblast cells were isolated from WT mouse hearts and cultured for 24 or 48 hrs. Cells were fixed with 4% PFA and stained with vimentin, CD31, and CD45 antibodies

to check isolated CF cell purity. No significant contamination of endothelial cells or immune cells was noticed.

**Cardiac fibroblasts migration assay**

To determine the cardiac fibroblasts migration ability, 5x $10^4$ primary isolated ACFs from WT and FAM114A1$^{-/-}$ were plated per well 24 well plates. Fibroblasts were stimulated with human angiotensin II (100 nM) or unstimulated (vehicle). Fibroblast monolayers were then scratched with a 200 µl pipet tip. To analyze the migration of the fibroblasts, the same scratched area was captured with the Olympus microscope after 0, 6,12, and 24 hrs. The migration rate was calculated as (cell-free area at 0 hr – cell-free area at 6,12 or 24 hr) / cell-free area at 0 hr. The cell-free area was calculated using the Image J software (NIH, USA). The migration assay was carried out in 3 biological replicates with 3 wells per treatment condition.

**Triphenyl Tetrazolium Chloride (TTC) staining for measurement of infarct size**

Excised hearts were perfused with 1x PBS to remove the blood and sectioned into 4-5 levels (2 mm thick). The sliced hearts were placed in a petri dish with 1% TTC in 1x PBS and incubated for 15 mins at 37°C. Then, tissue slices were fixed with 10% formalin for 1 hr. Hearts were visualized using a bright field microscope. Quantification of infarct size was performed using Image J Version 2.0. software (NIH, USA) by normalizing total scar (white color area) to the left ventricle wall (% LV free wall) and averaging across four cross-sectional levels of the heart (apex to ligature).

**Mouse Cytokine Array**

Blood was collected from the mice through the cardiac puncture and placed in collection tubes coated with EDTA (Mini Collect, Greiner Bio-One). The samples were centrifuged at 1,200 rpm for 10 mins, and serum was collected from each sample and stored at -80°C until ready to use. A total of 100 µl of serum was used by combining serum from 5 animals from each treatment of WT and *FAM114A1*$^{-/-}$ mice. Proteome profiler mouse cytokine array (ARY006, R&D Systems) assay was performed according to the manufacturer's instructions. Cytokines were detected by chemiluminescence with exposure up to 1-5 mins using the ChemiDoc Imaging System (Bio-Rad). The relative intensity of the signals was calculated using Image Lab software (Bio-Rad).

**Immunoprecipitation and Western blot analysis**

Heart tissues, isolated CMs, and cultured CFs were homogenized in ice-cold RIPA lysis buffer with protease inhibitor cocktails (Santa Cruz). Cell debris was removed by centrifugation for 10 mins at 10,000 rpm, 4°C. Total protein concentration was determined by Bradford assay (Bio-Rad). An equal amount of protein was loaded onto 10% and 12% SDS-PAGE gels and then transferred to PVDF membranes. The membranes were blocked in the 5% milk in PBST buffer for 1 hr at RT. The respective membranes were probed with specific primary antibodies for target proteins in 4% BSA (Sigma Aldrich) in PBST buffer overnight. After several washes with the PBST buffer, the blots were incubated with a horseradish peroxidase-conjugated secondary antibody in 3% milk with

PBST buffer for 1 hr and developed using the ECL reagent (Bio-Rad). The IP experiments were repeated 3 times, and one representative image was shown.

**Stranded mRNA Library Construction & Sequencing**

Total RNA extracted from CF cells isolated from WT and *Fam114a1*$^{-/-}$ male mice were treated with DNase I (NEB) to remove potential genomic DNA in the RNA samples. The DNase I treated RNA samples were purified with phenol:chloroform:isoamyl alcohol. Total RNA concentration was determined with the NanopDrop 1000 spectrophotometer (NanoDrop, Wilmington, DE), and RNA quality was assessed with the Agilent Bioanalyzer (Agilent, Santa Clara, CA). The TruSeq Stranded mRNA Sample Preparation Kit (Illumina, San Diego, CA) was used for next-generation sequencing library construction per the manufacturer's protocols. Briefly, mRNA was purified from 200 ng total RNA with oligo-dT beads and fragmented. First-strand cDNA synthesis was performed with random hexamer priming, followed by second-strand cDNA synthesis using dUTP incorporation for strand marking. End repair and 3' adenylation was then performed on the double stranded cDNA. Illumina adaptors were ligated to both ends of the cDNA, purified by gel electrophoresis and amplified with PCR primers specific to the adaptor sequences to generate cDNA amplicons of approximately 200-500 bp in size. The amplified libraries were hybridized to the Illumina single end flow cell and amplified using the cBot (Illumina, San Diego, CA). Single end reads of 100 nt were generated for each sample using Illumina's HiSeq2500v4.

**RNA-Seq data processing and alignment**

RNA-Seq was conducted at the Genomic Research Center of URMC. Raw reads generated from the Illumina HiSeq2500 sequencer were demultiplexed using bcl2fastq version 2.19.0. Quality filtering and adapter removal were performed using Trimmomatic version 0.36(3) with the following parameters: "TRAILING:13 LEADING:13 ILLUMINACLIP: adapters.fasta:2:30:10 SLIDINGWINDOW:4:20 MINLEN:15". Processed/cleaned reads were then mapped to the *Mus musculus* reference genome (GRCm38, mg38) with STAR_2.5.2b(4) given the following parameters: "—twopassMode Basic --runMode alignReads --genomeDir $(5) --readFilesIn ${SAMPLE} --outSAMtype BAM SortedByCoordinate --outSAMstrandField intronMotif --outFilterIntronMotifs RemoveNoncanonical". The subread-1.5.0(6) package (featureCounts) was used to derive gene counts given the following parameters: "-s 2 -t exon -g gene_name" and the gencode M12 gene annotations. Differential expression analysis and data normalization were performed using DESeq2-1.16.1(7) with an adjusted p-value threshold of 0.05 within an R-3.4.1(8) environment. A batch factor was given to the differential expression model to control batch differences. Gene ontology and KEGG pathway enrichment analyses were performed using DAVID Bioinformatics Resources 6.8(9).

**Statistical Analysis**

All quantitative data were presented as mean±SEM and analyzed using the GraphPad Prism (9.0.0) software. For normally distributed data, an unpaired two-tailed Student *t*-test was performed to compare two groups and one-way or two-way ANOVA with Tukey's multiple comparisons test for the comparisons between more than three groups.

For data that is not normally distributed, a non-parametric Mann-Whitney test was performed for the comparisons between two groups and Kruskal-Wallis test with Dunn's multiple comparisons test for the comparisons between more than three groups. Statistical significance was assumed at a value of $P<0.05$.

## SI References


1. Forrester SJ, Booz GW, Sigmund CD, Coffman TM, Kawai T, Rizzo V, et al. Angiotensin II Signal Transduction: An Update on Mechanisms of Physiology and Pathophysiology. *Physiol Rev.* 2018;98(3):1627-738.
2. Wu J, Subbaiah KCV, Xie LH, Jiang F, Khor ES, Mickelsen D, et al. Glutamyl-Prolyl-tRNA Synthetase Regulates Proline-Rich Pro-Fibrotic Protein Synthesis During Cardiac Fibrosis. *Circ Res.* 2020;127(6):827-46.
3. Bolger AM, Lohse M, and Usadel B. Trimmomatic: a flexible trimmer for Illumina sequence data. *Bioinformatics.* 2014;30(15):2114-20.
4. Dobin A, Davis CA, Schlesinger F, Drenkow J, Zaleski C, Jha S, et al. STAR: ultrafast universal RNA-seq aligner. *Bioinformatics.* 2013;29(1):15-21.
5. Whiffin N, Karczewski KJ, Zhang X, Chothani S, Smith MJ, Evans DG, et al. Characterising the loss-of-function impact of 5' untranslated region variants in 15,708 individuals. *Nat Commun.* 2020;11(1):2523.
6. Liao Y, Smyth GK, and Shi W. featureCounts: an efficient general purpose program for assigning sequence reads to genomic features. *Bioinformatics.* 2014;30(7):923-30.
7. Love MI, Huber W, and Anders S. Moderated estimation of fold change and dispersion for RNA-seq data with DESeq2. *Genome Biol.* 2014;15(12):550.
8. Team RC. R: A language and environment for statistical computing. R Foundation for Statistical Computing, Vienna, Austria. URL https://www.R-project.org/. 2016.
9. Huang da W, Sherman BT, and Lempicki RA. Systematic and integrative analysis of large gene lists using DAVID bioinformatics resources. *Nat Protoc.* 2009;4(1):44-57.

# Full unedited blots for Figure 1

Figure 1B

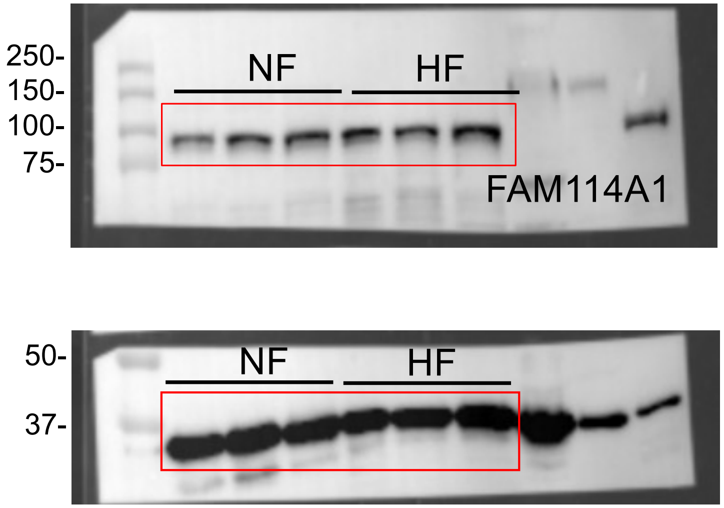


Figure 1D

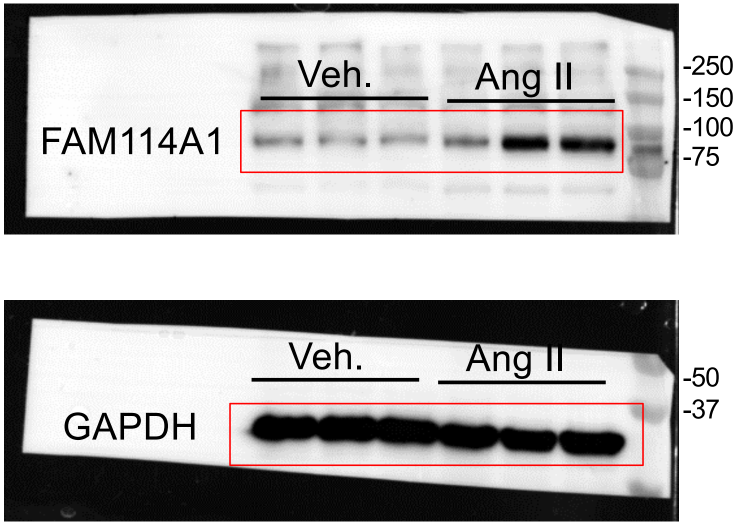

# Full unedited blots for Figure 5B

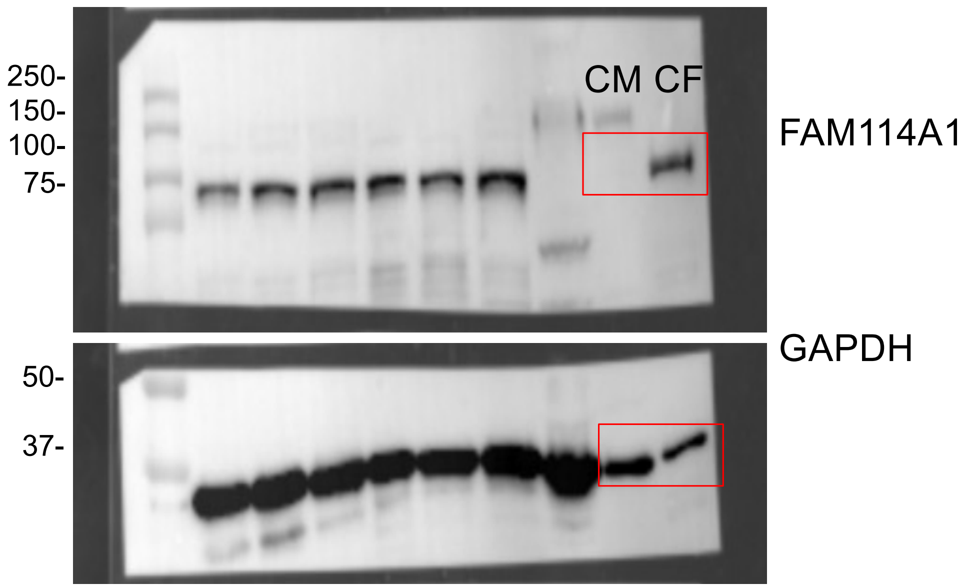

# Full unedited blots for Figure 6A

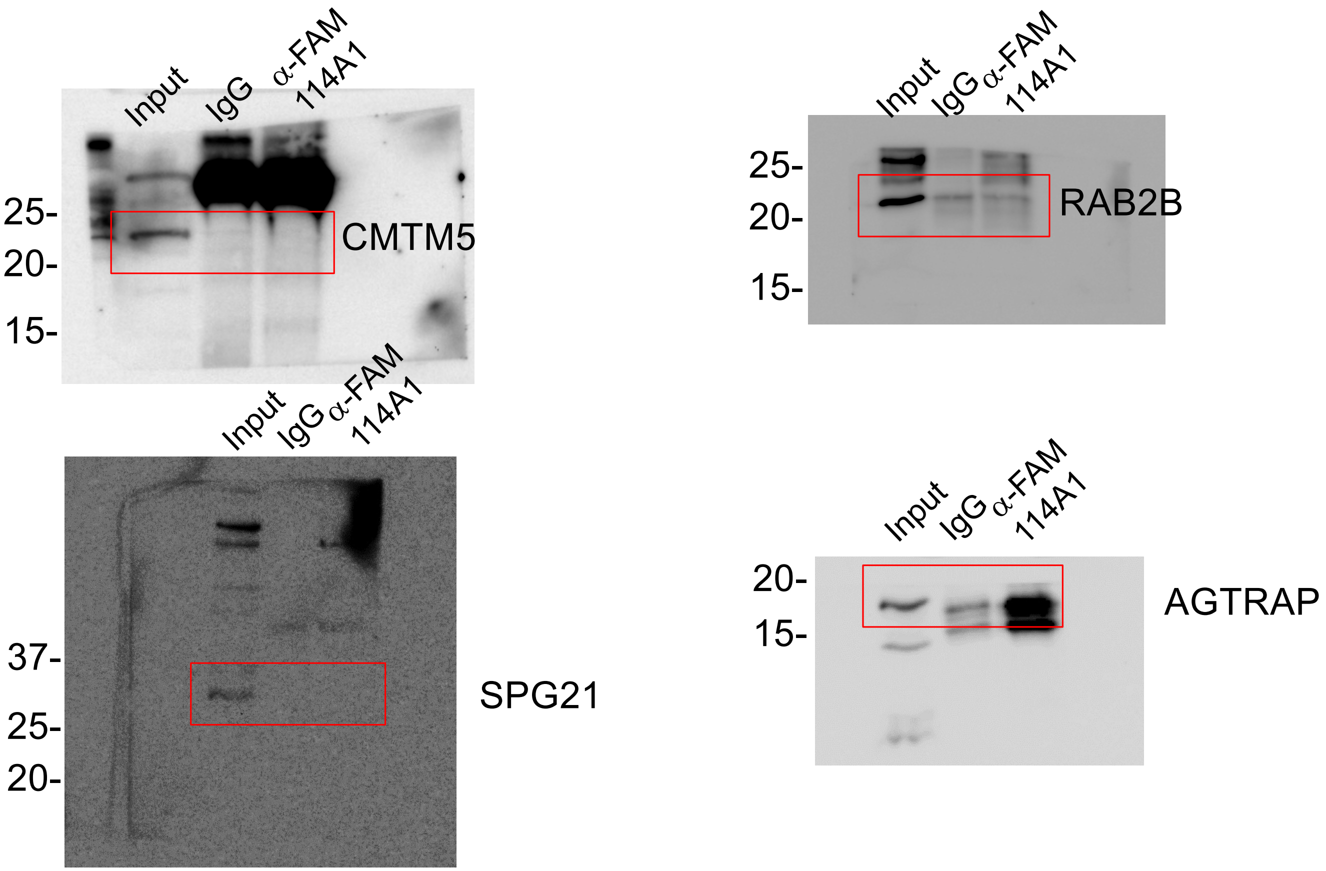

# Full unedited blots for Figure 6C

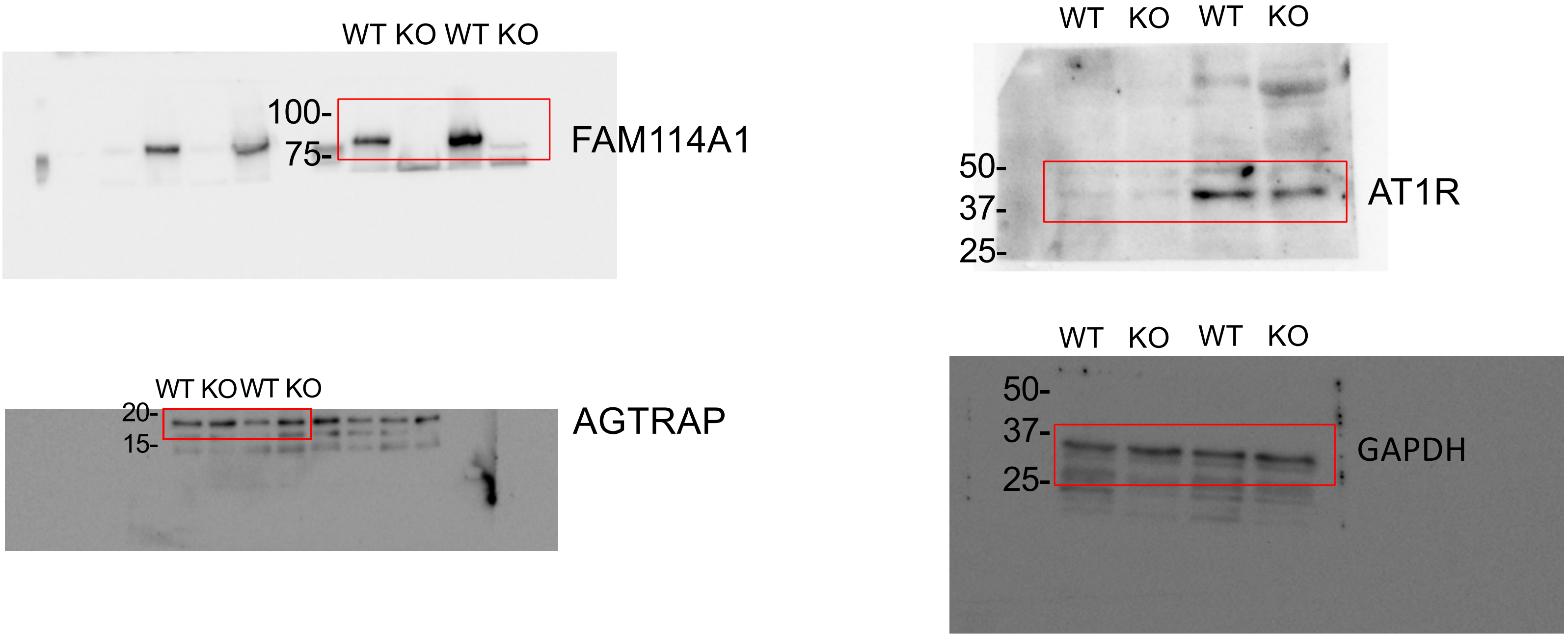

# Full unedited blots for Figure 6D

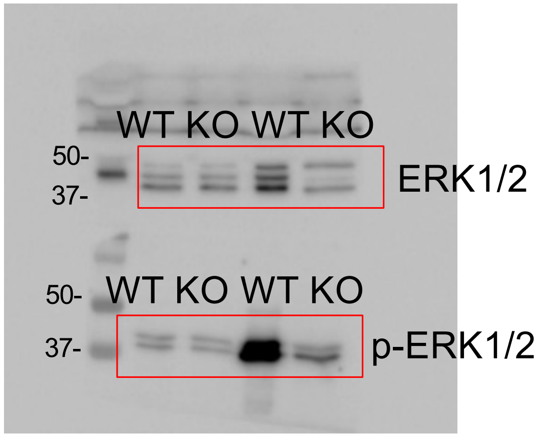


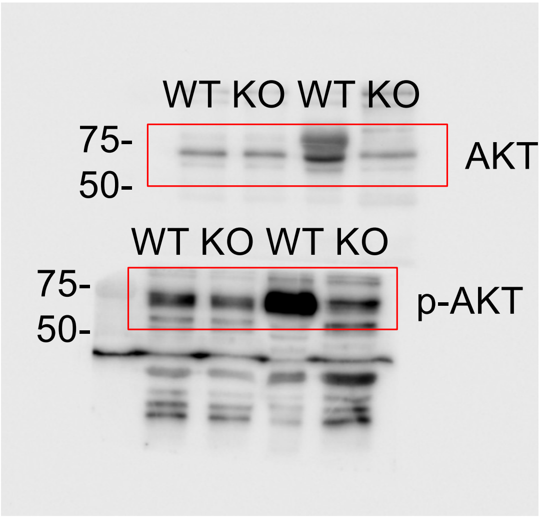


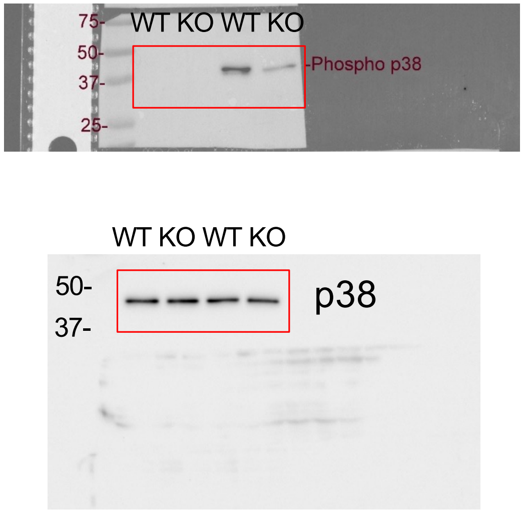

# Full unedited blots for Figure S1J

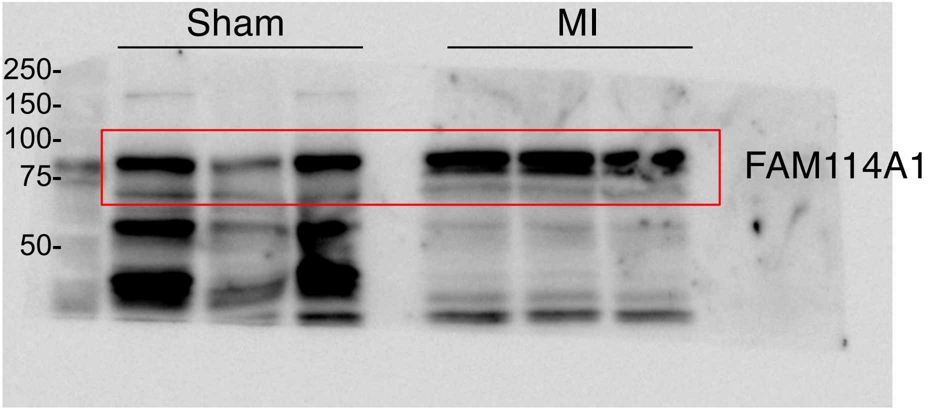


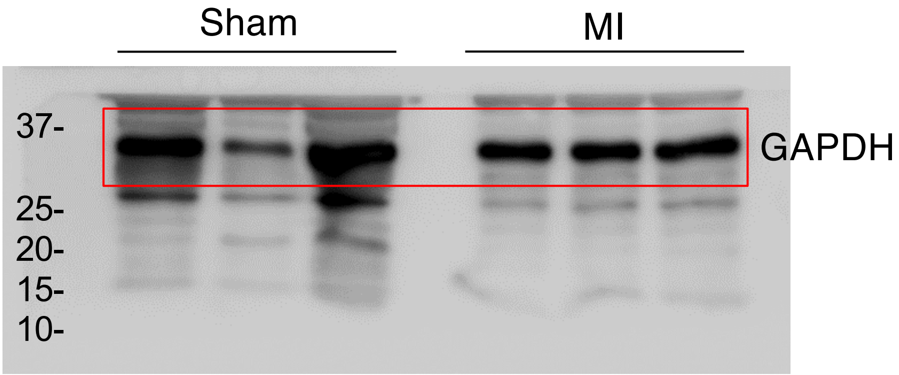


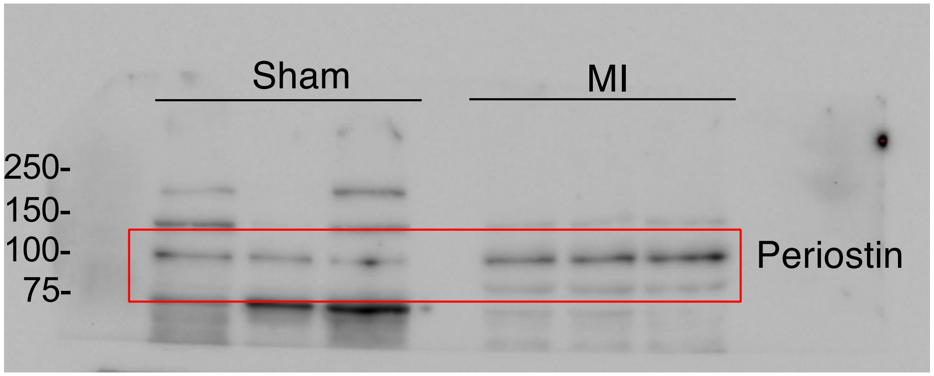

# Full unedited blots for Figure S2D

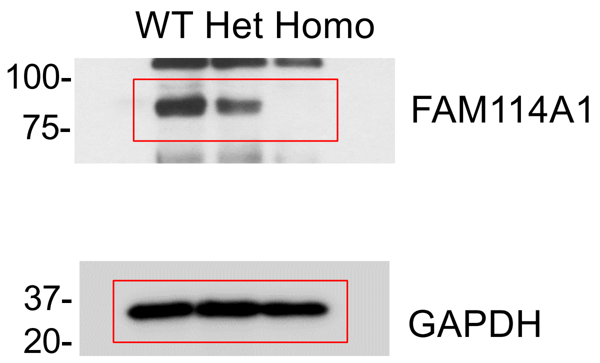

# Full unedited blots for Figure S7B

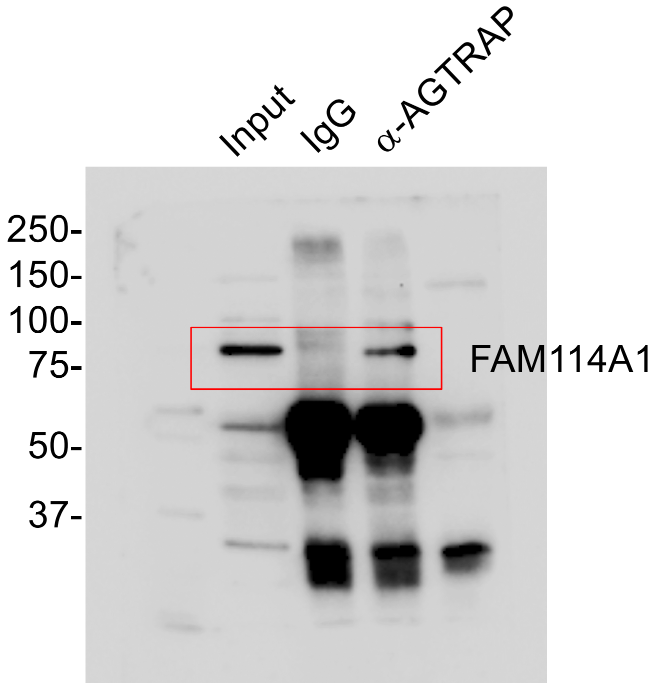


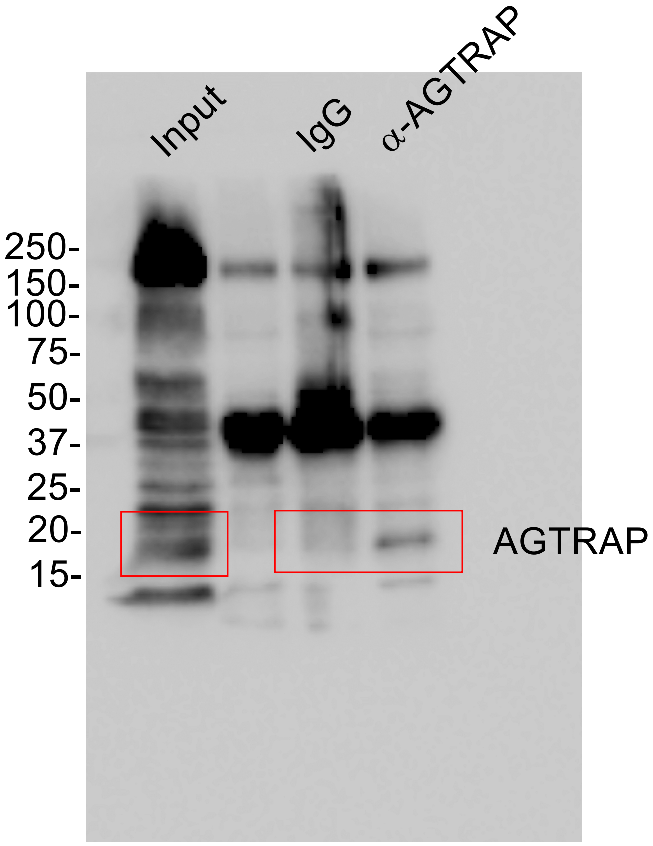

# Full unedited blots for Figure S7C

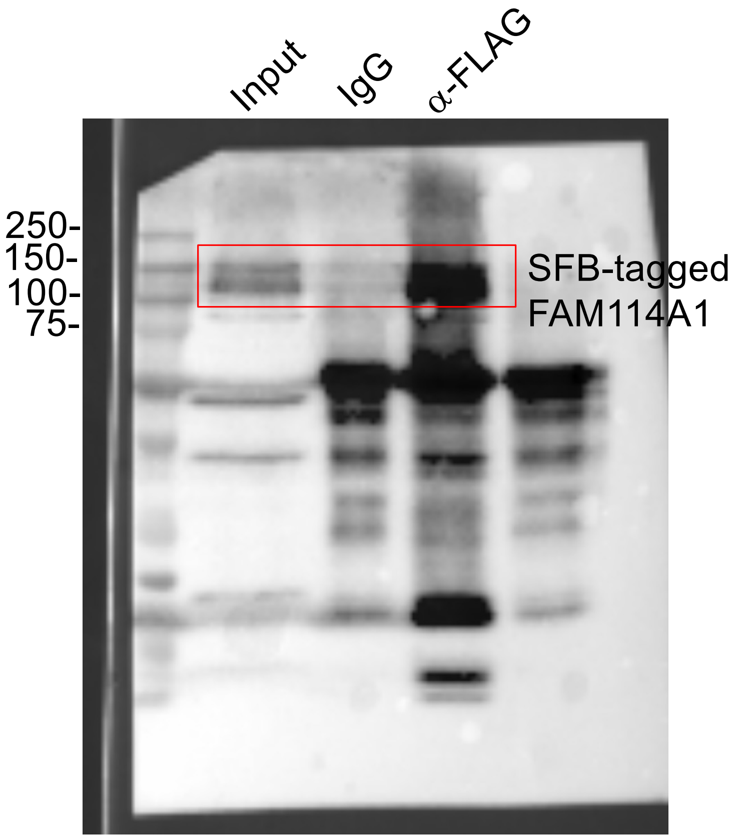


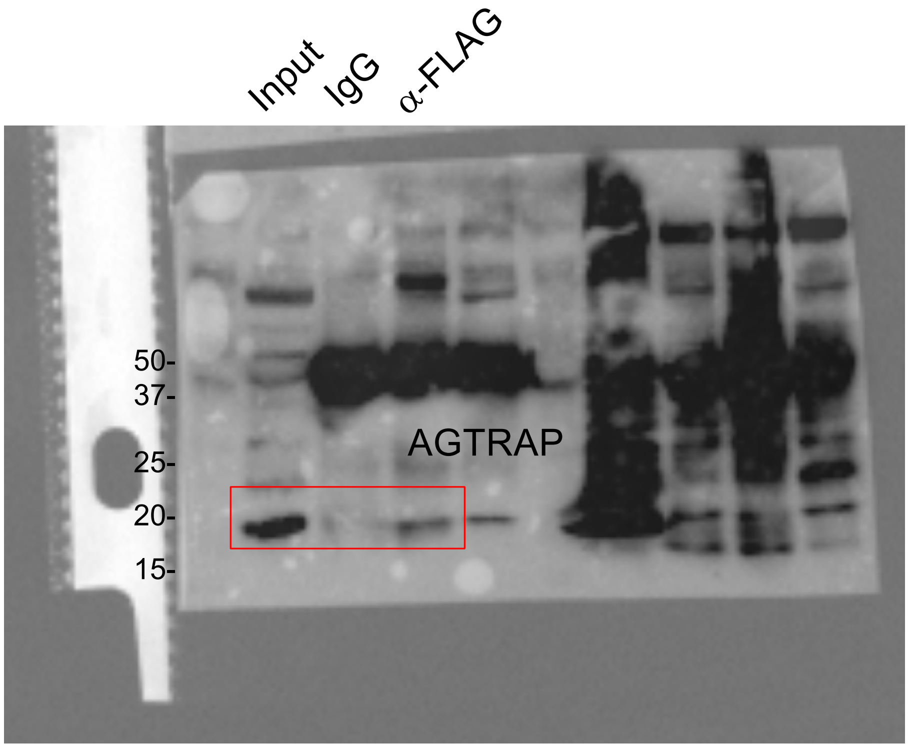

# Full unedited blots for Figure S7E

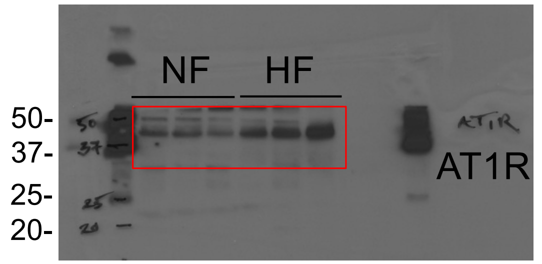


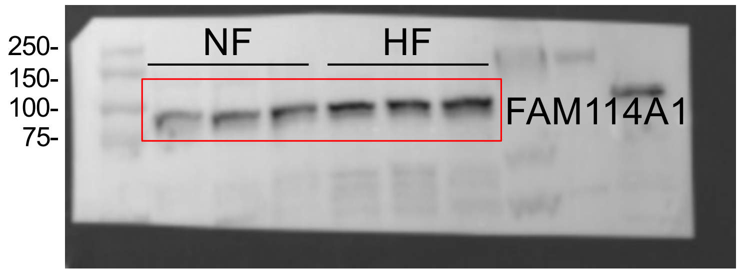


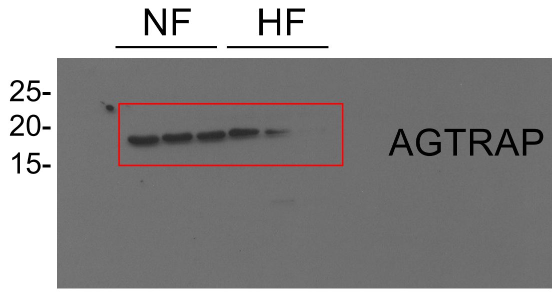


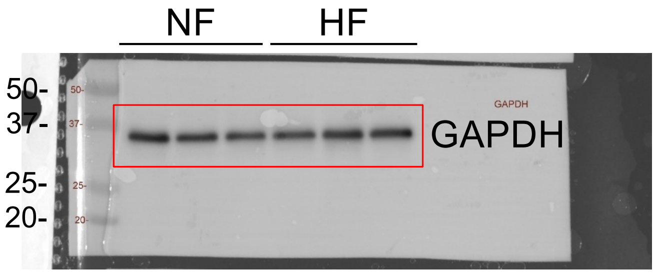

# Full unedited blots for Figure S7J

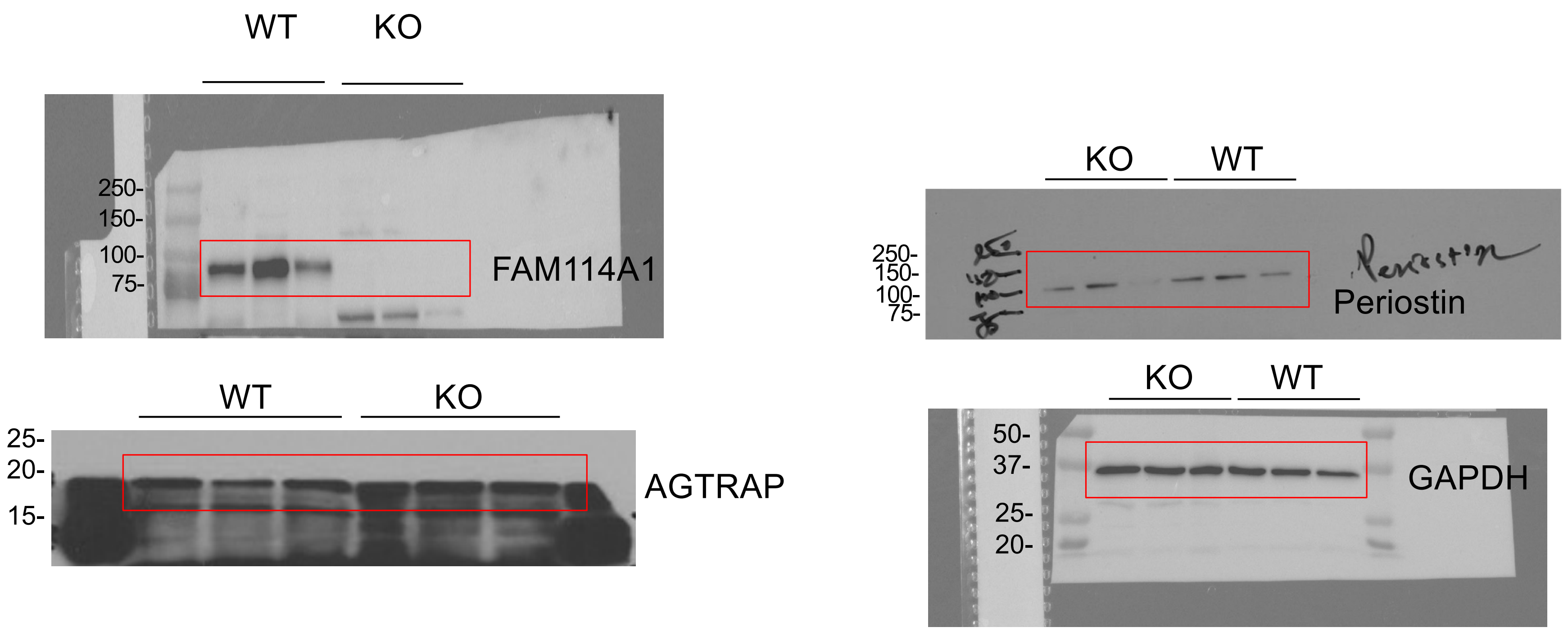

Supplement: Supplemental data [file jciinsight-7-152783-s132.pdf]
